# Novel 5-Substituted Oxindole Derivatives as Bruton’s Tyrosine Kinase Inhibitors: Design, Synthesis, Docking, Molecular Dynamics Simulation, and Biological Evaluation

**DOI:** 10.1021/acsomega.3c08343

**Published:** 2024-02-07

**Authors:** Vani Madhuri Velavalapalli, Venkatanarayana Chowdary Maddipati, Soňa Gurská, Narendran Annadurai, Barbora Lišková, Naresh Kumar Katari, Petr Džubák, Marián Hajdúch, Viswanath Das, Rambabu Gundla

**Affiliations:** † GITAM School of Pharmacy, GITAM Deemed to Be University, Hyderabad, Telangana 502329, India; ‡ Department of Chemistry, GITAM School of Science, GITAM Deemed to Be University, Hyderabad, Telangana 502329, India; § Institute of Molecular and Translational Medicine, Faculty of Medicine and Dentistry, Palacký University and University Hospital Olomouc, Hněvotínská 1333/5, Olomouc 77900, Czech Republic; ∥ Czech Advanced Technologies and Research Institute (CATRIN), Institute of Molecular and Translational Medicine, Palacký University Olomouc, Olomouc 77900, Czech Republic

## Abstract

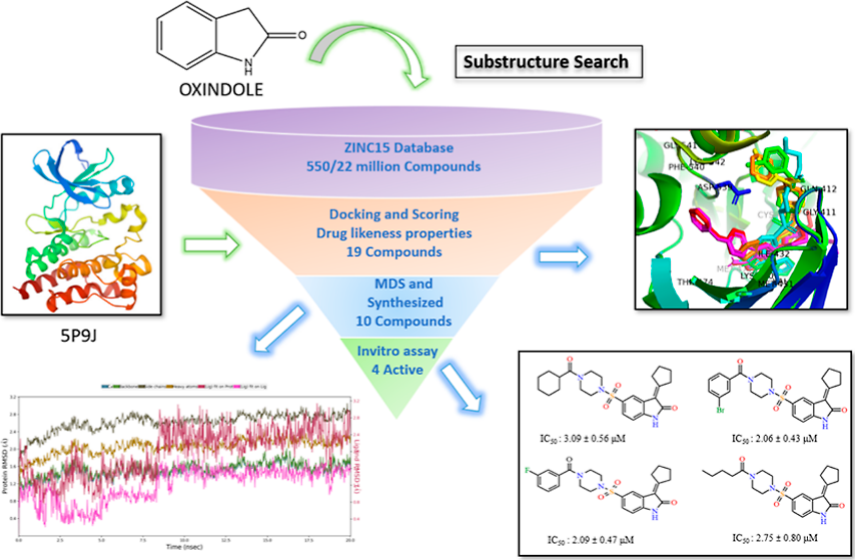

Bruton’s tyrosine kinase (BTK) is a non-RTK cytoplasmic kinase predominantly expressed by hemopoietic lineages, particularly B-cells. A new oxindole-based focused library was designed to identify potent compounds targeting the BTK protein as anticancer agents. This study used rational approaches like structure-based pharmacophore modeling, docking, and ADME properties to select compounds. Molecular dynamics simulations carried out at 20 ns supported the stability of compound **9g** within the binding pocket. All the compounds were synthesized and subjected to biological screening on two BTK-expressing cancer cell lines, RAMOS and K562; six non-BTK cancer cell lines, A549, HCT116 (parental and p53^–/–^), U2OS, JURKAT, and CCRF-CEM; and two non-malignant fibroblast lines, BJ and MRC-5. This study resulted in the identification of four new compounds, **9b**, **9f**, **9g**, and **9h**, possessing free binding energies of −10.8, −11.1, −11.3, and −10.8 kcal/mol, respectively, and displaying selective cytotoxicity against BTK-high RAMOS cells. Further analysis demonstrated the antiproliferative activity of **9h** in RAMOS cells through selective inhibition of pBTK (Tyr223) without affecting Lyn and Syk, upstream proteins in the BCR signaling pathway. In conclusion, we identified a promising oxindole derivative (**9h**) that shows specificity in modulating BTK signaling pathways.

## Introduction

**Figure 1:**
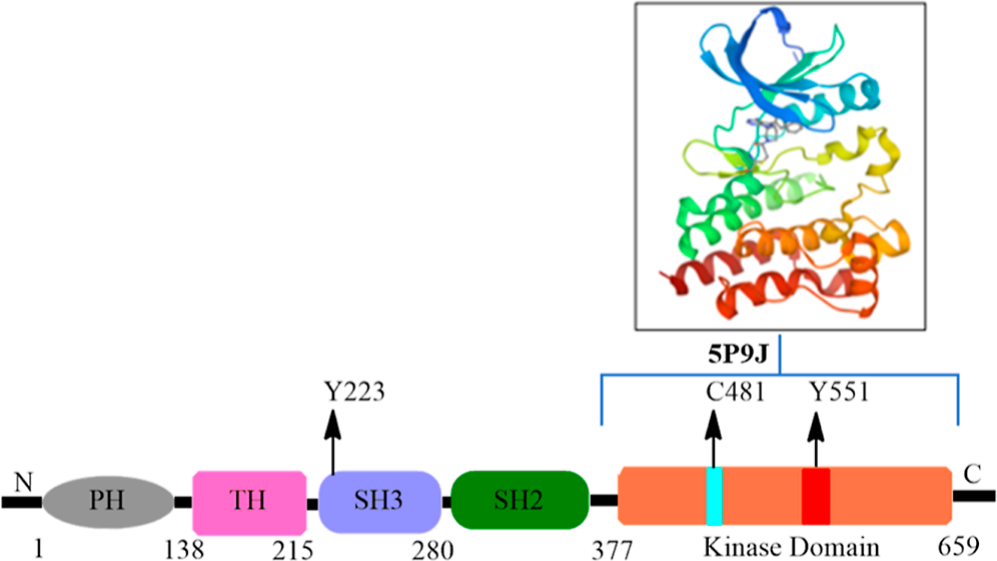
Graphical depiction of BTK showing different regions: (1) Pleckstrin homology (PH), (2) Tec homology, (3) SRC homology (SH3), (4) SRC homology (SH2), and (5) kinase domain. The most frequent C481S mutation associated with resistance to covalent BTK inhibitors is located in the kinase domain.

Bruton’s tyrosine kinase (BTK) belongs to the Tec family of kinase and is a non-RTK cytoplasmic kinase primarily expressed by hemopoietic lineages, particularly B-cells.^1^ BTK has five different domains, and two important phosphorylation sites, tyrosine 233 (Y233) and tyrosine 551 (Y551), that are important for BTK activation are located within the SRC homology and C-terminal kinase domain (Figure 1). Y551 is phosphorylated by spleen tyrosine kinase (Syk) or LYN proto-oncogene (Lyn), leading to BTK activation.^1^ BTK is also activated when PIP3/PI3K is attached to the PH domain with different cell surface receptors.^1^ BTK is implicated in various human diseases, primarily those related to the immune system, such as X-linked agammaglobulinemia, chronic lymphocytic leukemia (CLL), mantle-cell lymphoma (MCL), follicular lymphoma, non-Hodgkin’s lymphomas, Waldenström’s macroglobulinemia (WM), diffuse large B-cell lymphoma, rheumatoid arthritis, and other autoimmune diseases.^1,2^ Therefore, BTK inhibitors have implications not only in cancer but also in the treatment of severe autoimmune diseases such as multiple sclerosis.^2^

Currently, there are three covalent irreversible inhibitors of BTK available in the market, namely, ibrutinib, zanubrutinib, and acalabrutinib.^2^ Ibrutinib is approved for treating CLL, MCL, and WM,^1,2^ whereas the second-generation more selective inhibitors, acalabrutinib and zanubrutinib, are approved for treating CLL, small lymphocytic lymphoma, and relapsed/refractory MCL.^2^ However, the anticancer activity of ibrutinib, zanubrutinib, and acalabrutinib that bind to cysteine 481 in the BTK kinase domain is susceptible to resistance caused by the C481S mutation (Figure 1).^3,4^ Additionally, these drugs result in considerable toxicity after long-term use due to off-target interactions with other kinases containing cysteine motifs.^4^ There is a growing interest in non-covalent, reversible BTK inhibitors, such as fenebrutinib and vecabrutinib, to overcome drug resistance and minimize off-target effects.^3,4^ Nonetheless, there remains substantial interest in BTK as a molecular target in medicinal chemistry, with numerous active molecules currently in various stages of drug discovery and development.^4−15^

**Figure 2:**
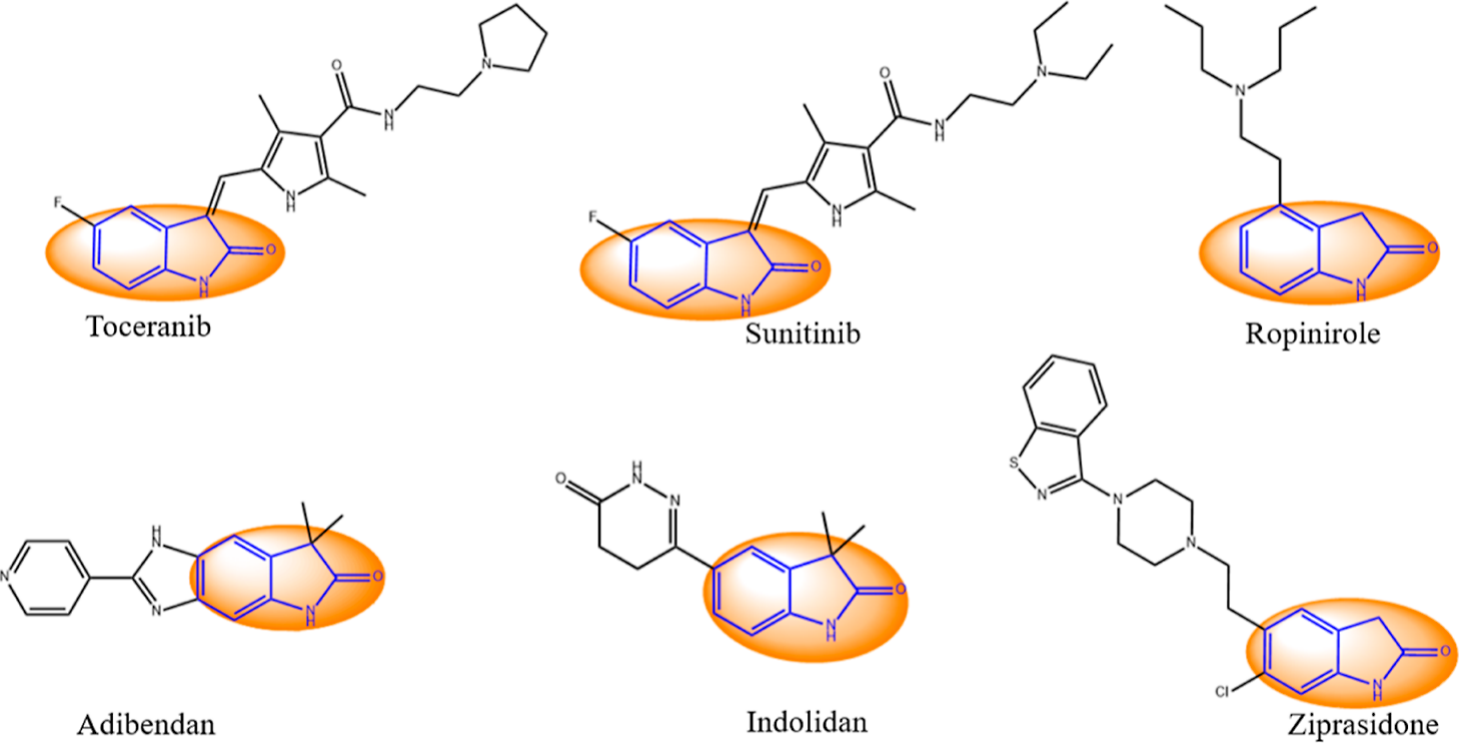
Structures of commercially marketed drugs containing oxindole as the core moiety approved for treating canine and human tumors, Parkinson’s disease in humans, and heart conditions in dogs, hypertension, schizophrenia, and bipolar disorder.

Oxindoles are significant structural motifs in various natural products and pharmaceutical compounds, making them valuable in drug discovery.^16,17^ Compounds containing the oxindole structure are known for their anticancer, anti-inflammatory, antiviral, and antifungal activities.^17,18^ Importantly, substituted oxindole derivatives exhibit selectivity toward several protein kinases, such as VEGFR-1, VEGFR 2, VEGFR 3, PDGFRα, PDGFRβ, Kit, Flt-3, and CSF-1R.^17^ The U.S. Food and Drug Administration-approved drugs such as sunitinib and toceranib used for treating human and canine tumors contain indoline-2-one, which is crucial for their pharmacological activity as tyrosine kinase inhibitors.^16−18^ Several other oxindole derivatives, such as ropinirole, adibendan, indolidan, and ziprasidone, have been successfully developed as marketed drugs for various medical conditions (Figure 2).^16−18^ Our recent research on BTK inhibitors uncovered the notable selective cytotoxicity of oxindole sulfonamides against BTK-high human B-cell lymphoma cells, with no-to-minimal cytotoxicity in non-BTK cancer and non-cancer cells.^19^ In this study, we report the design, synthesis, and biological evaluation of a series of novel oxindole derivatives as promising anti-BTK candidates.

## Results and Discussion

**Figure 3:**
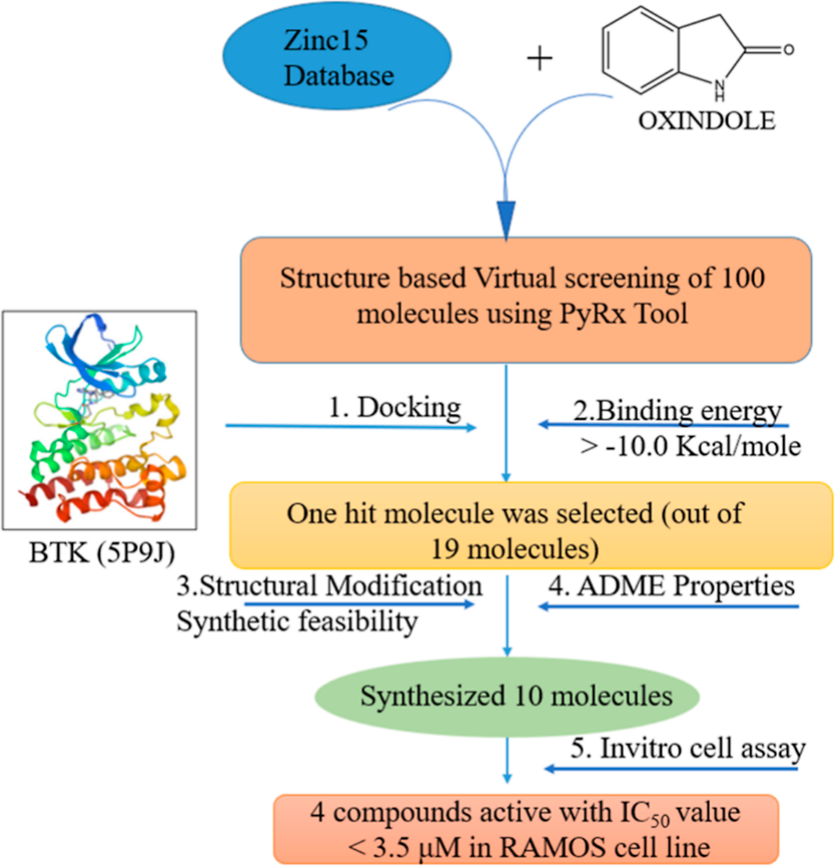
Schematic representation of workflow of the study.

**Figure 4:**
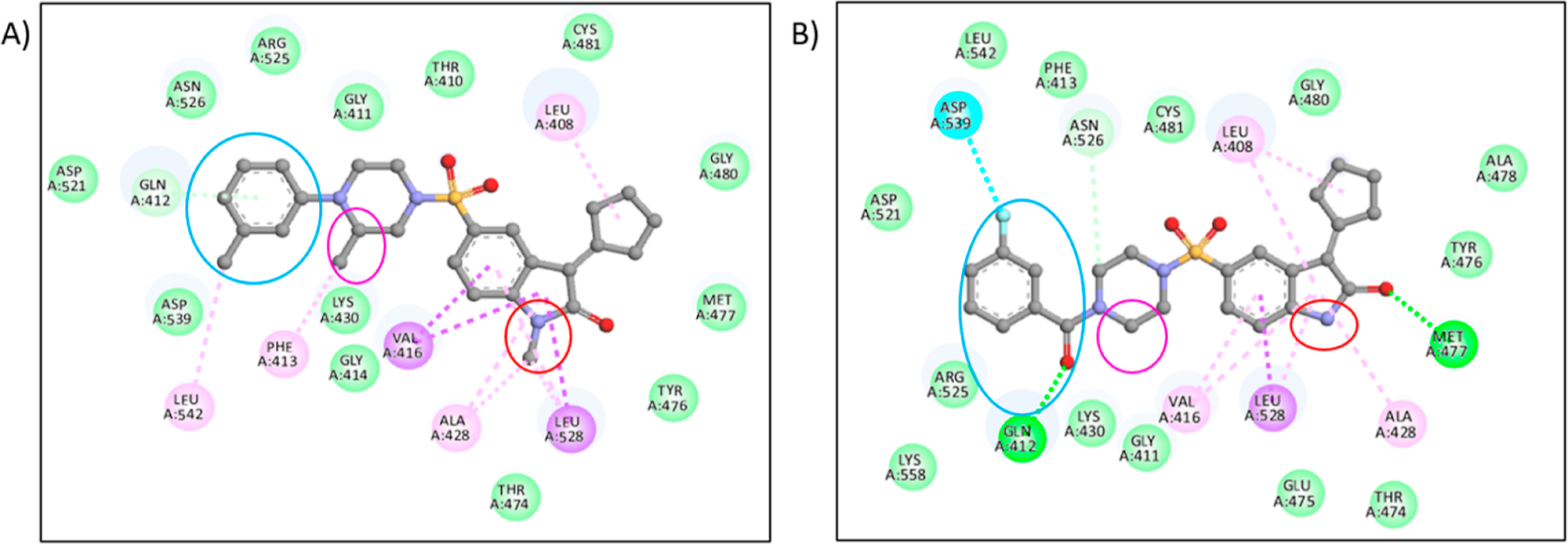
Structure-based pharmacophore modeling of compound **819** (A) to **9g** (B) for better interaction; the blue color ring shows the replacement of methyl benzyl with 3-fluoro benzoyl, the pink color ring shows the replacement of the methyl group with hydrogen, and the red color ring shows the replacement of the methyl group with hydrogen. (A) Interaction profile of compound **819**. (B) Interaction profile of **9g**.

**Scheme 1:**
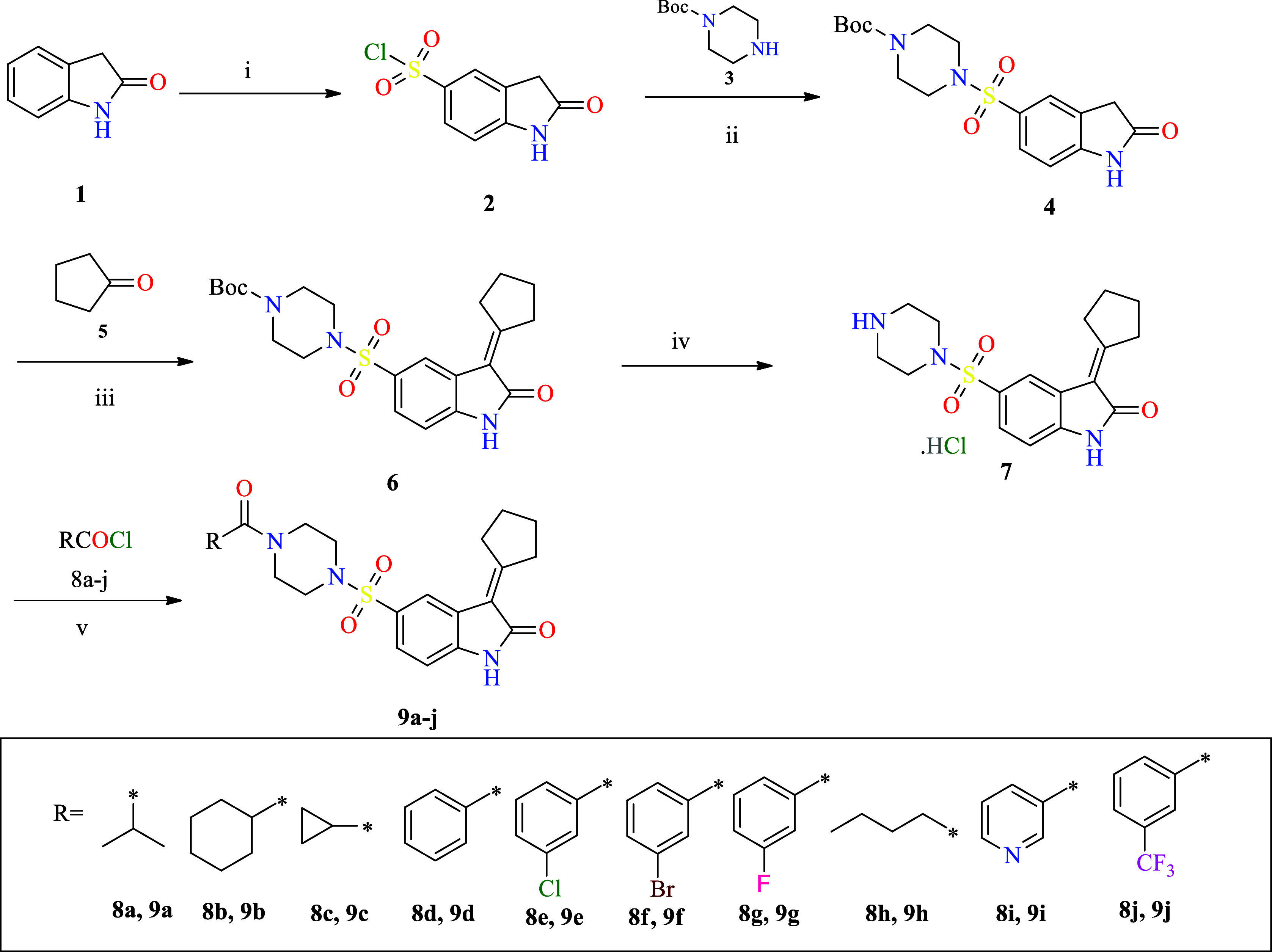
Synthesis of 5-Substituted Oxindole Derivatives; Conditions: (i) ClSO_3_H, 70 °C, 2 h; (ii) Pyridine, 1,4-Dioxane, rt, 2 h; (iii) Piperazine, EtOH, rt, 2 h; (iv) 4 M HCl in 1,4-Dioxane, rt, 16 h; and (v) DIPEA, CH_2_Cl_2_, rt, 3 h

Structure-based designing was carried out to generate anticancer compounds against BTK protein. The compound selection process is depicted as a flowchart shown in Figure 3. Initially, compound **819** was identified as the lead compound from the docking based on the binding free energy, and further modifications were carried out to increase its interactions with the critical amino acids in BTK (PDB-5P9J), as shown in Figure 4. For the purpose of establishing a structure–activity relationship, acid chlorides were introduced at position R (Scheme 1), leading to the synthesis of 10 compounds. It is worth noting that all the 10 compounds (**9a**–**9j**) exhibited favorable ADME properties, as detailed in Table 1.

**Table 1:**
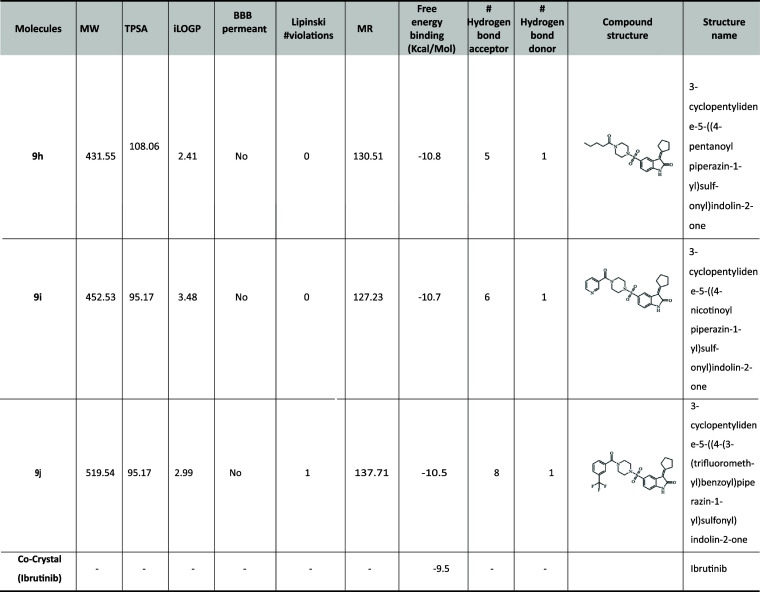
ADME Properties of the 10 Compounds from Swiss ADME, along with the Docking Score

**Figure 5:**
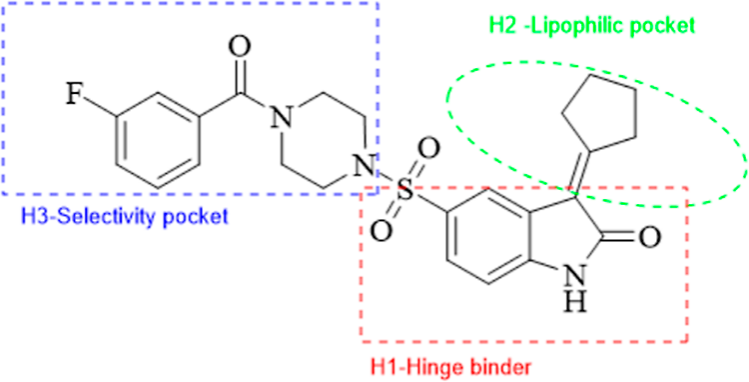
Schematic representation of the interaction of BTK with **9g**.

The methyl benzyl group attached to the piperazine ring was replaced with the different acid chlorides, which showed interaction with ASP-539 amino acid and GLN-412. Replacing the methyl group with hydrogen in the piperazine ring and the oxindole ring methyl group with hydrogen showed better interaction with MET-477 and LEU-528 at the base of the ATP pocket (Figure 4). Amino acid MET-477 forms a hydrogen bond with the carbonyl oxygen of the oxindole ring, and LEU-528 forms a π–σ bond with the phenyl of the oxindole ring in the H1 pocket (Figure 5). Amino acids VAL-416, ALA-428, and LEU-408 form alkyl and π–alkyl bonds with an oxindole ring in the H1 pocket. Other amino acids CYS-481, GLY-480, ALA-478, TYR-476, THR-474, and GLU-475 form van der Waals interactions with the cyclopentylidene moiety in the H2 lipophilic pocket. Amino acid ASN-526 forms a carbon–hydrogen bond with the piperazine ring, oriented toward the bottom of the H3 pocket (Figure 5). In the H3 pocket, we changed 10 different acid chloride substitutions and synthesized analogues with docking scores of more than −10 kcal/mol.

In compound **9b**, the cyclohexane group showed van der Waal interaction with ASP-521, ARG-525, ASN-526, TYR-551, and LYS-558 amino acids; and a π–σ bond with VAL-416; and alkyl interactions with LEU-408, LEU-528, and ALA-428. The hydrogen bonds are formed with GLN-412 and MET-477 with 2.89 Å, and the free binding energy is −10.8 kcal/mol. In compound **9f**, the bromine group showed π–alkyl interaction with PHE-413, alkyl interaction with LEU-542, and van der Waal interaction with ASP-539 amino acid. Similarly, hydrogen bonds are formed with GLN-412 with 2.94 Å and MET-477 with 3.18 Å and a free binding energy of −11.1 kcal/mol. For compound **9g**, the fluorine group forms a π–σ bond with DFG residue ASP-539 in the H3 pocket, an essential amino acid (Figure 5). Similarly, the hydrogen bonds are formed with GLN-412 with 2.91 Å and MET-477 with 3.01 Å, and the free binding energy is −11.3 kcal/mol.

**Figure 6:**
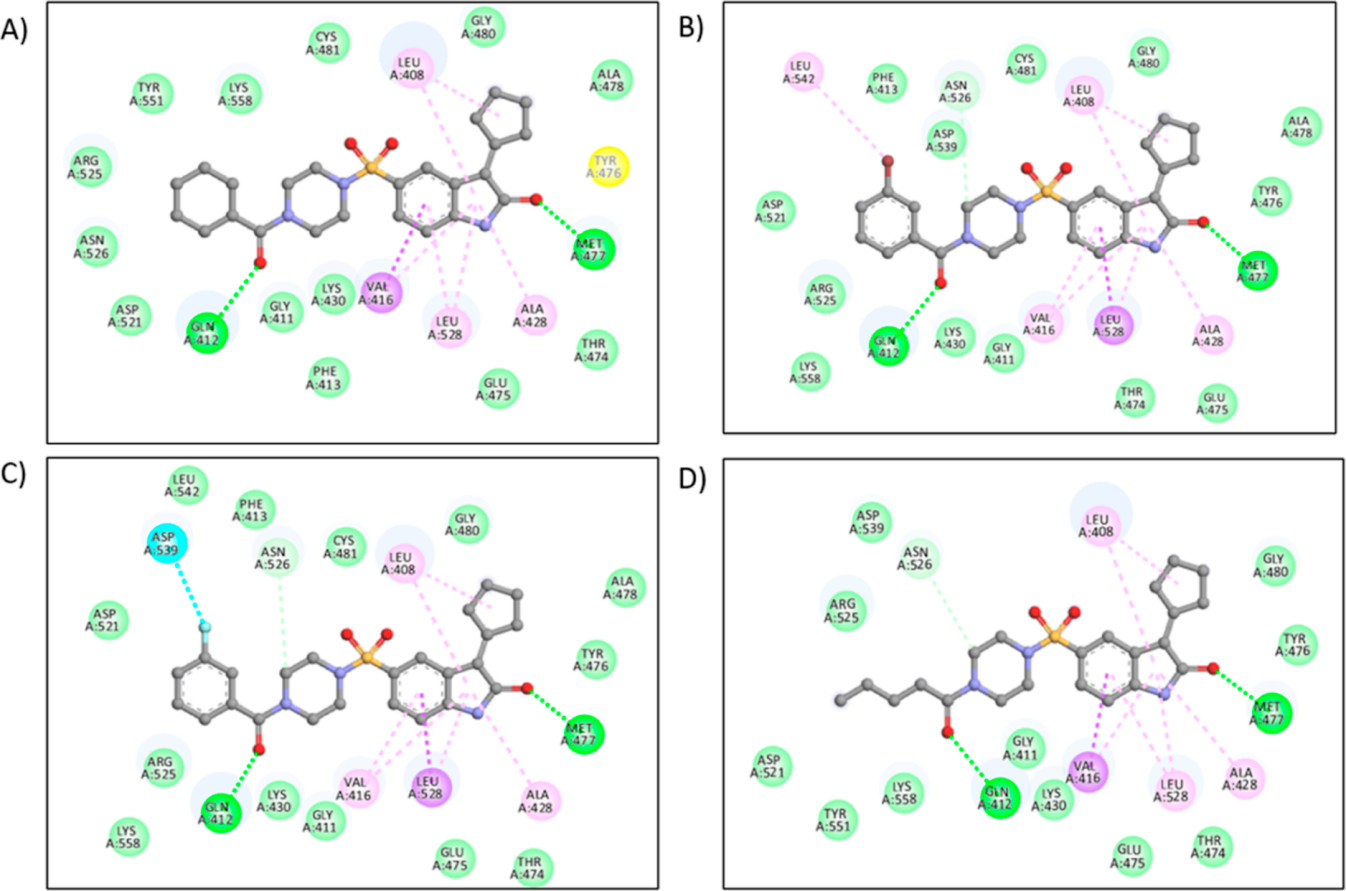
Active site of BTK (PDB-ID-5P9J) showing interactions with compounds (A) **9b**, (B) **9f**, (C) **9g**, and (D) **9h** with amino acids GLY-411, ASP-539, MET-477, VAL-416, and LYS-430.

**Figure 7:**
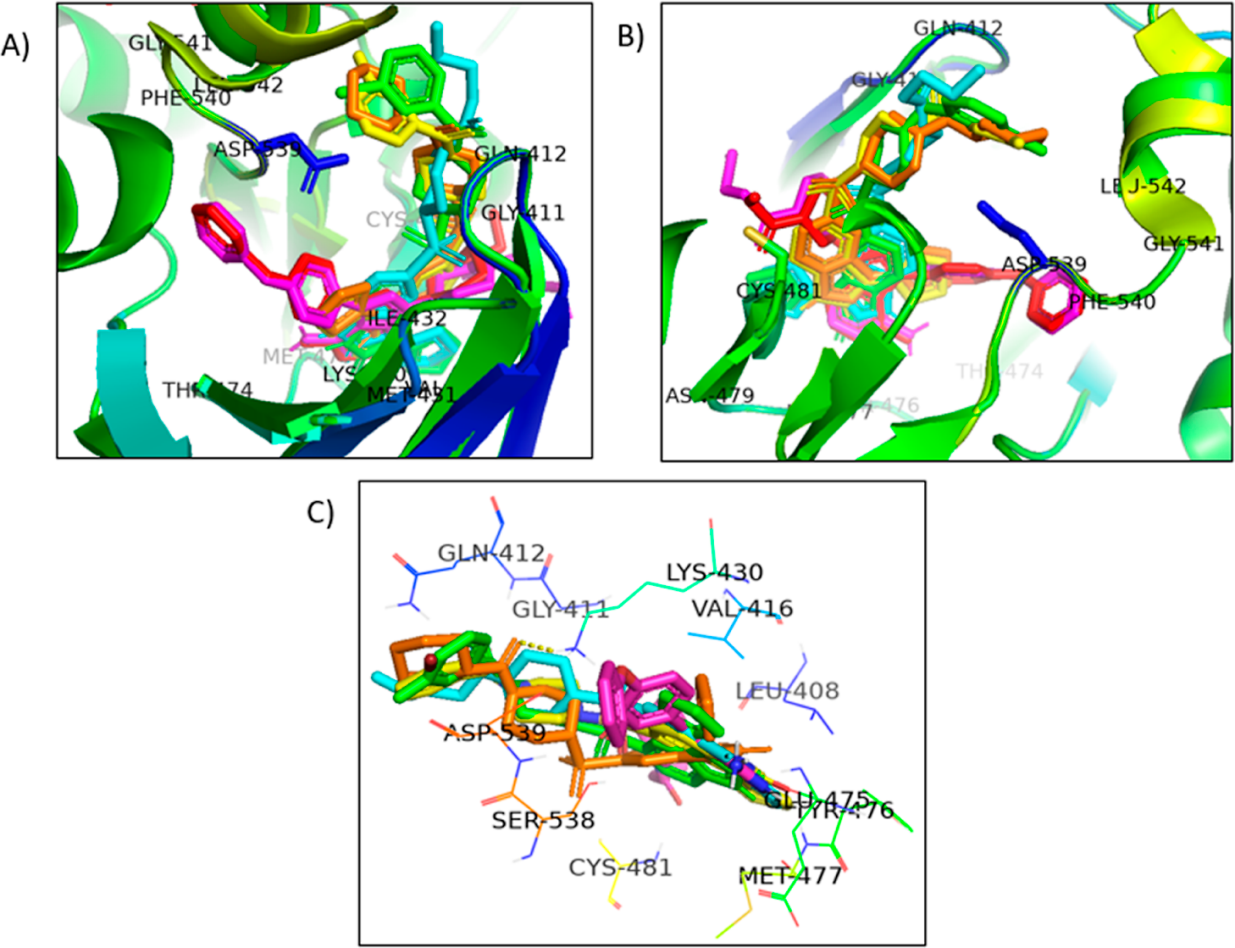
(A) Docking poses of four compounds **9b**—orange, **9f**—yellow, **9g**—green, and **9h**—cyan along with the cocrystal structure—pink and the redocked cocrystal structure—red at the same root-mean-square deviation (RMSD). (B,C) Active site images of compounds **9b**—orange, **9f**—yellow, **9g**—green, and **9h**—cyan with the cocrystal—pink.

In compound **9h**, the aliphatic chain showed van der Waal interaction with only ASP-521, ARG-525, TYR-551, and LYS-558 amino acids; π–σ bond with VAL-416; and alkyl interactions with LEU-408, LEU-528, and ALA-428. The hydrogen bonds are formed with GLN-412 with 2.93 Å and MET-477 with 3.21 Å, as shown in Figure 6, and the free binding energy is −10.8 kcal/mol. The crystal structure of BTK complexed with the cocrystal structure with four compounds, **9b**, **9f**, **9g**, and **9h**, is shown in Figure 7.

## Molecular Dynamics Simulations

**Figure 8:**
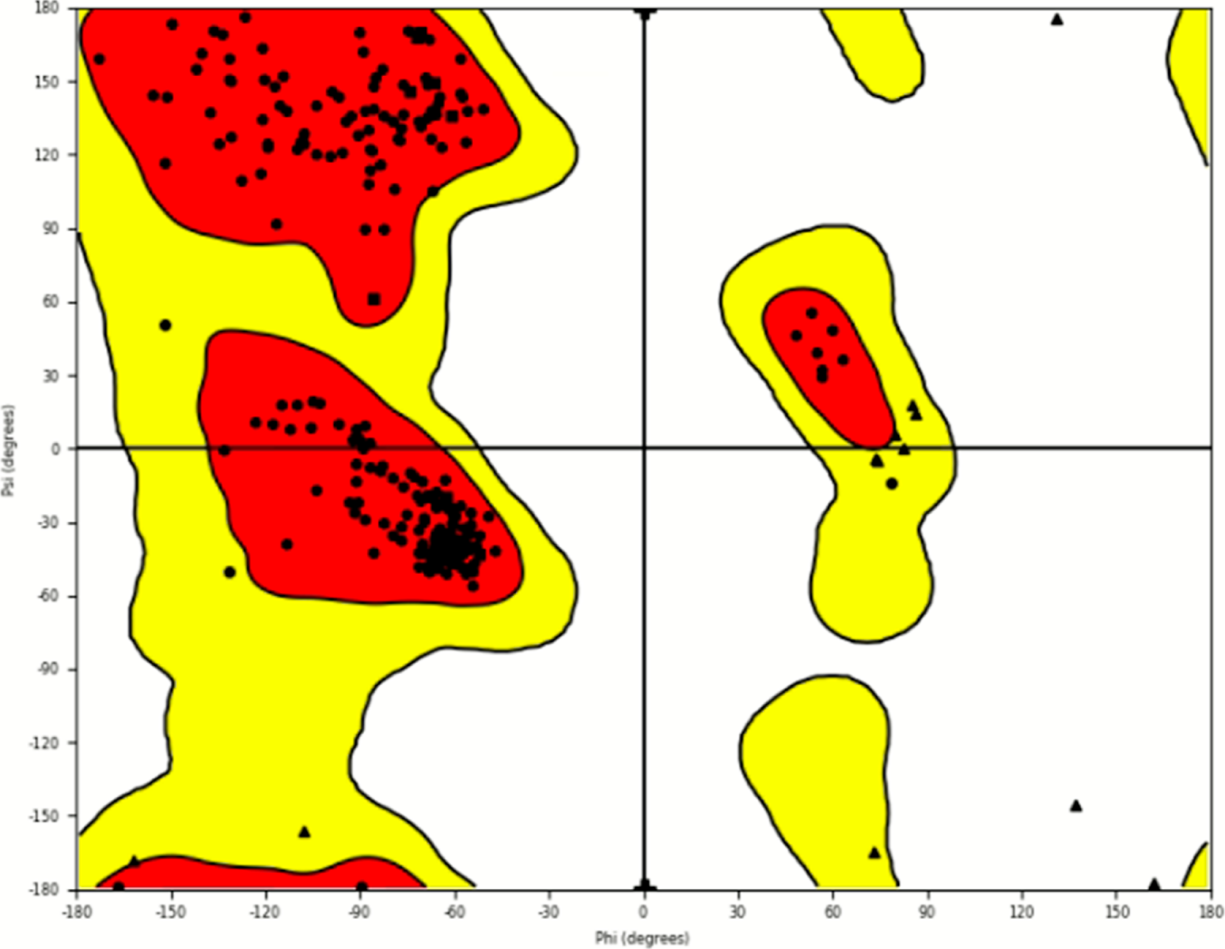
Ramachandran plot depicting stereochemical geometry for compound **9h**-5P9J complex.

Molecular dynamics (MD) simulations at 50, 20, and 20 ns were performed for complex compounds **9f**-5P9J, **9g**-5P9J, and **9h**-5P9J. Several factors, such as the ligand protonation state, conformation of ligands, water molecules, cofactors, ions, and conformational and solvation entropies, will affect docking predictions in an unexpected pattern. Many reports support the role of MD simulations in filtering docking results.^20,21^ MD simulations were carried out to determine the interaction stability of the ligand–protein docked complex. The stereochemical solid geometries of the residues were analyzed for the final structure using the Ramachandran map (Figure 8). The residue percentage in the favored region is 95.69% (267 residues), allowed is 3.58% (10 residues), and the outlier is 0.71% (2 residues).

**Figure 9:**
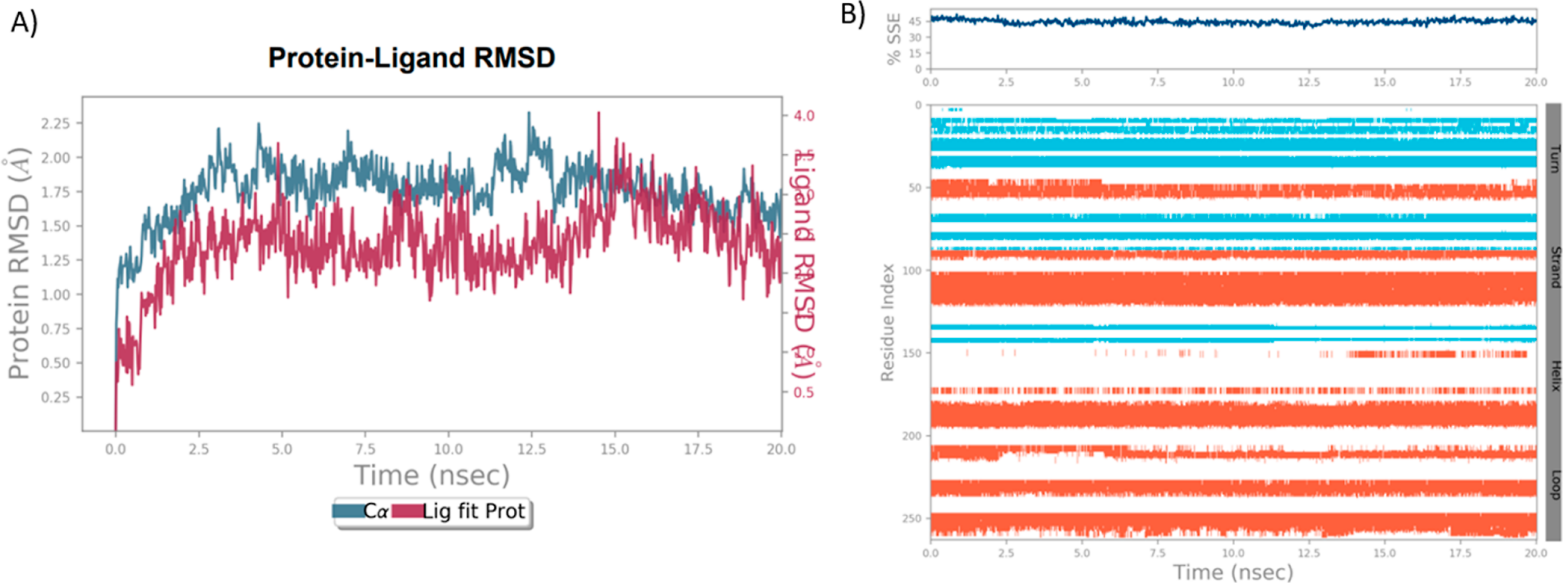
(A) RMSD plot of protein (5P9J) and ligand (**9h**). (B) SSE of the protein is shown with helices in blue and beta strands in orange.

**Figure 10:**
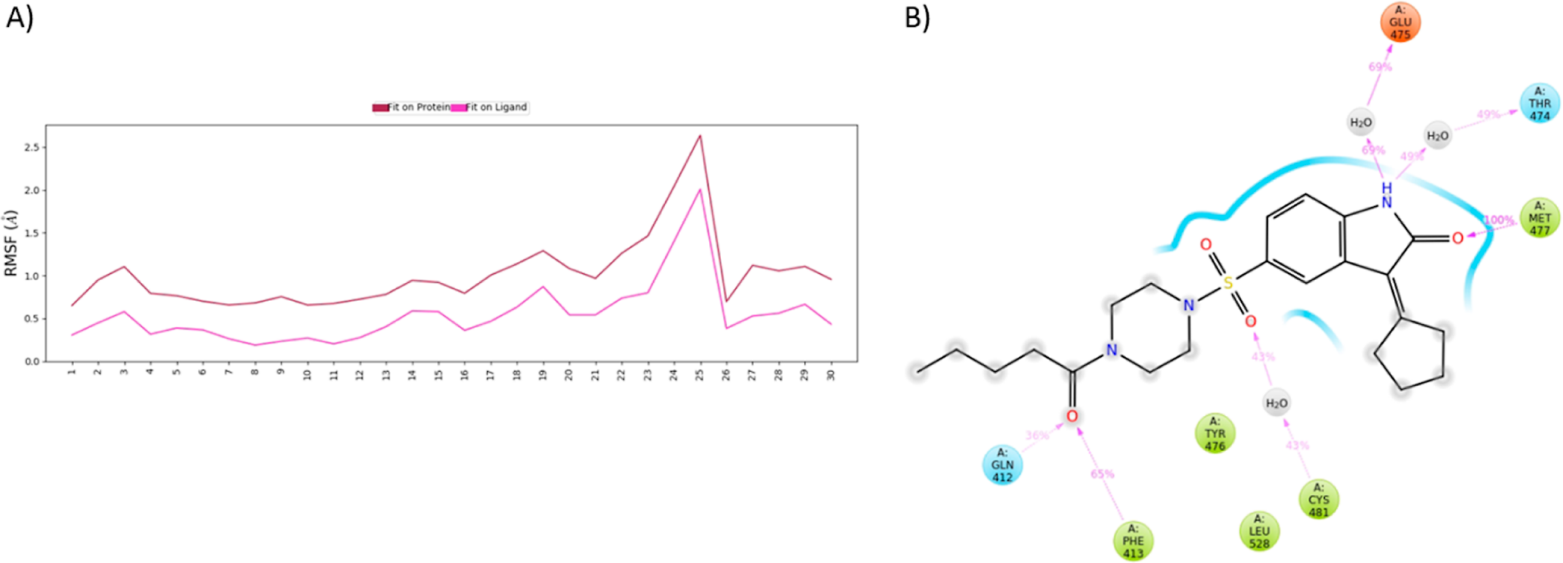
(A) Ligand-RMSF plot for compound **9h**-protein 5P9J, where the brown line indicates the ligand fluctuations regarding the binding site residues on the target protein and the pink line indicates the fluctuations where the ligand in each frame was aligned on the ligand in the first reference frame. (B) Compound **9h** shows interacting residues.

The RMSD of Cα, backbone, and side chains for all complexes showed fluctuations in the range of 0.4–3.2 Å, which are in the acceptable range. The results showed that the simulation equilibrated after 4 ns. The RMSD values of Cα and ligand for complex 5P9J-**9h** are shown in Figure 9A. The protein’s secondary structure elements (SSEs) were monitored during the simulations (Figure 9B). The total percentage of SSE for **9f**, **9g**, and **9h** was found to be 42.95, 45.71, and 44.49%, respectively. The RMSD values of Cα and ligand and the SSE for complex 5P9J-**9f** and complex 5P9J-**9g** are shown in Figures S1A,B and S2A,B, respectively. The protein-RMSF was monitored to analyze the local changes along the protein chain. The ligand-RMSF was examined to study the fluctuations at the atom level, as shown in Figure 10A. The brown line indicates “Fit on Protein”, and the pink line indicates “Fit on Ligand”. Compound **9h** interacted with GLY-412 and MET-477, making hydrogen bonds without water. In the presence of water, MET-477 bonded 100% through simulation, PHE-413 with 65%, and GLY-412 with 36%. The hydrogen water bonds formed are GLU-475 at 69%, THR-474 at 49%, and CYS-481 at 43%, as shown in Figure 10B. The results showed that the role water molecules play within the binding pocket of the protein 5P9J for compound **9h**. The percentage of contacts between compounds **9f** and **9g** with the protein are given in Figures S3A,B and S4A,B. Noticeably, both the compounds showed fewer interactions than compound **9h**. The other interacting residues of compound **9h** are LEU-408, THR-410, GLY-411, GLU-412, PHE-413, GLY-414, LYS-417, ALA-428, LYS- 430, THR-474, GLU-475, TYR-476, MET-477, CYS-481, ASN-484, ARG-525, ASN-526, CYS-527, LEU-528, VAL-537, ASP-539, VAL-546, and TYR-551 as shown in Figure S5, where the RMSF values are less than 3.6 Å. The green color indicates compound **9h**, the maroon color indicates the *B* factor, and the orange and blue bands indicate helices and β-strands, respectively. The *B* factor and Cα are parallel, indicating that the results correlate. In Figure S6, the top panel shows the total number of contacts compound **9h** made throughout the trajectory. In contrast, the bottom panel showed the specific contacts made with the protein throughout the trajectory in each frame. Compound **9h** formed a hydrogen bond with residue MET-477; hydrogen and water bridge interaction with GLN-412, PHE-413, GLU-475, and CYS-481; hydrophobic interactions with VAL-416, ALA-428, TYR-476, and LEU-528; and water bridge bonds with LEU-408, THR-410, GLY-414, THR-474, and ARG-525, as shown in Figure S7. The radial plot gave the details of torsion angle conformation at the simulation time, as shown in Figure S8. The 2D diagram of compound **9h** is presented with color-coded rotational bonds. The bar plots gave information about the torsion angle’s density and the rotational bond’s potential in kcal/mol. The torsion potential relationships of compound **9h** with conformational strain were explained, conserving a protein-bound confirmation. The stability of the ligand was analyzed using six parameters: polar surface area, RMSD, solvent-accessible surface area, molecular surface area, IntraHB, and rGyr (radius of gyration), shown in Figure S9. The RMSD of the ligand was shown to be stable, which ranged up to 1.5 Å. The histogram graphs, torsional analysis, and stability analysis of the ligands for compounds **9f** and **9g** are shown in Figures S10–S15.

## Synthesis

Commercially available oxindole was used as a starting material for synthesizing 5-substituted oxindole derivatives. To chlorosulfonic acid, oxindole was added portionwise at 0 °C and stirred for 30 min at room temperature (RT). The mixture was heated to 70 °C using an oil bath to afford 2-oxoindoline-5-sulfonyl chloride **2**.^22,23^ Sulfonyl chloride intermediate **2** was coupled in the presence of pyridine with *N*-Boc piperazine **3**,^24,25^ resulting in *N*-Boc 4-(2-oxoindoline-5-sulfonamido)piperazine **4**. Intermediate **4** in ethanol was subjected to Knoevenagel condensation with cyclopentanone **5** by adding pyrrolidine as a base that affords intermediate **6**.^26,27^ The protecting group tertiary butyl carbonyl of intermediate **6** was removed by treating it with 4 M HCl in 1,4-dioxane to yield critical scaffold **7** as HCl salt. Analogues **9a**–**9j** were synthesized by amide coupling with different acid chlorides **8a**–**8j** in the presence of di-isopropyl ethyl amine. The liberated hydrochloric acid was neutralized by adding the base di-isopropyl ethyl amine (Scheme 1).

## Cytotoxicity of Compounds in Cell Cultures

Ten derivatives were tested in a panel of cell lines consisting of ITK-positive T-cell leukemia lines,^28^ BTK-positive B-cell leukemia lines,^29^ ITK/BTK-negative malignant lines, and two non-malignant fibroblast lines. Although both RAMOS and K562 cell lines are positive for BTK expression, they do not express ITK. However, BTK expression is relatively higher in RAMOS than in K562 cells.^19^ In contrast, ITK expression is higher in JURKAT than in CCRF-CEM cells, both of which lack BTK expression.^19^ RAMOS is well known for its high BTK expression.^29^ Other panel cell lines, including A549, HCT116, U2OS, MRC-5, and BJ, do not express BTK or ITK.^19^

The compounds that did not show 50% inhibition of cell proliferation when evaluated at a single dose of 50 μM were not processed for dose–response analysis. The cytotoxicity profiling was done by considering compounds with IC_50_ values above 50 μM as inactive, above 30 μM as weakly active, between 10 and 20 μM as moderately active, and below 10 μM as highly active. Based on these standard norms, the effects of four structurally similar compounds (active—**9b**, **9f**, **9g**, and **9h**) were interesting to observe in BTK-high cell lines (Table 2). These four compounds were inactive in A549, HCT116, U2OS, JURKAT, and non-malignant cells. Of all the 10 compounds, four compounds, **9b**, **9f**, **9g**, and **9h**, showed activity in RAMOS cells.

**Table 2:** Biological Activity of 10 Compounds in ITK/BTK-Negative, ITK-Positive, and BTK-Positive Cancer Cell Lines and Non-malignant Fibroblast Lines^a^

	ITK/BTK-negative cell lines				ITK-positive cell lines		BTK-positive cell lines		non-cancer	
	A549	HCT116	HCT116p53^–/–^	U2OS	JURKAT	CCRF-CEM	RAMOS	K562	MRC-5	BJ
**9a**	>50	>50	>50	>50	>50	>50	>50	>50	>50	>50
**9b**	>50	>50	>50	>50	>50	35.53 ± 7.05	3.04 ± 0.56	>50	>50	>50
**9c**	>50	>50	>50	>50	50 ± 0	>50	>50	>50	>50	>50
**9d**	>50	>50	>50	>50	>50	>50	>50	>50	>50	>50
**9e**	>50	>50	>50	>50	>50	>50	>50	>50	>50	>50
**9f**	>50	>50	>50	>50	>50	>50	2.06 ± 0.43	>50	>50	>50
**9g**	>50	>50	>50	>50	>50	46.46 ± 5.81	2.09 ± 0.47	>50	>50	>50
**9h**	>50	>50	>50	>50	>50	46.15 ± 3.13	2.75 ± 0.80	>50	>50	>50
**9i**	>50	>50	>50	>50	>50	>50	>50	>50	>50	>50
**9j**	>50	>50	>50	23.62 ± 6.44	>50	>50	>50	>50	27.38 ± 6.80	>50
ibrutinib		30 ± 1	30 ± 1	23 ± 3	5 ± 1	4 ± 1	0.3 ± 0	27 ± 3	28 ± 0	29 ± 1

a IC_50_ values are in μM. Mean ± SD, *n* ≥ 6. Representative dose–response curves of **9b**, **9f**, **9g**, **9h**, and **9j** are shown in the Supporting Information.

The isopropyl group in compound **9a** attached to the piperazine moiety shows no activity. Replacing it with cyclohexyl moiety in compound **9b** showed promising activity in RAMOS cells with an IC_50_ value of 3.04 ± 0.56 μM. Compounds **9c**, **9d**, and **9e**, having cyclopropyl, benzoyl, and 3-chlorobenzoyl groups attached to the piperazine moiety, did not show any activity in RAMOS cells. Compounds **9f** and **9g** having 3-bromobenzoyl and 3-fluorobenzoyl groups attached to the piperazine moiety showed promising activity in RAMOS cells with IC_50_ values of 2.06 ± 0.43 μM and 2.09 ± 0.47 μM, respectively. Compound **9h** with aliphatic group replacement showed promising activity in RAMOS cells with an IC_50_ value of 2.75 ± 0.80 μM. Compounds **9i** and **9j** with pyridine and trifluoro benzoyl moieties attached to piperazine showed no activity in RAMOS cells. The SAR profiling indicates that group cyclopentylidene at the C3 position in compounds **9b**, **9f**, **9g**, and **9h** and cyclohexyl in **9b**, 3-bromobenzoyl in **9f**, 3-fluorobenzoyl in **9g**, and valeryl in **9h** attached to the carbonyl (C=O) group are essential for biological activity in RAMOS cells (Table 2). Compound **9b** showed weak activity in CCRF-CEM cells (IC_50_ = 35.53 ± 7.05 μM). Compounds **9g** and **9h** showed weak activity in CCRF-CEM cells (**9g**, IC_50_ = 46.46 ± 5.81 μM; **9h**, IC_50_ = 46.15 ± 3.13 μM). Compound **9j** showed moderate activity in U2OS cells (IC_50_ = 23.62 ± 6.44 μM), possibly due to non-specific activity.

We also assessed the cytotoxic effects of ibrutinib on our cell line panel. Compared to the derivatives we synthesized, ibrutinib exhibited significant cytotoxic activity against RAMOS cells (IC_50_ = 0.29 ± 0.04), in addition to its activity against BTK-null and ITK-positive cancer cells and non-malignant fibroblast lines (Table 2). This is not surprising given the broad range of kinases inhibited by ibrutinib. However, except for **9j**, none of our synthesized compounds that were effective against BTK-high RAMOS cells displayed any toxicity in both malignant and non-malignant cell lines lacking BTK. This data shows the selective cytotoxicity of our derivates on BTK-high cancer cells.

## In Vitro Pharmacological Properties

We next subjected **9b**, **9f**, and **9h** to in vitro ADME analyses. Results showed that the three selected are stable in the presence of plasma proteins and bind to proteins with >85% affinity (Table 3). However, **9b** is metabolized quickly by microsomes, suggesting a high probability that the compound will be primarily metabolized in the liver. Compared to **9b**, the intrinsic clearance of **9f** and **9h** was classified as medium in the microsomal stability assay. The passive diffusion mechanism for the three compounds was classified as low to medium by parallel artificial membrane permeability assay (PAMPA). All the three compounds showed poor permeability in assays with MDCK-MDR1 cells, indicating a low potential for penetration through the blood–brain barrier. Furthermore, **9b** showed low permeability in Caco-2 cells, suggesting that it is unsuitable for oral administration due to poor intestinal absorption. However, the medium permeability of **9f** and **9h** indicates a relatively good human intestinal permeability, suggesting that these compounds are suitable for oral administration.

**Table 3:** Pharmacological Properties of **9b**, **9f**, and **9h** Determined by In Vitro and In Vivo ADME Assays

	metabolism			permeability			
	in vitro			in vitro		in vivo	
				PAMPA		MDR1-MDCK	Caco-2
	plasma stability (category)	microsomal stability (clearance)	plasma protein binding (% bound)	log *P*	category	CNS (−ive/+ive)	category
**9b**	stable	high	88.60	–6.810	low	CNS –ive	low
						Papp (×10 × 10^–6^): 2.92	Papp (×10 × 10^–6^): 3.18
						efflux ratio: 9.3	efflux ratio: 2.69
						active efflux: yes	active efflux: yes
						% recovery: 60.23	% recovery: 45.37
**9f**	stable	medium	95.70	–7.414	low	CNS –ive	moderate
						Papp (×10 × 10^–6^): 3.2	Papp (×10 × 10^–6^): 10.59
						efflux ratio: 6.93	efflux ratio: 3.79
						active efflux: yes	active efflux: yes
						% recovery: 79.16	% recovery: 74.54
**9h**	stable	medium	96.66	–5.845	medium	CNS –ive	moderate
						Papp (×10 × 10^–6^): 5.43	Papp (×10 × 10^–6^): 7.18
						efflux ratio: 13.13	efflux ratio: 3.34
						active efflux: yes	active efflux: yes
						% recovery: 86.9	% recovery: 63.25

## Inhibition of BTK Signaling by Selected Compounds

**Figure 11:**
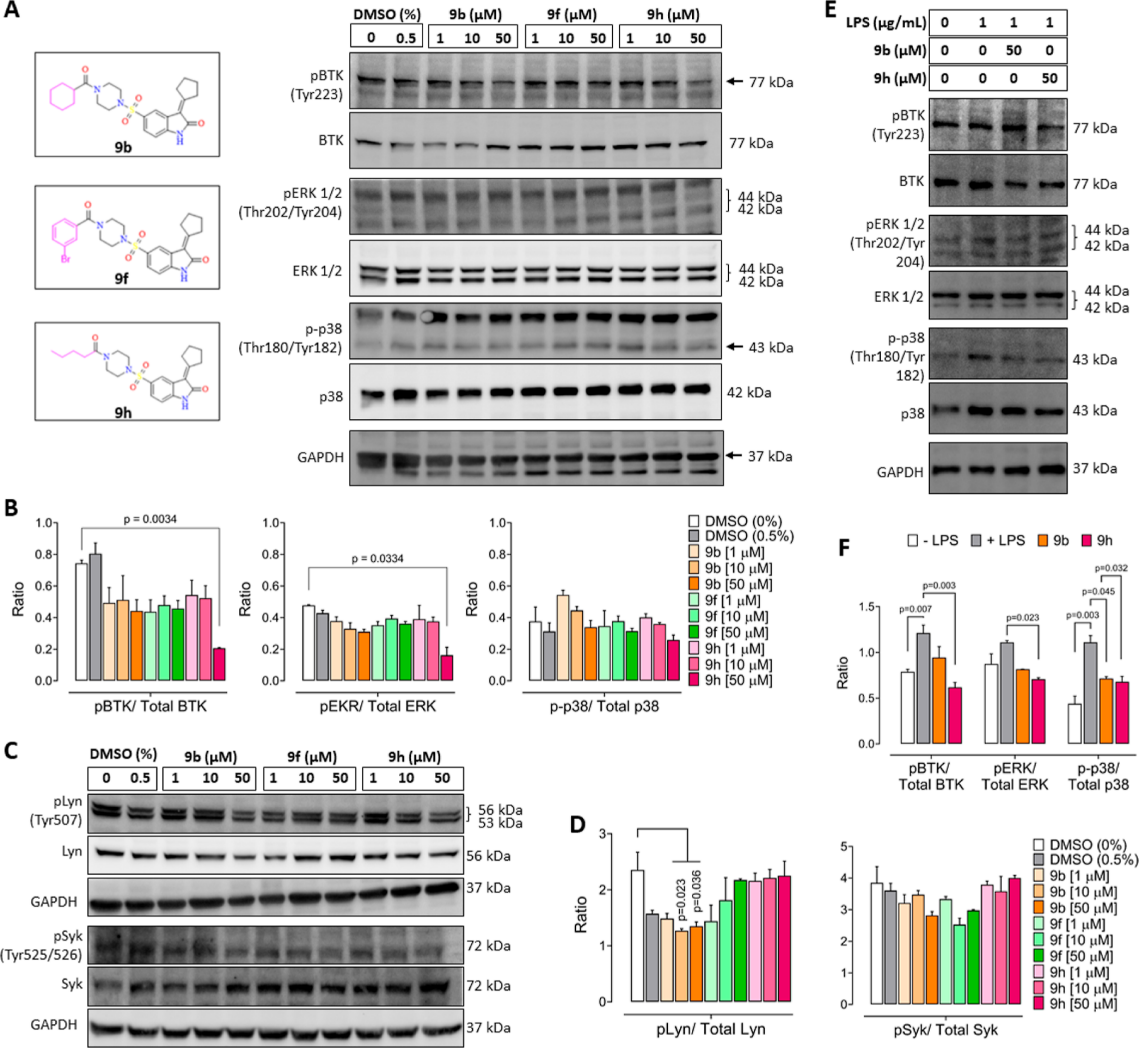
Compound effect on BTK signaling. (A) RAMOS cells were treated with **9b**, **9f**, and **9h** for 24 h at indicated concentrations, and whole protein extracts were probed for phosphorylated and total BTK, ERK, and p38 by western blotting. (B) Ratio of pBTK/total BTK, pERK/total ERK, and p-p38/total p38 band intensities. Mean ± SEM, *n* = 2–3, one-way ANOVA, Dunnett’s multiple comparison test. (C) Effect of **9b**, **9f**, and **9h** on Lyn and Syk phosphorylation in RAMOS cells after 24 h of treatment. (D) Ratio of pLyn/total Lyn and pSyk/total Syk bands shown in panel C. Mean ± SEM, *n* = 2, one-way ANOVA, Dunnett’s multiple comparison test. (E) RAMOS cells were stimulated with LPS for 10 min and then treated with **9b** and **9h** at 50 μM concentration for 3 h, and changes in BTK, ERK1/2, and p38 phosphorylation were probed by western blotting of the whole protein extract. (F) Ratio of pBTK/total BTK, pERK/total ERK, and p-p38/total p38 bands shown in panel E. Mean ± SEM, *n* = 2–3, one-way ANOVA, Dunnett’s multiple comparison test. Images of all uncropped blots are shown in the Supporting Information (Figure S17).

The effects of **9b**, **9f**, and **9h** at three concentrations were next examined on the activity of BTK tyrosine 223 phosphorylation [pBTK (Tyr223)]. Although all the three compounds decreased pBTK (Tyr223) levels, the decrease was significant following cell treatment with **9h** at 50 μM concentration (Figure 11A,B). We next checked the effect of these compounds on upstream proteins of the BCR signaling pathway, particularly Lyn and Syk, which are important regulators of BTK signaling.^1^ Lyn phosphorylation was significantly inhibited by only **9b**, whereas none of the compounds affected Syk (Figure 11C,D).

Based on these findings, we next selected **9b** and **9h** for further analysis in RAMOS cells stimulated with lipopolysaccharide (LPS), a well-known inducer of BTK phosphorylation, which subsequently activates downstream MAPK family proteins, including ERK1/2 and p38.^30,31^ Additionally, it is established that BTK inhibition blocks the activation of downstream MAPK family proteins.^31^ The western protein analysis showed that LPS stimulation significantly activated pBTK (Tyr223) signaling (Figure 11E,F). This pBTK (Tyr223) activation was significantly inhibited by only compound **9h**, as evidenced by the decrease in pBTK, pERK 1/2 (Thr202/Tyr204), and p-p38 (Thr180/Tyr182) levels (Figure 11F). The effect of **9b** was only evidenced on p-p38 (Thr180/Tyr182) levels, although there was no significant effect of **9b** on pBTK levels, suggesting that this compound might be non-specific in its activity. Overall, these findings, together with cytotoxicity data, underscore the potency of compound **9h** as a significant inhibitor of pBTK activity.

## Conclusions

As an initial effort, we screened the zinc database using oxindole as a core moiety to identify new BTK inhibitors. One compound with good binding energy with BTK protein and good ADME properties was chosen to design a focused library. Compound **819** was taken as the lead and further modified for better interactions with the BTK protein, and 10 analogous (**9a**–**9b**) were synthesized. The cytotoxic activity of the compounds was examined in a panel of cancer and non-cancer cell lines. Notably, four molecules, **9b**, **9f**, **9g**, and **9h**, exerted good anticancer activity in the micromole range in BTK-high RAMOS lymphoma cells. MD simulations for compounds **9f**, **9g**, and **9h** were conducted, and the RMSF values were below 2 Å. The RMSD and the ligand-RMSF percentage for Cα indicated the stability of compounds **9f**, **9g**, and **9h** with 5P9J, and the protein-bound conformations were confirmed by torsional analysis. Compound **9h** showed more protein–ligand contacts in the simulations. All the three compounds exhibited low permeability in assays conducted with MDCK-MDR1 cells, indicating limited potential for crossing the blood–brain barrier. Compound **9b** displayed low permeability in Caco-2 cells, suggesting that it may not be suitable for oral administration due to poor intestinal absorption. However, the medium permeability observed for **9f** and **9h** in Caco-2 cells suggests that these compounds have relatively good human intestinal permeability, indicating that they are suitable for oral administration. The antiproliferative activity of **9h** corresponded to its pBTK (Tyr223) inhibitory activity in RAMOS cells. Moreover, this compound did not affect Lyn and Syk, two proteins upstream of BTK in the BCR signaling pathway, suggesting that the anti-pBTK activity of **9h** is due to its activity on BTK. According to our present findings, **9h** exhibits promising specificity and efficacy in modulating BTK signaling pathways, warranting further investigation.

## Materials and Methods

### Selection of the Target Molecule

The crystal structure of BTK [Protein Data Bank (PDB) ID: 5P9J, resolution: 1.08 Å, *R*-value free: 0.224, and *R*-value work: 0.204, no mutations] was obtained from RCSB PDB.^32^ By removing its cocrystallized ligand and water molecules and adding missing residues, the protein was prepared using Swiss PDB Viewer. The SDF files from the ZINC15 database were used as they are for virtual screening. The ligand preparation for the designed molecules was drawn in ChemDraw and saved as SDF files. The schematic representation of the workflow is shown in Figure 3.

### Structure-Based Pharmacophore Selection

Oxindole was taken as the core moiety to search the molecules from the ZINC15 database, where around 550 molecules were selected and docked with the BTK protein (5P9J) using the PyRx Virtual screening tool.^33,34^ The top 19 molecules were selected, considering docking scores above 10 kcal/mol as criteria. From those 19 molecules, we have designed new molecules based on the pharmacophoric features, which play an essential role in the macromolecule ligand recognition and biological activity shown in Figure 4.

### Pharmacokinetics and Drug-Likeness Prediction

The compounds finalized after docking using the PyRx Virtual screening tool were further proceeded to predict their pharmacokinetics and drug-likeness. The physicochemical properties, pharmacokinetics, Log *P*, water solubility, and AMES toxicity were predicted by Swiss ADME. The drug-likeness properties were checked with Lipinski violations using Swiss ADME^35,36^ (Table 1).

### MD Simulation

The MD studies were conducted for the complex structures of the 5P9J protein with selected compounds **9f**, **9g**, and **9h** using Desmond Software Release 2018-4 for academic licensing (Schrödinger, LLC, New York, NY, USA) to check the stability of binding for all the complexes.^37^ The simulations used the 0.15 M NaCl and SPC water model to mimic a physiological ionic concentration. Energy minimization was conducted for 100 ps. The MD simulations were run for 20 ns at 300 K and standard pressure (1.01325 bar), with a dimension buffer of 10 Å × 10 Å × 10 Å with an orthorhombic box and an *NPT* ensemble. The energies were recorded at intervals of 1.2 ps. The MD-simulated net charge system of the protein–ligand complex was neutralized by adding Na^+^ or Cl^–^ ions. The Nosé–Hoover chain and Martyna–Tobias–Klein algorithms were used to maintain the temperature of all MD systems at 300 K and pressure at 1.01325 bar.

### Chemistry

All the solvents and reagents used for synthesis were purchased from commercial sources (Sigma-Aldrich, Avra, TCI). All reactions were observed by thin-layer chromatography using Merck classic aluminum silica plates with a thickness of 200 μm, size 20 × 20 cm, and were checked in Ultraviolet–visible spectroscopy at 254 nm. All compounds were purified using column chromatography with silica gel (60–100#) as the stationary phase. Proton ^1^H and ^13^C NMR spectra were recorded on an SA-AGILENT 400 MHz NMR and an (Ascend) AVANCE NEO 600 MHz FT-NMR spectrometer. Proton NMR chemical shifts are reported using tetramethylsilane (TMS) as a standard reference in parts per million (δ). ESI spectra were recorded on Micro mass, Quattro LC using ESI+ software with a capillary voltage of 3.98 kV and an ESI mode positive-ion trap detector. IR spectra were recorded on an FT-IR spectrometer (Shimadzu FT-IR 8300 spectrophotometer), and peaks were reported in cm^–1^. Melting points were measured in degrees centigrade (°C) using a MP apparatus and reported.

#### Synthesis of 2-Oxoindoline-5-sulfonyl Chloride **2**

To ice-cooled chlorosulfonic acid (20 mL) was added indolin-2-one **1** (5.0 g, 37.5 mmol) portionwise at 0 °C, stirred at RT for 30 min, and heated at 70 °C for 1 h. The reaction mixture was added slowly dropwise to the ice-cold water after cooling to RT and stirred for 30 min. The precipitated solid was washed with water (20 mL) thrice. Under reduced pressure, the resulting solid was dried to produce compound 2-oxoindoline-5-sulfonyl chloride **2**. (6.20 g, 71.4%) as light brown solid: ^1^H NMR (300 MHz, DMSO–*d*_6_): δ 10.55 (s, 1H), 7.49–7.46 (m, 2H), 6.79 (d, *J* = 7.8 Hz, 1H), 3.49 (s, 2H); MS (ESI + APCI): *m*/*z* = 231.9 [M + H]^+^.

#### Synthesis of *tert*-Butyl 4-((2-Oxoindolin-5-yl)sulfonyl)piperazine-1-carboxylate (**4**)

2-Oxoindoline-5-sulfonyl chloride **2** (3.00 g, 12.94 mmol) was taken in 1,4-dioxane (20 mL), *tert*-butyl piperazine-1-carboxylate **3** (3.60 g, 19.35 mmol) added, and charged with pyridine (1.80 g, 25.8 mmol) resultant reaction mixture stirred at RT for 2 h. Water (30 mL) was added to the reaction mixture to dilute it. The reaction mixture was then acidified to a pH of 6 with 2 N HCl solution and extracted with ethyl acetate (3 50 mL of EtOAc). The mixed organic layer was concentrated under reduced pressure, filtered, and dried over anhydrous Na_2_SO_4_. Hexane (2× 20 mL) washes were given and dried under reduced pressure to afford *tert*-butyl 4-((2-oxoindolin-5-yl)sulfonyl)piperazine-1-carboxylate (**4**) (3.80 g, 76.9%) as a light brown solid (crude directly used for next step without any purification); MS (ESI + APCI): *m*/*z* = 380.5 [M – H]^+^.

#### Synthesis of *tert*-Butyl 4-((3-Cyclopentylidene-2-oxoindolin-5-yl)sulfonyl)piperazine-1-carboxylate (**6**)

A solution of *tert*-butyl 4-((2-oxoindolin-5-yl)sulfonyl)piperazine-1-carboxylate 4 (2.8 g, 7.34 mmol) and cyclopentanone 5 (1.85 g, 22.04 mmol) in EtOH (20 mL) was charged with piperazine (1.87 g, 22.02 mmol); the resultant reaction mixture was stirred at RT for 1 h and then heated at 50 °C for 2 h. The reaction mixture was cooled to RT, and the solid was filtered and washed with hexanes (50 mL) to afford compound **6** (1.4 g, 42%) as an off-white solid; ^1^H NMR (400 MHz, DMSO-*d*_6_): 10.97 (s, 1H), 7.65–7.56 (m, 2H), 7.06 (d, *J* = 8.00 Hz, 1H), 3.41–3.38 (m, 4H), 3.02 (t, *J* = 5.60 Hz, 2H), 2.89 (t, *J* = 6.00 Hz, 2H), 2.80–2.80 (m, 4H), 1.85–1.75 (m, 4H), 1.34 (s, 9H); MS (ESI + APCI): *m*/*z* = 448 [M + H]^+^.

#### Synthesis of 3-Cyclopentylidene-5-(piperazin-1-ylsulfonyl)indolin-2-one·HCl (**7**)

A solution of compound **6** (1.4 g, 3.13 mmol), in CH_2_Cl_2_ (10 mL), was added to 4 M HCl in 1,4-dioxane (10 mL); the resulting reaction mixture was stirred at RT for 16 h. The reaction mixture was concentrated under reduced pressure, and the solid was washed with MTBE (20 mL) and dried to afford 3-cyclopentylidene-5-(piperazin-1-ylsulfonyl)indolin-2-one·HCl (**7**) (1.1 g, 92%) as an off-white solid; ^1^H NMR (400 MHz, DMSO–*d*_6_): δ 11.02 (s, 1H), 9.02 (s, 2H), 7.63–7.61 (m, 2H), 6.10 (d, *J* = 8.8 Hz, 1H), 3.16–3.13 (m, 8H), 3.03 (t, *J* = 5.8 Hz, 2H), 2.91 (t, *J* = 5.8 Hz, 2H), 1.89–1.74 (m, 4H); MS (ESI + APCI): *m*/*z* = 348 [M + H]^+^.

### General Procedure for the Synthesis of **9**

To a solution of compound 7 (1.0 equiv) in CH_2_Cl_2_ (3.0 mL) was added DIPEA (3.0 equiv), followed by RCOCl (1.2 equiv) at RT. The resultant reaction was stirred at rt for 3 h. The reaction mass was quenched with water and extracted with EtOAc (5 mL). The organic layer was dried over Na_2_SO_4_ and concentrated under reduced pressure to afford compound **9**. All series of compounds **9a**–**j** were synthesized using the same procedure.

#### 3-Cyclopentylidene-5-((4-isobutyrylpiperazin-1-yl)sulfonyl)indolin-2-one (**9a**)

Compound **9a** was obtained from 3-cyclopentylidene-5-(piperazin-1-ylsulfonyl)indolin-2-one hydrochloride (**7**) and isobutyryl chloride **8a** as an off white solid; yield 72.8%; mp 165–171 °C; IR (KBr): υ (cm^–1^): 1622_(–C=O for amide)_, 1702_(–C=O for amide)_, 3543_(−NH for secondary amine)_, 1325_(–SO_2_ bending for methylene)_, 1452_(–C=C– for aromatic)_; ^1^H NMR (400 MHz, DMSO–*d*_6_): δ 10.93 (s, 1H, –NH), 7.59–7.56 (m, 2H, Ar–H), 7.06–7.03 (d, *J* = 12 Hz, 1H, Ar–H), 3.58–3.53 (m, 4H, –CH_2_–CH_2_−), 3.04–2.99 (m, 2H, –NCH_2_–piperazine), 2.89–2.86 (m, 6H, –NCH_2_–piperazine), 2.77–2.75 (m, 1H, −CH–isobutyl), 1.87–1.75 (m, 4H, –CH_2_–CH_2_–cyclopentylidene), 0.91 (d, *J* = 6.4 Hz, 6H, –C(CH_3_)_2_–isobutyl); ^13^C NMR (600 MHz, CDCl_3_): δ 26.15, 26.94 (–CH_2_(C=C), 32.89, 35.44 (–CH_2_–CH_2_), 13.81, 22.69, 25.77, 27.19 (–CH_2_, Aliphatic), 40.83, 41.97, 45.98, 46.20 (–N(CH_2_)piperazine), 109.49, 118.61, 121.89, 124.87, 127.73, 127.90, 143.60 (Ar–C), 168.96 (−CH=CH), 170.50 (–N(C=O), 171.70 (–C=O(NH)); MS (ESI + APCI): *m*/*z* = 418.34 [M + H]^+^.

#### 5-((4-(Cyclohexanecarbonyl)piperazin-1-yl)sulfonyl)-3-cyclopentylideneindolin-2-one (**9b**)

Compound **9b** was obtained from 3-cyclopentylidene-5-(piperazin-1-ylsulfonyl)indolin-2-one hydrochloride (**7**) and cyclohexanoyl chloride **8b** as a light brown solid. mp 140–145 °C, Yield: 84.61%. FT-IR (KBr): ν cm^–1^: 1620_(–C=O for amide):_; 1710 cm^–1^_(–C=O for amide):_; 3423_(−NH for secondary amine)_; 1346_(–SO_2_ bending for methylene)_; 1462_(–C=C– for aromatic)_; ^1^H NMR (400 MHz, DMSO–*d*_6_): δ 10.95 (1s, 1H, –NH), 7.72–7.81 (d, 2H, Ar–H), 7.06 (s, 1H, Ar–H), 3.54–3.35 (m, 4H, piperazine), 3.01–2.87 (m, 6H), 1.87–1.65 (m, 7H, cyclopentylidene), 1.41–0.91 (m, 10H, cyclohexane); ^13^C NMR (600 MHz, CDCl_3_): δ 25.68, 26.18 (CH_2_(C=C), 35.47, 36.07 (CH_2_(CH_2_)), 20.70, 22.66, 29.06, 31.59, 34.66, 40.83 (CH_2_, cyclic), 42.71, 44.77, 46.04, 46.41 (N(CH_2_)), 109.43, 118.52, 121.95, 124.89, 127.72, 128.05, 143.36 (ArC),168.88 (CH=CH), 170.71 (N(C=O), 174.64 (–C=O(NH)); MS (ESI + APCI): *m*/*z* = 458.20 [M + H]^+^.

#### 3-Cyclopentylidene-5-((4-(cyclopropane carbonyl)piperazin-1-yl)sulfonyl)indolin-2-one (**9c**)

Compound **9c** was obtained from 3-cyclopentylidene-5-(piperazin-1-ylsulfonyl)indolin-2-one hydrochloride (**7**) and cyclopropanoyl chloride **8c** as a light brown solid. mp 136–142 °C, Yield: 90.30%; FT-IR (KBr): ν cm^–1^: 1637_(–C=O for amide)_; 1704_(–C=O for amide)_; 3408_(−NH for secondary amine)_; 1338_(–SO2__bending for methylene)_; 1453_(–C=C– for aromatic)_; ^1^H NMR (400 MHz, DMSO–*d*_6_): δ 10.94 (s, 1H, –NH), 7.60–7.56 (m, 2H, Ar–H), 7.05 (d, *J* = 8.0 Hz, 1H, Ar–H), 3.74–3.54 (m, 4H), 3.01–2.99 (m, 2H, piperazine), 2.91–2.88 (m, 6H), 1.89–1.75 (m, 5H, cyclopentylidene), 0.65–0.63 (m, 4H, cyclopropyl); ^13^CNMR (400 MHz, CDCl_3_): δ7.73 (CH_2_(CH_2_)), 10.88 (CH(C=O)), 25.78, 26.15 (CH_2_(C=C), 35.00, 35.44, ((CH_2_(CH_2_)), 45.71, 45.90, 45.94, 46.01 ((CH_2_(NH)), 109.29, 118.49, 121.98, 124.94, 127.75, 128.0, 143.36 (Ar–C), 168.55 (−CH=CH), 170.44 (–N(C=O), 172.05 (–C=O(NH)); MS(ESI + APCI): *m*/*z* = 416.17 [M + H]^+^

#### 5-((4-Benzoylpiperazin-1-yl)sulfonyl)-3-cyclopentylideneindolin-2-one (**9d**)

Compound **9d** was obtained from 3-cyclopentylidene-5-(piperazin-1-ylsulfonyl)indolin-2-one hydrochloride (**7**) and benzoyl chloride **8d** as a light brown solid. mp 135–140 °C, Yield: 80.07%; FT-IR (KBr): ν cm^–1^: 1617_(–C=O for amide)_; 1708_(–C=O for amide)_; 3418_(−NH for secondary amine)_; 1344_(–SO_2_ bending for methylene)_; 1455_(–C=C– for aromatic)_; ^1^H NMR (400 MHz, DMSO–*d*_6_): δ 10.94 (s,1H, –NH), 7.94 (d, *J* = 8.00 Hz, 1H, Ar–H), 7.60–7.33 (m, 6H, Ar–H) 7.05 (d, *J* = 8.0 Hz, 1H, Ar–H), 3.64 (m, 4H, cyclopentylidene), 3.01–2.66 (m, 8H, piperazine), 1.77–1.84 (m, 4H, cyclopentylidene); ^13^C NMR (600 MHz, CDCl_3_): δ 25.77, 26.14 (CH_2_(C=C), 35.09, 35.53 (CH_2_(CH_2_)), 46.01, 46.14, 46.18, 46.35 (–N(CH_2_)), 109.53, 118.52, 121.97, 127.15, 127.73, 128.14, 127.73, 130.13, 130.26, 133.58, 134.72, 143.33 (Ar–C), 169.09 (−CH=CH), 170.11 (–N(C=O), 171.06 (–C=O(NH)); MS (ESI + APCI): *m*/*z* = 452.16 [M + H]+.

#### 5-((4-(3-Chlorobenzoyl)piperazin-1-yl)sulfonyl)-3-cyclopentylideneindolin-2-one (**9e**)

Compound **9e** was obtained from 3-cyclopentylidene-5-(piperazin-1-ylsulfonyl)indolin-2-one hydrochloride (**7**) and 3 chlorobenzoyl chloride **8e** as a light brown solid. mp 133–138 °C, Yield: 87%; FT-IR (KBr): ν cm^–1^: 1615_(–C=O for amide)_; 1708_(–C=O for amide)_; 3445_(−NH for secondary amine)_; 1285_(–SO_2_ bending for methylene)_; 1442_(–C=C– for aromatic)_; ^1^H NMR (400 MHz, DMSO–*d*_6_): δ 10.94 (s, 1H, –NH), 7.60–7.50 (m, 2H, Ar–H), 7.49–7.48 (m, 1H, Ar–H), 7.44–7.43(m, 1H, Ar–H), 7.43–7.39 (m, 2H, Ar–H), 7.05 (d, *J* = 8.00 Hz, 1H, Ar–H), 3.70–3.66 (m, 2H, cyclopentylidene), 3.41–3.38 (d, *J* = 12.00 Hz, 2H, cyclopentylidene), 3.03–2.87 (m, 8H, piperazine), 1.86–1.75 (m, 4H, cyclopentylidene)); MS (ESI + APCI): *m*/*z* = 486.28 [M + H]^+^.

#### 5-((4-(3-Bromobenzoyl)piperazin-1-yl)sulfonyl)-3-cyclopentylideneindolin-2-one (**9f**)

Compound **9f** was obtained from 3-cyclopentylidene-5-(piperazin-1-ylsulfonyl)indolin-2-one hydrochloride (**7**) and 3 bromobenzoyl chloride **8f** as a light brown solid. mp 130–135 °C, Yield: 76.51%; FT-IR (KBr): ν cm^–1^: 1615_(–C=O for amide)_; 1701_(–C=O for amide)_; 3447_(−NH for secondary amine)_; 1309_(–SO2__bending for methylene)_; 1435_(–C=C– for aromatic)_; ^1^H NMR (400 MHz, DMSO–*d*_6_): δ 10.9 (s, 1H, –NH), 7.84–7.82 (m, 1H, Ar–H), 7.64–7.55 (m, 2H, Ar–H), 7.48 (d, *J* = 8.00 Hz, 1H, Ar–H), 7.38–7.33 (m, 2H, Ar–H), 7.06 (d, *J* = 12.00 Hz, 1H, Ar–H), 3.64 (d, *J* = 32.00 Hz, 4H, cyclopentylidene), 3.03–2.87 (m, 8H, piperazine), 1.86–1.75 (m, 4H, cyclopentylidene); ^13^C NMR (600 MHz, CDCl_3_): δ 25.76, 26.13 (CH_2_(C=C), 35.15, 35.58 (CH_2_(CH_2_)), 45.91 (–N(CH_2_)), 109.75, 118.56, 121.93, 122.81, 124.89, 125.60, 127.75, 128.65, 130.24, 131.72, 133.31, 136.65, 143.35 (Ar–C), 168.76 (−CH=CH), 169.58 (N(C=O), 71.56 (–C=O(NH)); MS (ESI + APCI): *m*/*z* = 532.22 [M + H]^+^.

#### 3-Cyclopentylidene-5-((4-(3-fluorobenzoyl)piperazin-1-yl)sulfonyl)indolin-2-one (**9g**)

Compound **9g** was obtained from 3-cyclopentylidene-5-(piperazin-1-ylsulfonyl)indolin-2-one hydrochloride (**7**) and 3 fluorobenzoyl chloride **8g** as a cream color solid. mp 132–135 °C, Yield: 78%; FT-IR (KBr): ν cm^–1^; 1624_(–C=O for amide)_; 1704_(–C=O for amide)_; 3448_(−NH for secondary amine)_; 1335 _(–SO2__bending for methylene)_: ν cm^–1^; 1449_(–C=C– for aromatic)_; ^1^H NMR (400 MHz, DMSO–*d*_6_): δ 10.97 (s, 1H, –NH), 7.60–7.56 (d, *J* = 16.00 Hz, 2H, Ar–H), 7.46–7.44 (d, *J* = 8.0 Hz, 1H, Ar–H), 7.27–7.16 (m, 3H, Ar–H), 7.07–7.05 (d, *J* = 8 Hz,1H, Ar–H), 3.99–3.03 (m, 4H), 3.69 (m, 3H), 3.37 (s, 1H, piperazine), 2.91–2.80 (m, 4H), 1.86–1.75 (m, 4H, cyclopentylidene); ^13^C NMR (400 MHz, CDCl_3_): δ 25.76, 26.17 (CH_2_(C=C), 35.05, 35.48 (CH_2_(CH_2_)), 46.05 (N(CH_2_)), 109.57, 114.33, 114.56, 117.19, 118.59, 121.91, 122.77, 124.95, 127.95, 130.57, 136.81, 143.59, 161.28 (Ar–C), 163.75 (−CH=CH), 168.97 (–N(C=O), 170.77 (–C=O(NH)); MS (ESI + APCI): *m*/*z* = 470.12 [M + H]^+^.

#### 3-Cyclopentylidene-5-((4-pentanoyl piperazin-1-yl)sulfonyl)indolin-2-one (**9h**)

Compound **9h** was obtained from 3-cyclopentylidene-5-(piperazin-1-ylsulfonyl)indolin-2-one hydrochloride (**7**) and valeroyl chloride (**8h**) as an off-white solid. mp 130–135 °C, Yield: 76.51%; FT-IR (KBr): ν cm^–1^; 1632_(–C=O for amide)_; 1701_(–C=O for amide)_; 3405_(−NH for secondary amine)_; 1326_(–SO_2_ bending for methylene)_; 1452_(–C=C– for aromatic)_; ^1^H NMR (400 MHz, DMSO–*d*_6_): δ 10.94 (s, 1H, –NH), 7.59–7.55 (m, 2H, Ar–H), 7.05 (d, *J* = 12.00 Hz, 1H, phenyl), 3.51 (s, 4H), 3.02 (d, *J* = 8.00 Hz, 2H), 2.99–2.83 (m, 6H), 2.24–2.20 (m, 3H), 1.87–1.83 (m, 4H), 1.78–1.75 (m, 3H), 1.39–1.33 (m, 6H), 1.25–1.20 (m, 3H, aliphatic, valeroyl); ^13^C NMR (600 MHz, CDCl_3_): δ 26.15, 26.94 (CH_2_(C=C), 32.89, 35.44 (CH_2_(CH_2_)), 13.81, 22.69, 25.77, 27.19 (CH_2_, aliphatic), 40.83, 41.97, 45.98, 46.20 (–N(CH_2_)), 109.49, 118.61, 121.89, 124.87, 127.73, 127.90, 143.60 (Ar–C), 168.96 (−CH=CH), 170.50 (–N(C=O), 171.70 (–C=O(NH)); MS (ESI + APCI): *m*/*z* = 432.14 [M + H]^+^.

#### 3-Cyclopentylidene-5-((4-nicotinoylpiperazin-1-yl)sulfonyl)indolin-2-one (**9i**)

Compound **9i** was obtained from 3-cyclopentylidene-5-(piperazin-1-ylsulfonyl)indolin-2-one hydrochloride (**7**) and 3-pyridine chloride **8i** as an off-white solid. mp 195–200 °C, Yield: 62%; FT-IR (KBr): ν cm^–1^; 1610_(–C=O for amide)_; 1704_(–C=O for amide)_; 3414_(−NH for secondary amine)_; 1332_(–SO_2_ bending for methylene)_; 1464_(–C=C– for aromatic)_; ^1^H NMR (400 MHz, DMSO–*d*_6_): δ 10.94 (s, 1H, –NH), 8.62 (d, *J* = 4.00 Hz, 1H, Ar–H), 8.52 (d, *J* = 8.00 Hz, 1H), 7.78 (d, *J* = 12.00 Hz, 1H, Ar–H), 7.60–7.56 (m, 2H), 7.44–7.41 (m, 1H), 7.06 (d, *J* = 12.00 Hz, 1H, Ar–H), 3.71–0.00 (m, 2H), 3.41–3.37 (m, 2H, cyclopentylidene), 3.03–2.87 (m, 8H, piperazine), 1.86–1.75 (m, 4H, cyclopentylidene); ^13^C NMR (600 MHz, CDCl_3_): δ 25.17, 26.13 (CH_2_(C=C), 35.04, 35.48 (CH_2_(CH_2_)), 45.89, 46.02, 46.13, 46.68 (–N(CH_2_)), 109.29, 121.98, 123.66, 125.08, 127.72, 127.88, 130.69, 135.25, 143.35, 147.90, 151.26 (Ar–C), 167.81 (−CH=CH), 168.24 (–N(C=O), 170.68 (–C=O(NH)): MS (ESI + APCI): *m*/*z* = 453.14 [M + H]^+^.

#### 3-Cyclopentylidene-5-((4-(3-(trifluoromethyl)benzoyl)piperazin-1-yl)sulfonyl)indolin-2-one (**9j**)

Compound **9j** was obtained from 3-cyclopentylidene-5-(piperazin-1-ylsulfonyl)indolin-2-one hydrochloride (**7**) and 3-trifluoromethyl benzoyl chloride **8j** as a light cream solid. mp 139–143 °C, Yield: 83%; FT-IR (KBr): ν cm^–1^; 1618_(–C=O for amide)_; 1707_(–C=O for amide)_; 3445_(−NH for secondary amine)_: cm^–1^; 1332_(–SO_2_ bending for methylene)_; 1469_(–C=C– for aromatic)_; ^1^H NMR (400 MHz, DMSO–*d*_6_): δ 10.94 (s, 1H, –NH), 7.79–7.56 (m, 7H, Ar–H), 3.40 (d, *J* = 8.00 Hz, 2H), 3.72 (d, *J* = 8.00 Hz, 2H, cyclopentylidene), 3.03–2.87 (m, 8H, piperazine), 1.85–1.7 (m, 4H, cyclopentylidene); ^13^C NMR (400 MHz, CDCl_3_): δ 25.76, 26.13 (CH_2_(C=C), 35.12, 35.55 (CH_2_(CH_2_)), 46.06 (N(CH_2_)), 109.71, 118.58, 121.91, 127.02, 128.15, 129.28, 129.78, 130.40, 131.03, 135.54, 143.47 (Ar–C), 168.87 (−CH=CH), 169.45 (–N(C=O), 171.35 (–C=O(NH)); MS (ESI + APCI): *m*/*z* = 520.14 [M + H]^+^.

### Cell Lines

All cell lines were purchased from ATCC (Middlesex, UK) or DSMZ (Braunschweig, Germany) and maintained according to recommendations at 37 °C in a humidified incubator (5% CO_2_/atmospheric air). Multidrug-resistant sublines (CEM-DNR and K562-TAX) expressing the LRP and P-glycoprotein transporter proteins were derived and cultured as previously described.^38^ Cell lines were routinely tested for mycoplasma contamination and authenticated biweekly or monthly.

### Cytotoxicity Assay

The cytotoxic activity of all the 10 compounds was tested under in vitro conditions using a 3 day standard 3-(4,5-dimethylthiazol-2yl)-5-(3-carboxymethoxyphenyl)-2-(4-sulfophenyl)-2*H*-tetrazolium reduction assay in 384-well plates on a robotic high-throughput screening platform (HighResBio, Boston, MA) as described elsewhere.^38^ The IC_50_ values were calculated from the respective dose–response curves of compounds with Dotmatics (San Diego, CA, USA).

### In Vitro Pharmacology

Selected compounds were assayed for human plasma and liver microsomal stability in vitro, PAMPA and cellular permeability models of gastrointestinal resorption, and the blood–brain barrier using Caco-2 and MDR1-MCDK cells as previously described.^38^ Samples were analyzed in an Agilent RapidFire 300 High-Throughput Mass Spectrometry System (RF-MS; Agilent, Wakefield, MA) with subsequent detection in a Qtrap 5500 mass spectrometer (AB Sciex, Concord, Canada).

### Western Blotting

RAMOS cells were plated at 0.5 × 10^6^/mL density in six-well plates. After 24 h, cells were treated with compounds at concentrations of 1, 10, and 50 μM for 24 h. The concentration of the vehicle was 0.5% in the highest test drug concentration (50 μM). LPS stimulation of RAMOS cells followed by drug treatment was performed as described previously.^19^

To obtain whole-cell protein extracts, cells were collected, washed with 1× Tris-buffered saline, and lysed in RIPA buffer [Thermo Fisher Scientific, Massachusetts, USA), Cat #89901] supplemented with protease (Roche, Basel, Switzerland, Cat. # 04693116001) and phosphatase (Roche, Basel, Switzerland; Cat. #04906837001) inhibitors by sonication using a Cup Horn sonicator (Qsonica, LLC., Connecticut, USA). The cell lysate was then centrifuged at 12,000 rpm for 30 min at 4 °C, and the supernatant was collected. 35 μg of protein samples was processed for electrophoresis and western blotting, as described elsewhere.^38^

Primary antibodies used were purchased from Cell Signaling Technology, Inc., Massachusetts, USA, and included BTK (1:1000; Cat. # 3533S), phosphor-BTK (Tyr223) (1:1000; Cat # 5082S), p44/42 MAPK (ERK1/2) (1:1000; Cat # 9102S), phospho-p44/42 MAPK (ERK1/2) (Thr202/Tyr204) (1:1000; Cat # 4376S), p38 MAPK (1:1000; Cat # 9212S), phospho-p38 MAPK (Thr180/Tyr182) (1:1000; Cat # 9211S), Lyn (C13F9) (1:1000: Cat # 2796), phospho-Lyn (Tyr507) (1:1000; Cat #2731), Syk (1:1000; Cat # 2712), phospho-Syk (Tyr525/526) (C87C1) (1:1000; Cat # 2710), and GAPDH (1:4000; Cat. # 2118). Primary antibody-stained blots were developed using antimouse (Cat. # A11034) or antirabbit (Cat. # A21202) Alexa Fluor 488-conjugated secondary antibodies (Invitrogen, Massachusetts, USA) at 1:2000 dilution for 1–2 h at RT in the dark. The blots were then imaged using a Gel Doc XR + Gel Documentation System (Bio-Rad, California, USA) with appropriate filters for Alexa Fluor 488 to visualize protein bands. Band intensities were quantified using NIH ImageJ Software (NIH, Bethesda, Maryland, USA).

### Statistical Analysis

All blots were analyzed using NIH ImageJ software (Bethesda, Maryland, USA). All statistical analyses were performed in Statistica Version 14 (TIBCO Software Inc., CA, USA) or GraphPad Prism 10 (GraphPad Software, Boston, MA, USA), and differences were considered significant at *P* < 0.05.

## Data Availability

All data generated or analyzed during this study are included in this published article and its Supporting Information. Data supporting the biological part of the study is stored in the online repository of IMTM. This article does not contain any studies with animals performed by any of the authors.

## Supporting Information Available

Characterization of all compounds (^1^H NMR, ^13^C NMR, HRMS, HPLC, and FT-IR Spectra); MD simulation data of protein (5P9J) and ligands (compounds **9f** and **9g**); representative dose–response curves of compounds **9b**, **9f**, **9g**, **9h**, and **9j**; unedited images of the western blots; and list of the contents of the materials supplied (PDF)
