# Supplementary material for: Novel 5-Substituted Oxindole Derivatives as Bruton’s Tyrosine Kinase Inhibitors: Design, Synthesis, Docking, Molecular Dynamics Simulation, and Biological Evaluation
Source: ACS Omega. 2024 Feb 7;9(7):8067–81. doi: 10.1021/acsomega.3c08343 (PMC10882696; doi:10.1021/acsomega.3c08343)
Supplement: Supplementary file 1 — ao3c08343_si_001.pdf [file ao3c08343_si_001.pdf]

Supplementary Material:

Novel 5-Substituted Oxindole Derivatives as Bruton's Tyrosine Kinase Inhibitors: Design, Synthesis, Docking, Molecular Dynamic Simulation, and Biological Evaluation

Vani Madhuri Velavalapalli,[a,b] Venkatanarayana Chowdary Maddipati,[b] Soňa Gurská,[c,d] Narendran Annadurai,[c] Barbora Lišková,[c] Naresh Kumar Katari,[b] Petr Džubák,[c,d] Marián Hajdúch,[c,d] Viswanath Das,[*c,d] Rambabu Gundla,[*b]

[a] GITAM School of Pharmacy, GITAM Deemed to be University, Hyderabad, Telangana-502329, India

[b] Department of Chemistry, GITAM School of Science, GITAM Deemed to be University, Hyderabad, Telangana-502329, India

[c] Institute of Molecular and Translational Medicine, Faculty of Medicine and Dentistry, Palacký University and University Hospital Olomouc, Hněvotínská 1333/5, 77900 Olomouc, Czech Republic

[d] Czech Advanced Technologies and Research Institute (CATRIN), Institute of Molecular and Translational Medicine, Palacký University Olomouc, Olomouc, Czech Republic

* *Corresponding authors*

Viswanath Das (Email: viswanath.das@upol.cz, Tel.: +420 585 632 111)

Rambabu Gundla (Email: rgundla@gitam.edu, Tel.: +91-9849869933)

1. CHARACTERIZATION OF ALL COMPOUNDS - ^{1}H NMR, ^{13}C NMR, AND FT-IR SPECTRA

^{1}H NMR of Compound-2

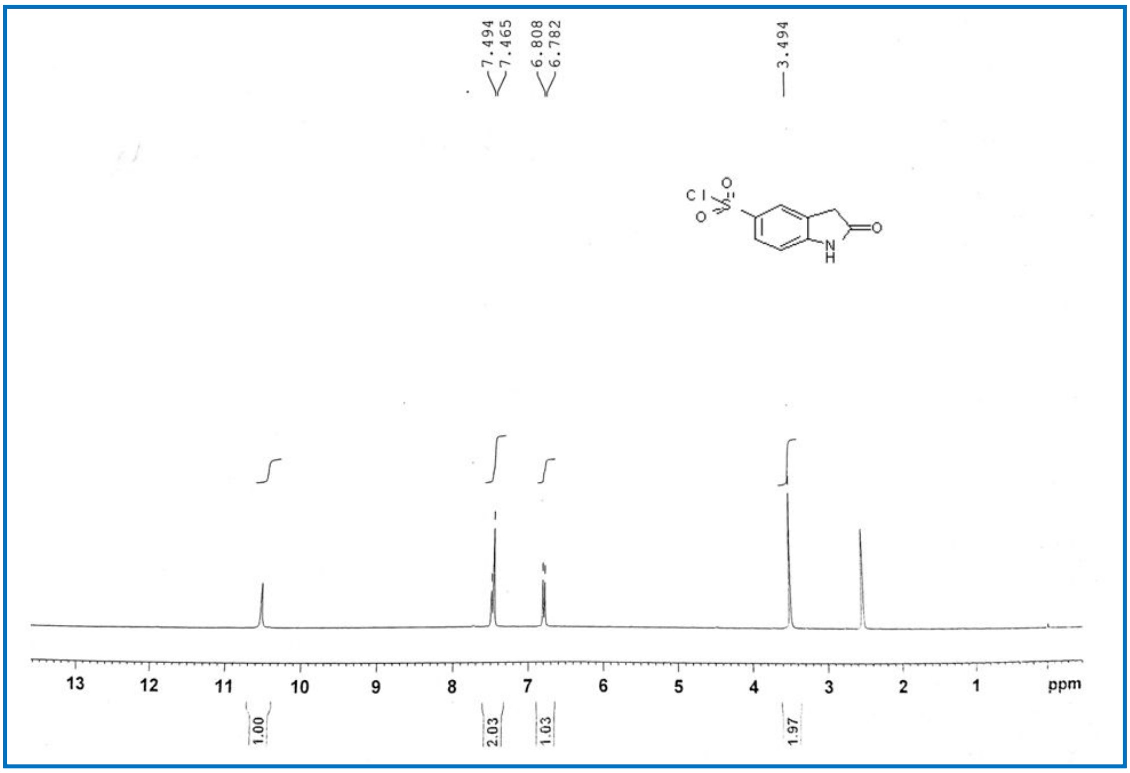

^{1}H NMR of Compound-6

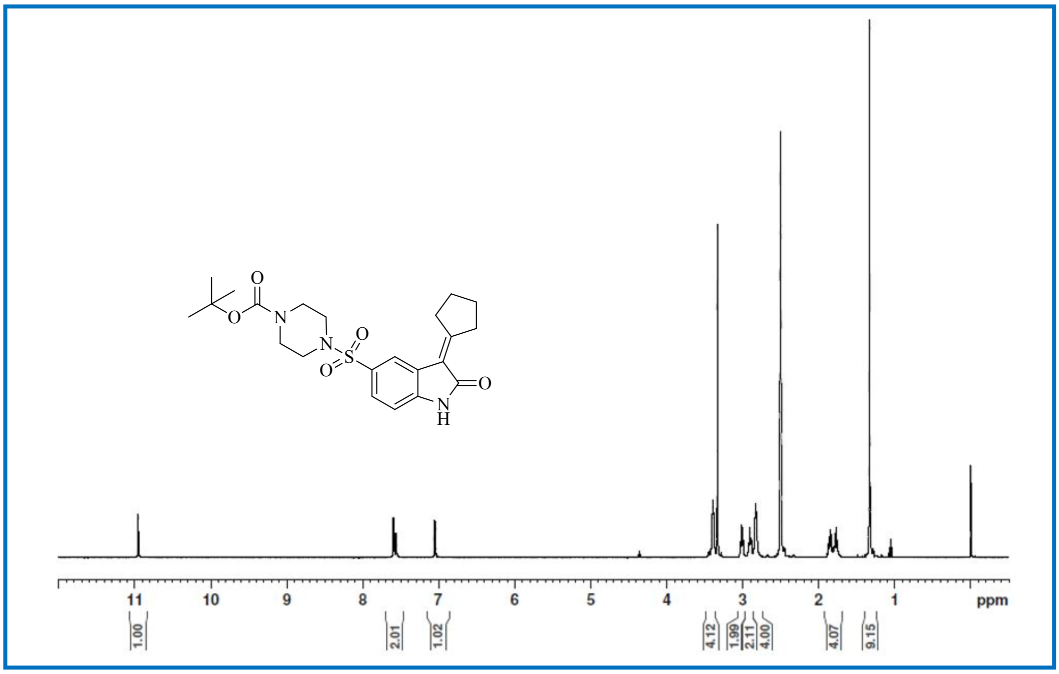

Mass of Compound-6

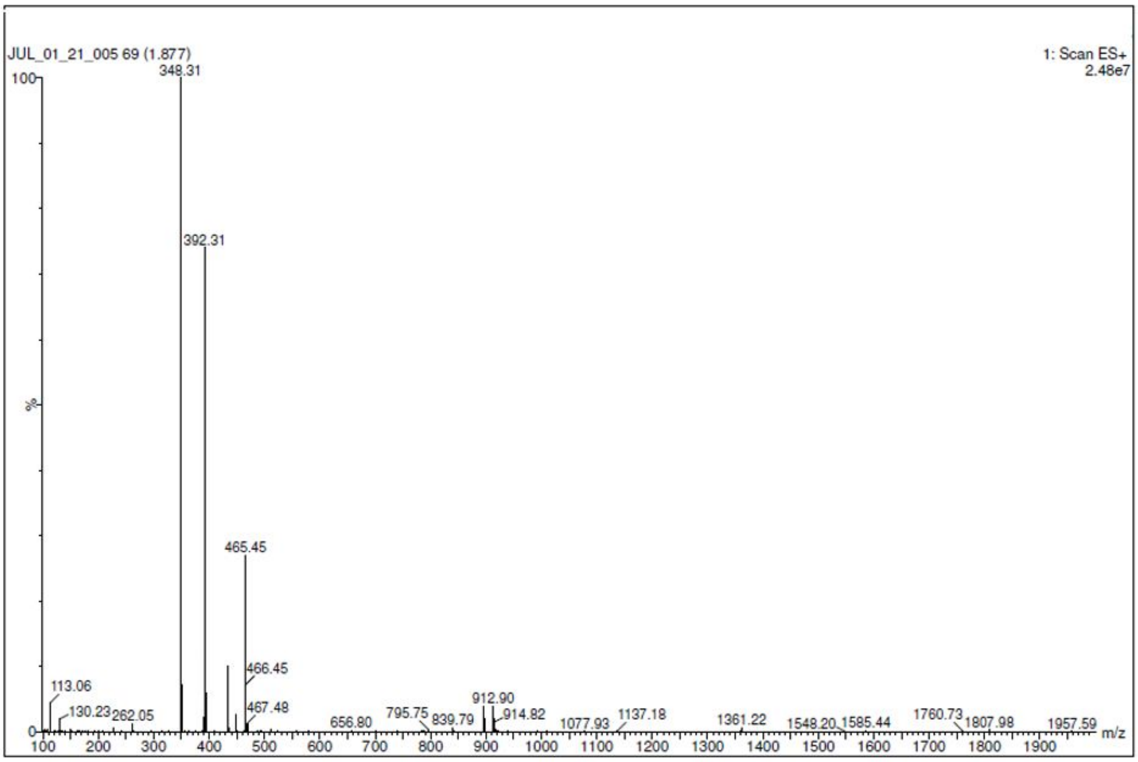

[1] H NMR of Compound-7

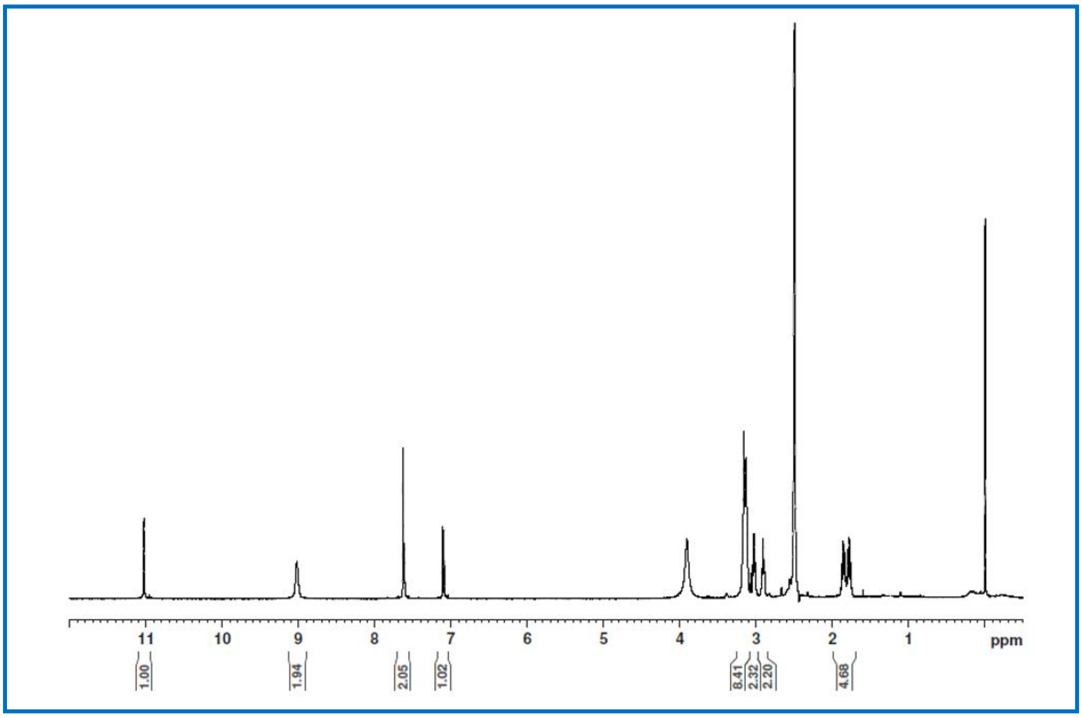

Mass of Compound-7

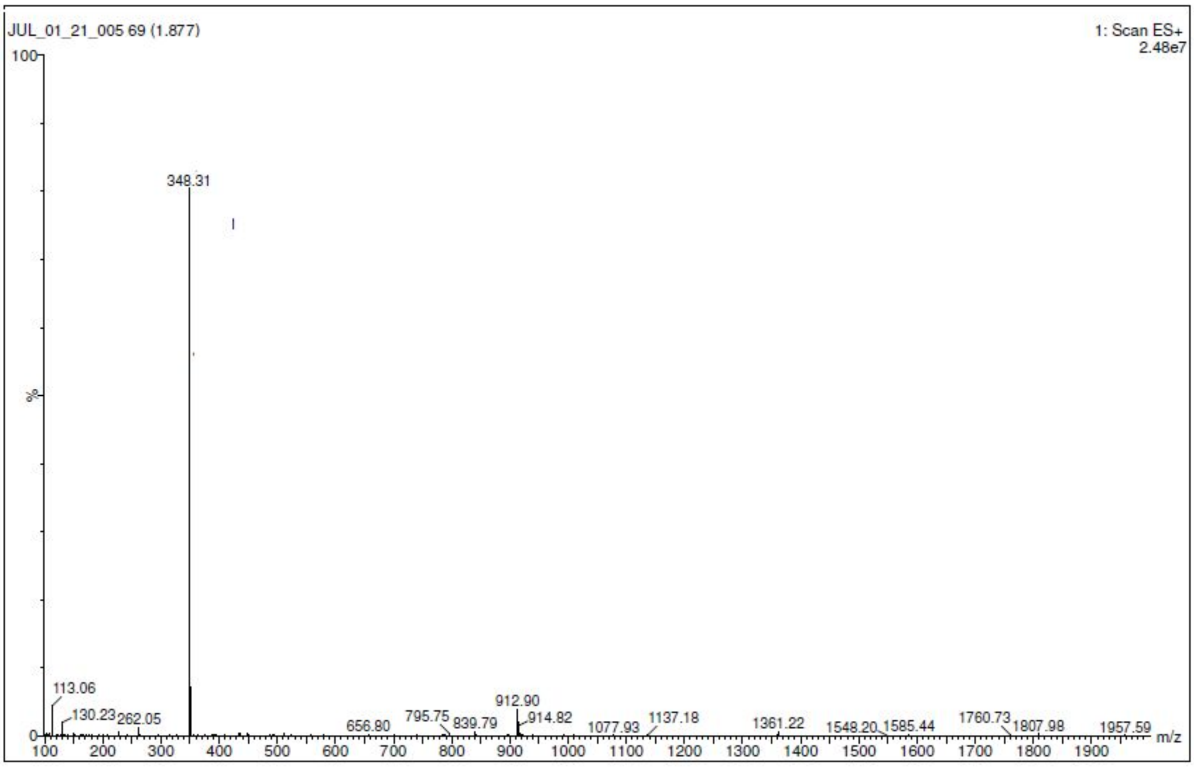

[1] H NMR of Compound-9a

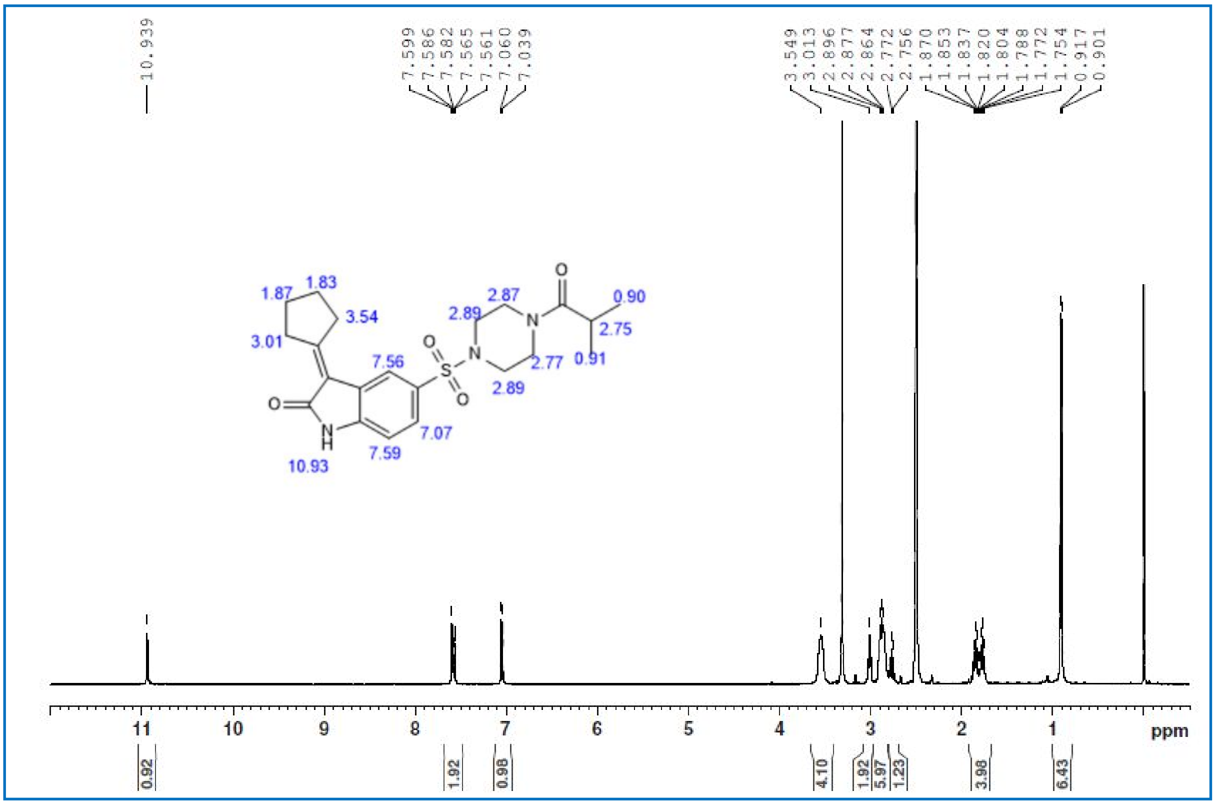

IR Compound-9a

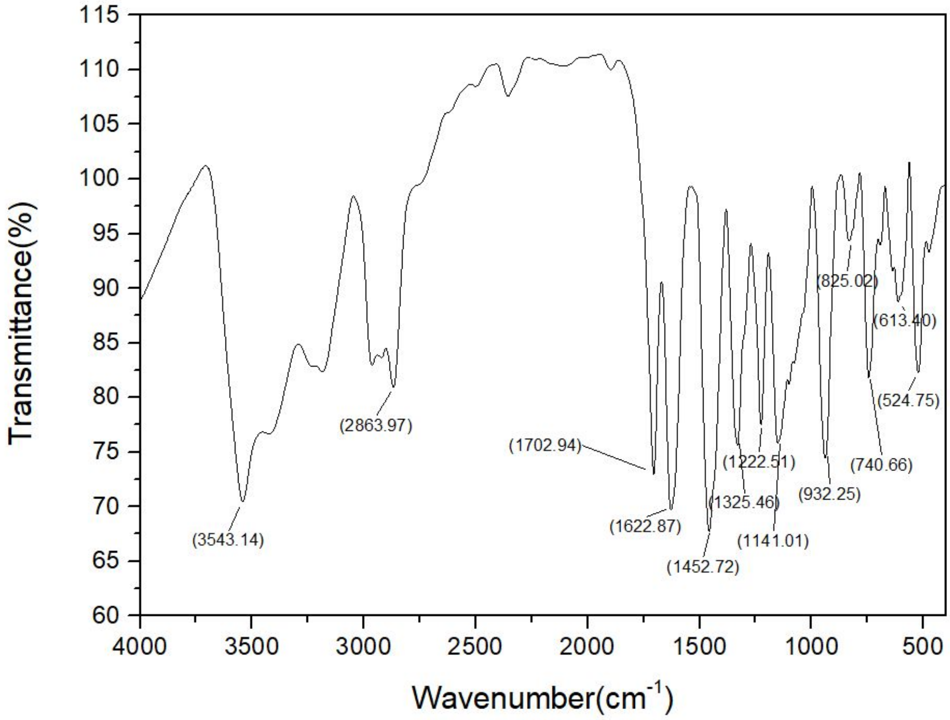

Mass of Compound-9a

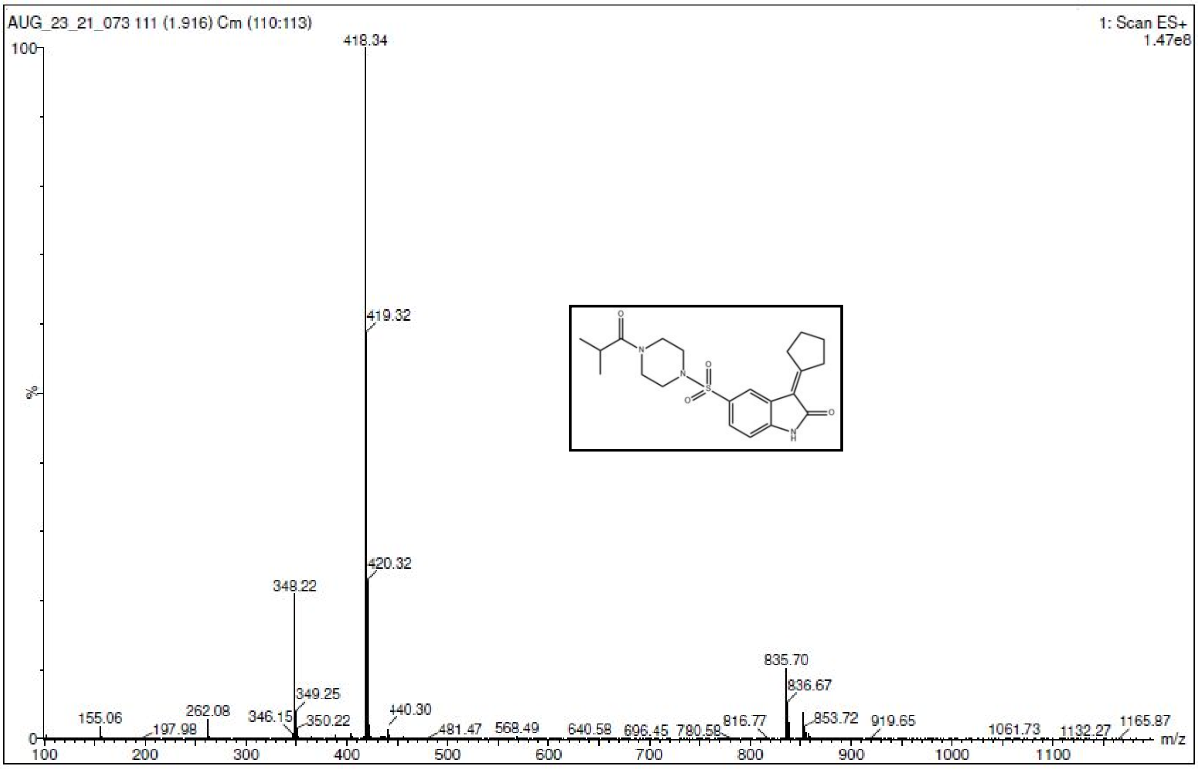

^{13}C-NMR of Compound-9a

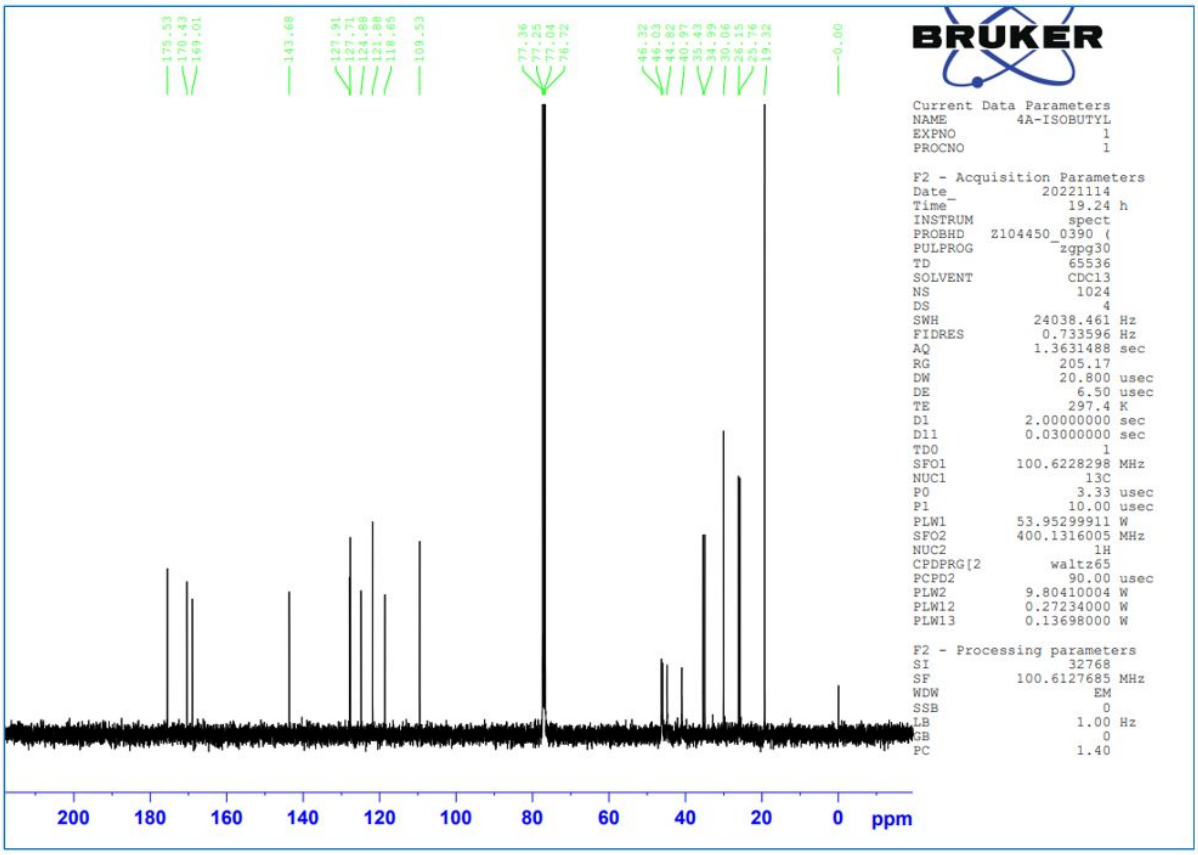

[1] H NMR of Compound-9b

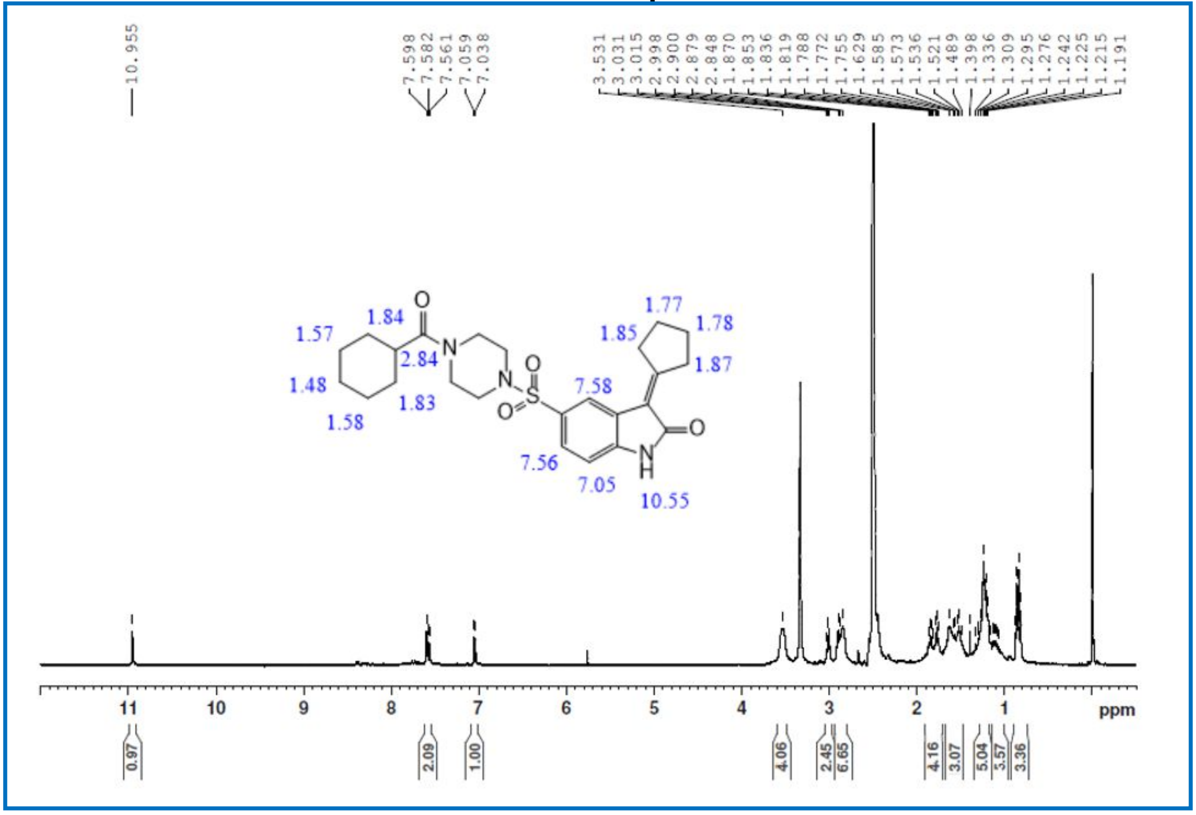

Mass of Compound-9b

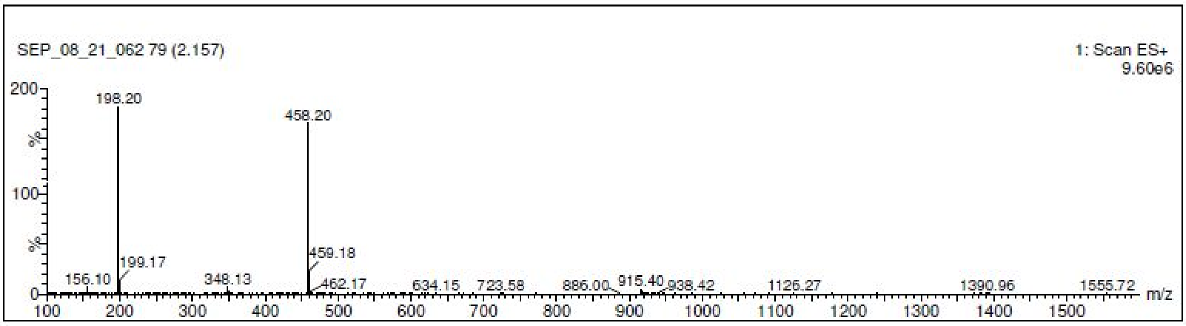

IR of Compound-9b

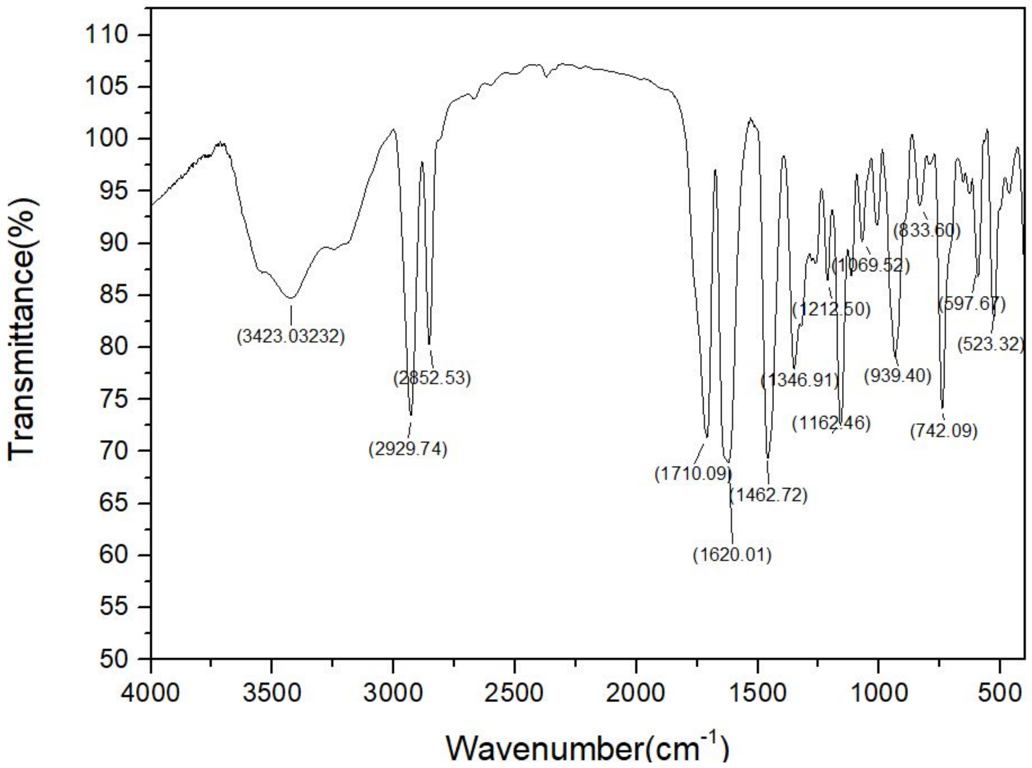

^{13}C -NMR of Compound-9b

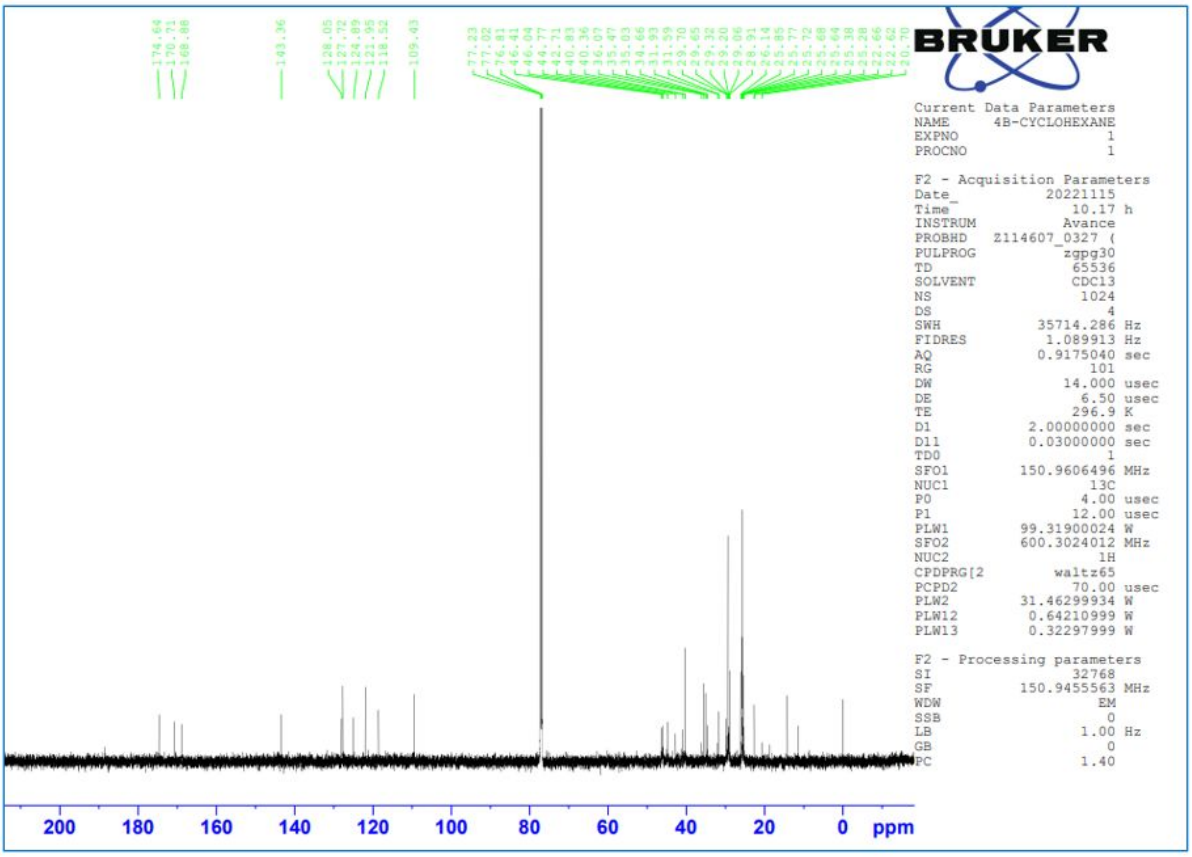

[1] H NMR of Compound-9c

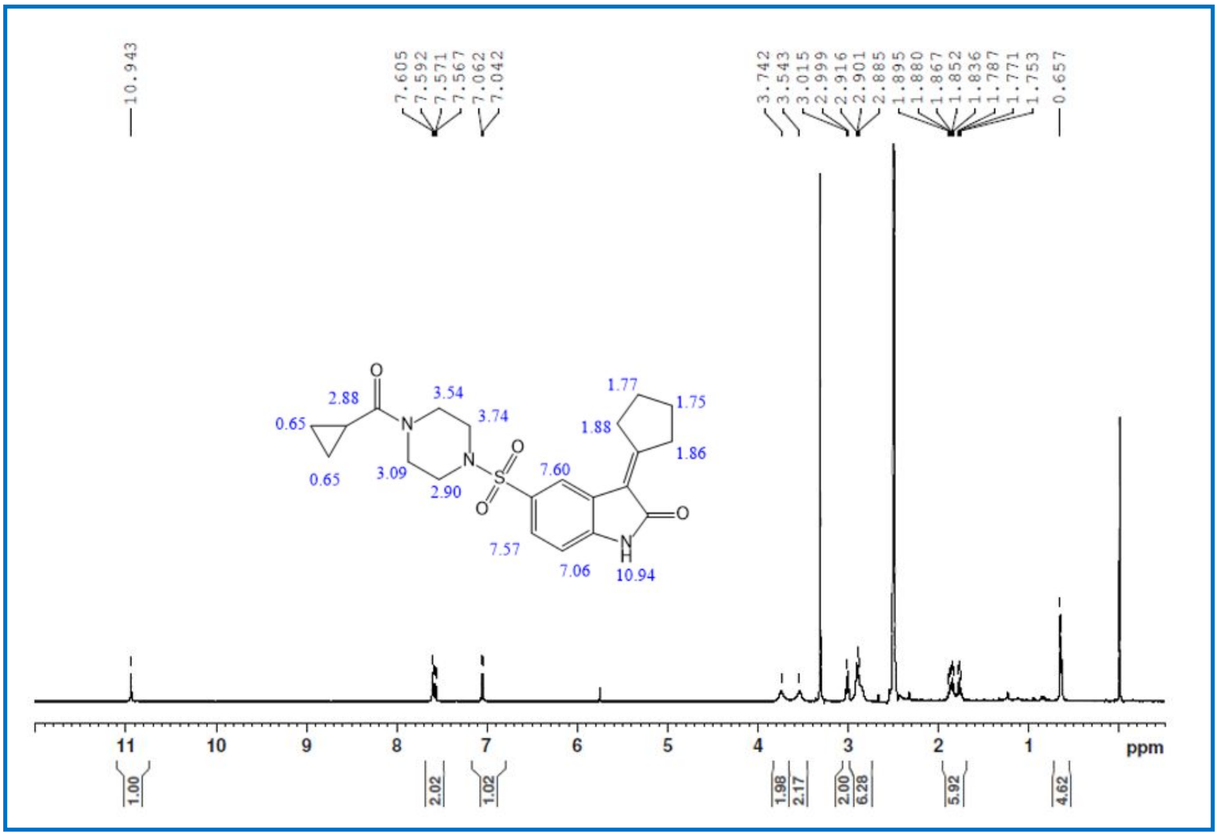

Mass of compound-9c

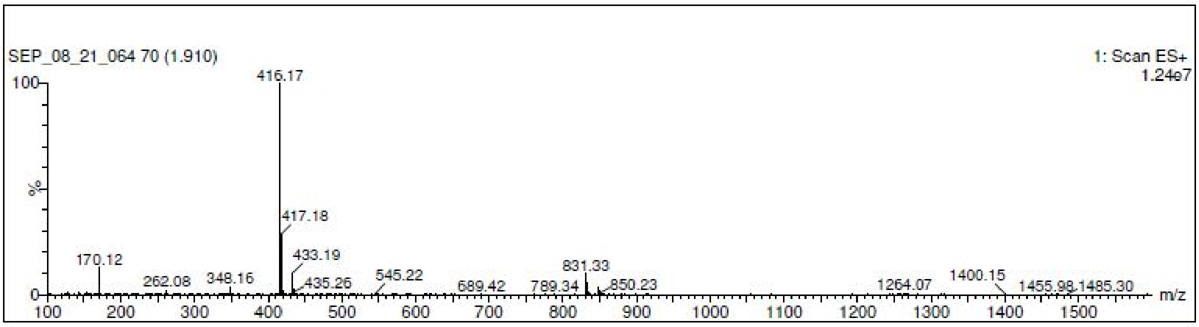

IR of Compound-9c

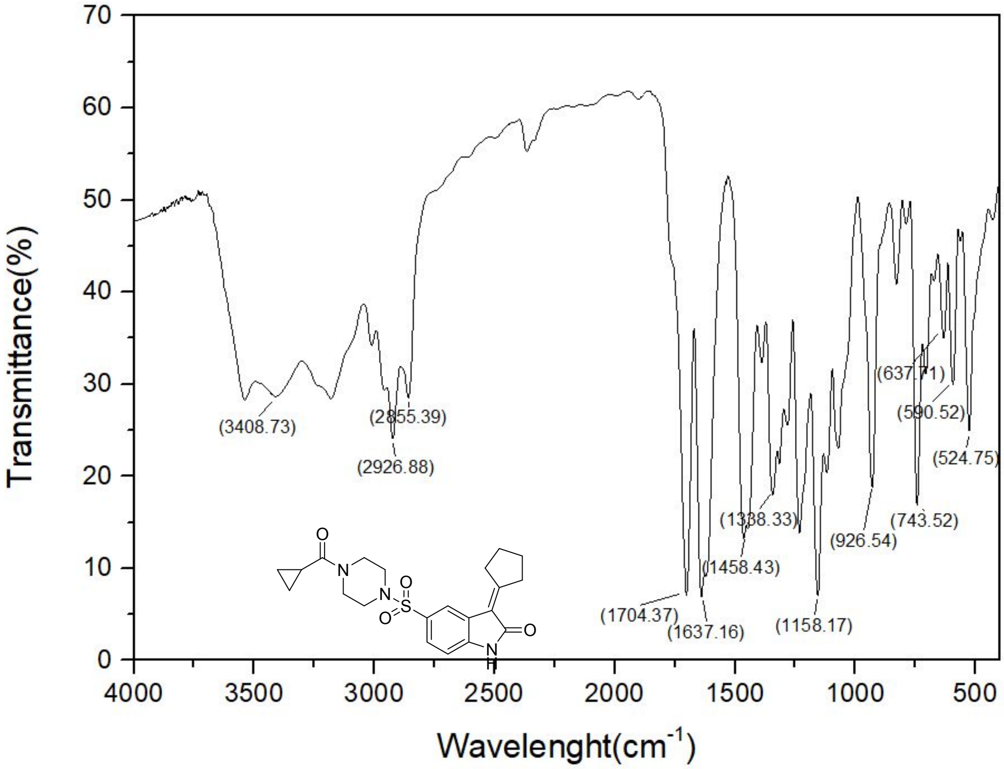

^{13}C -NMR of Compound-9c

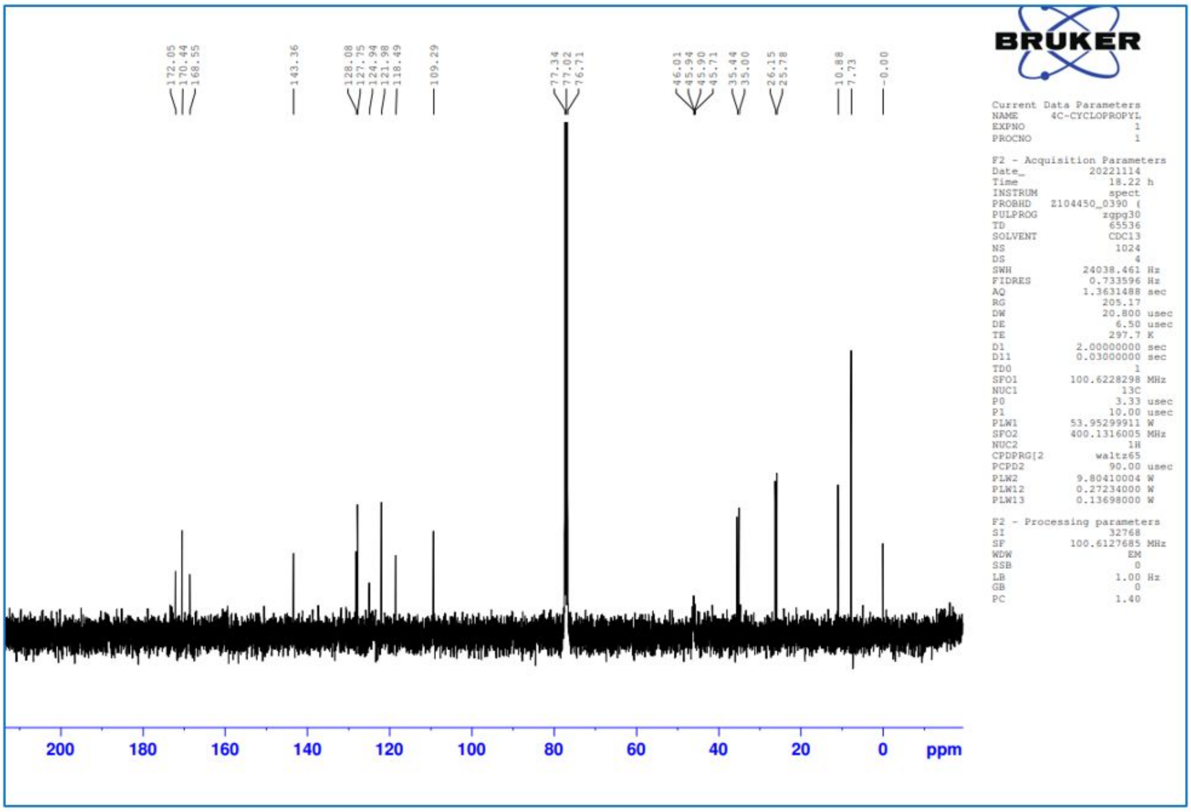

[1] H NMR of Compound-9d

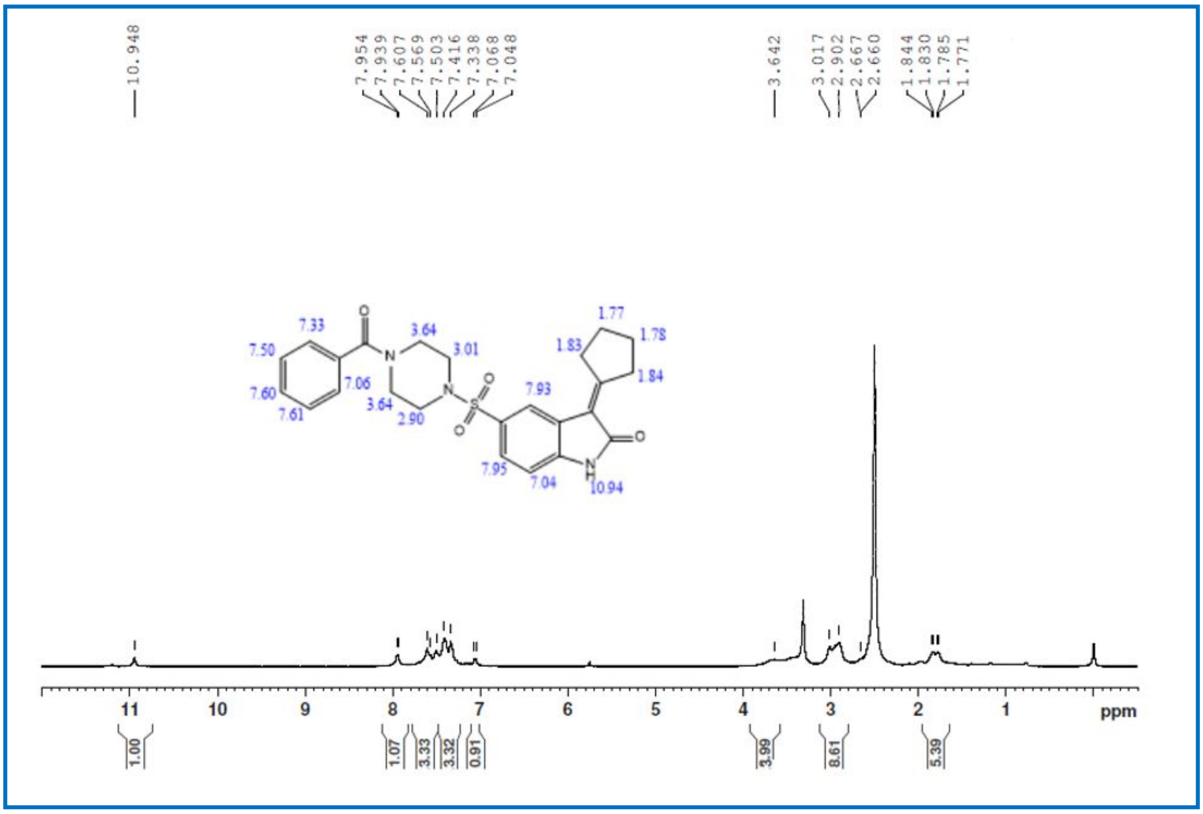

Mass of Compound-9d

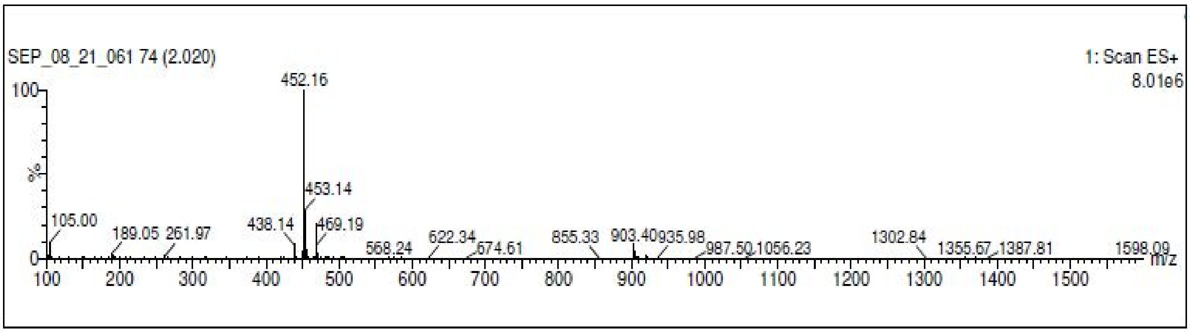

IR of Compound-9d

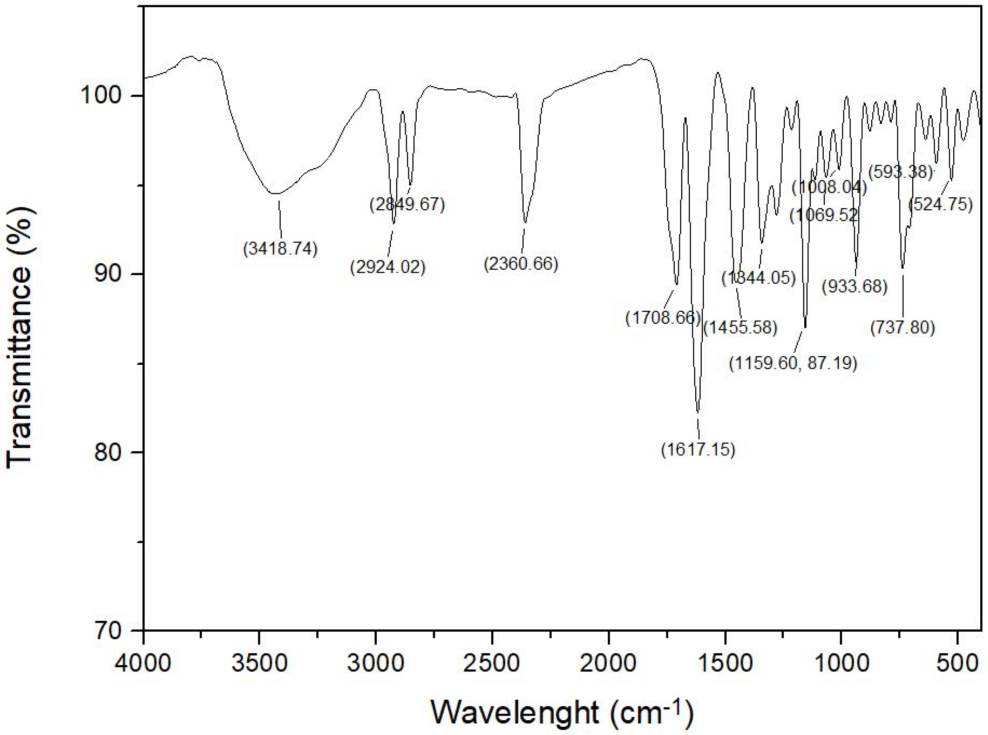

^{13}C -NMR of Compound-9d

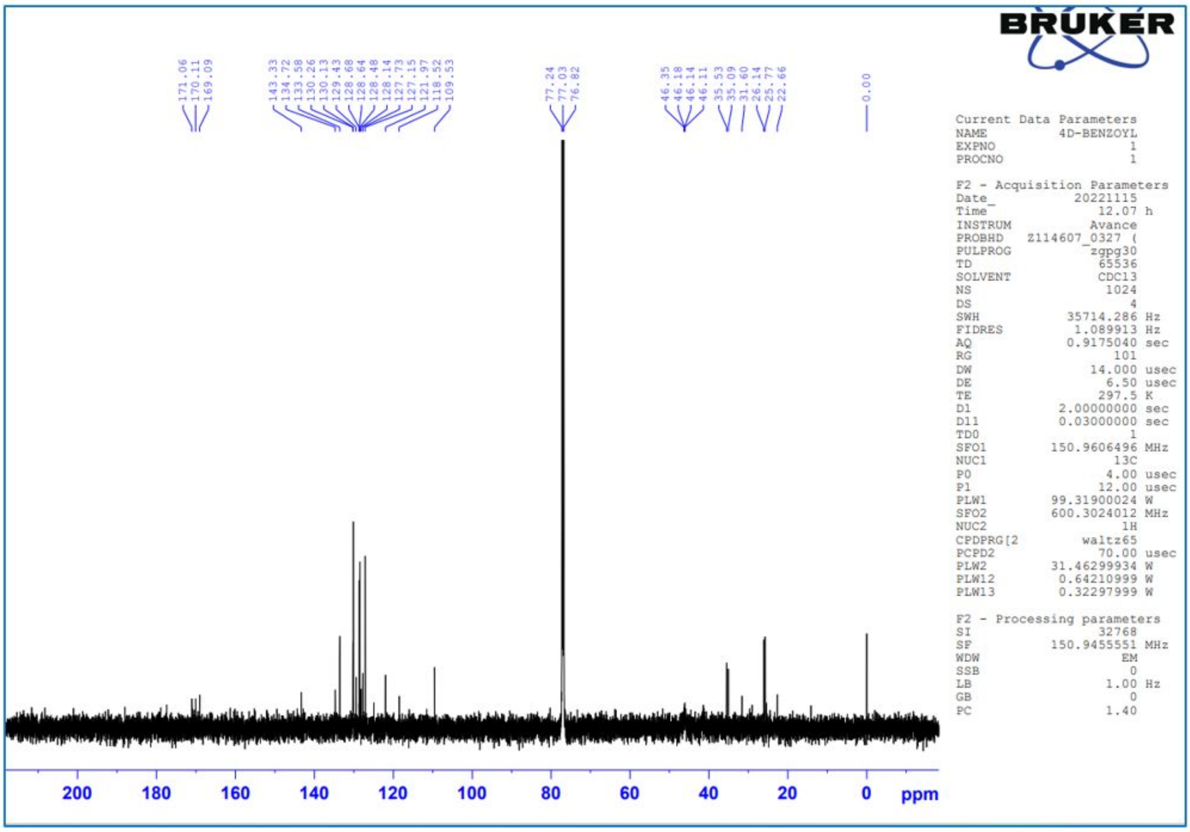

[1] H NMR of Compound-9e

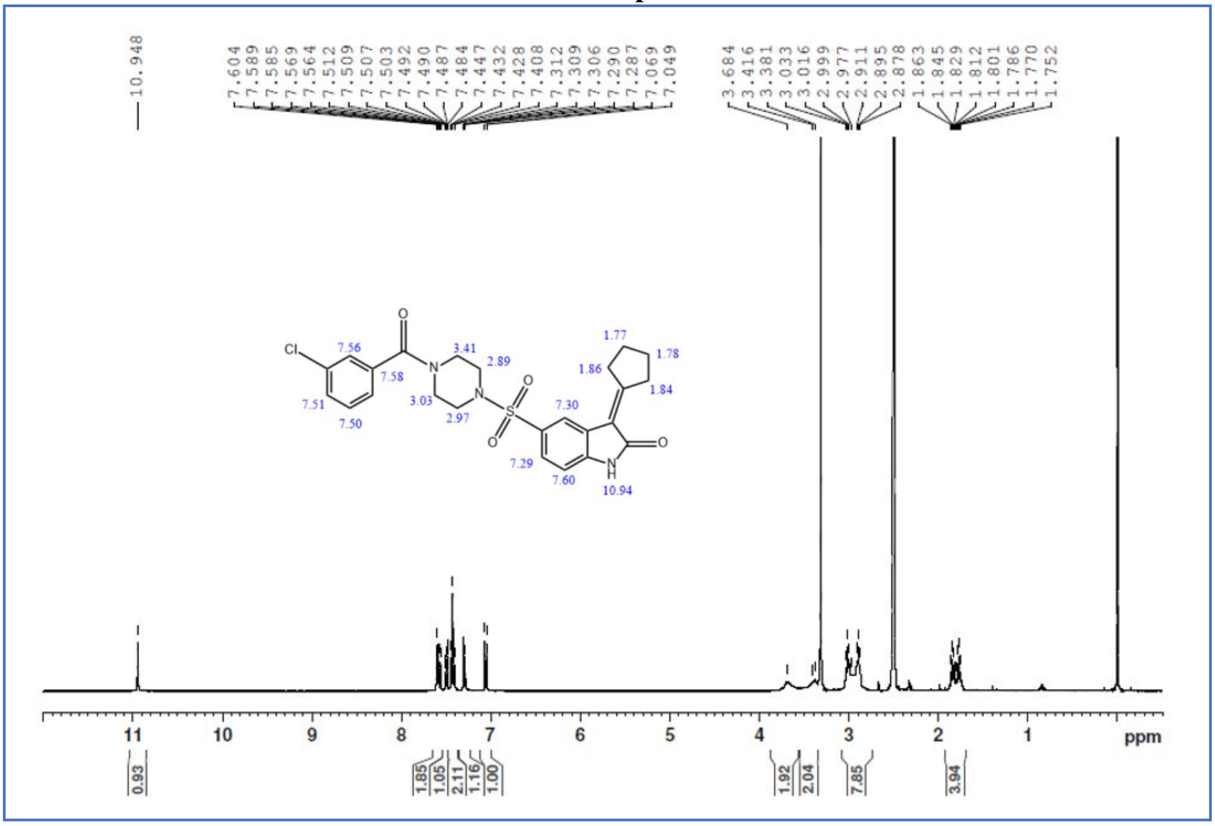

Mass of the Compound-9e

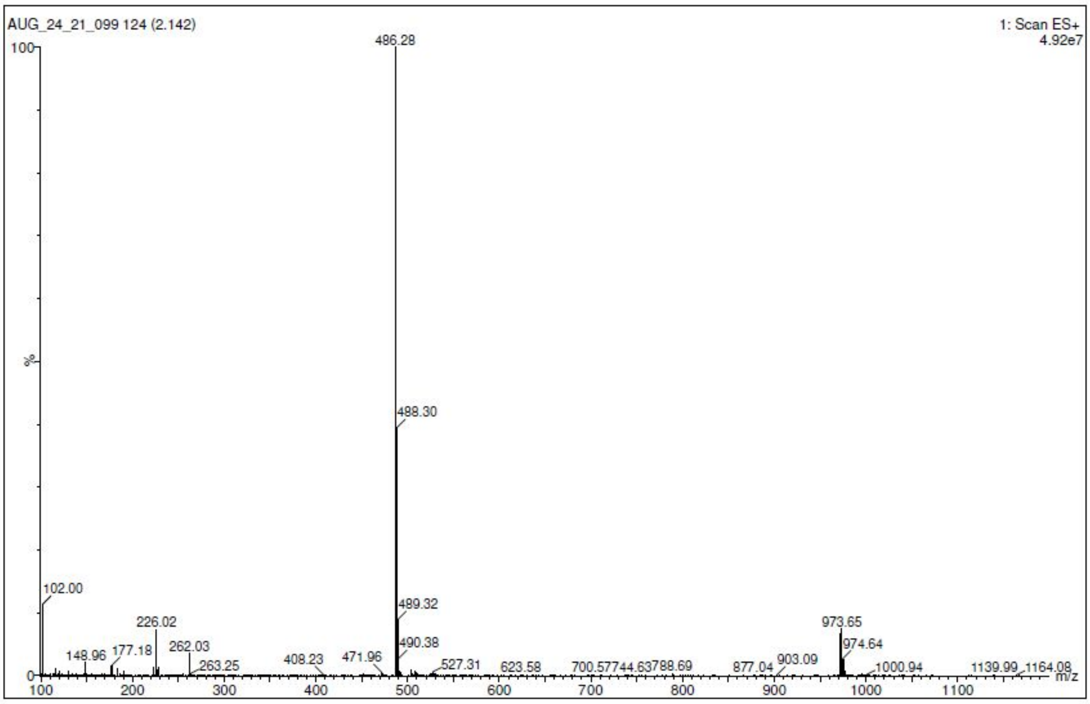

IR of the Compound-9e

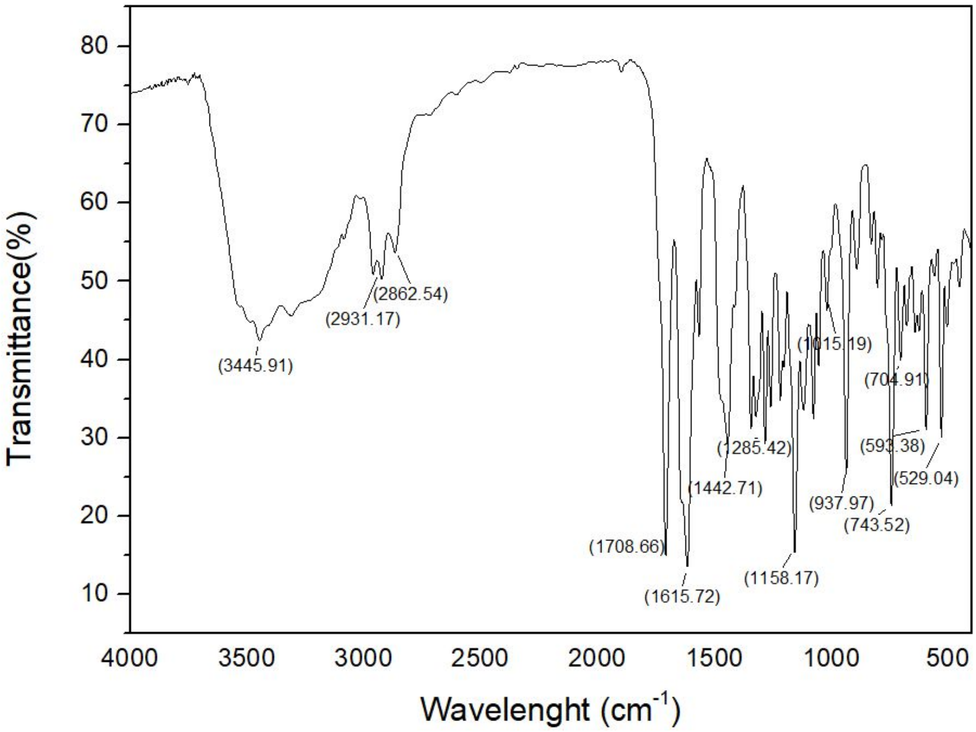

^{13}C -NMR of Compound-9e

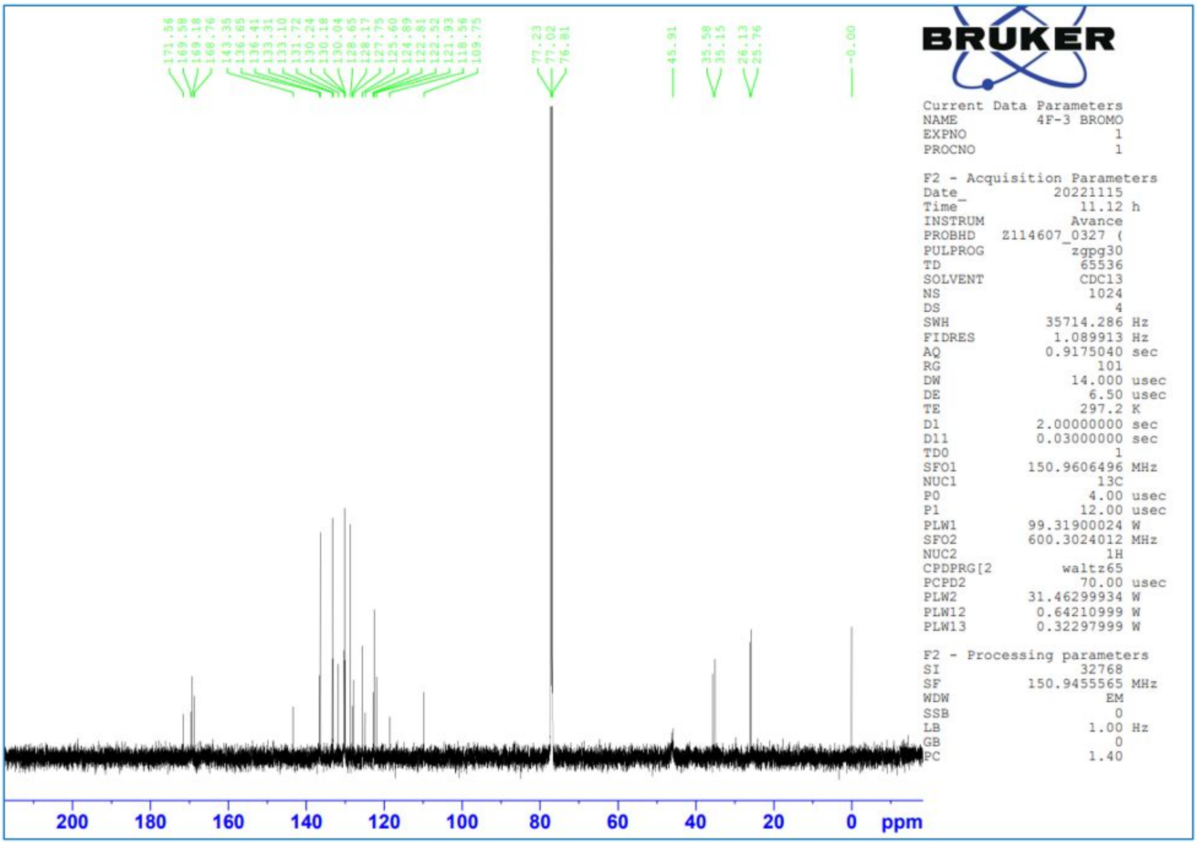

[1] H NMR of the Compound-9f

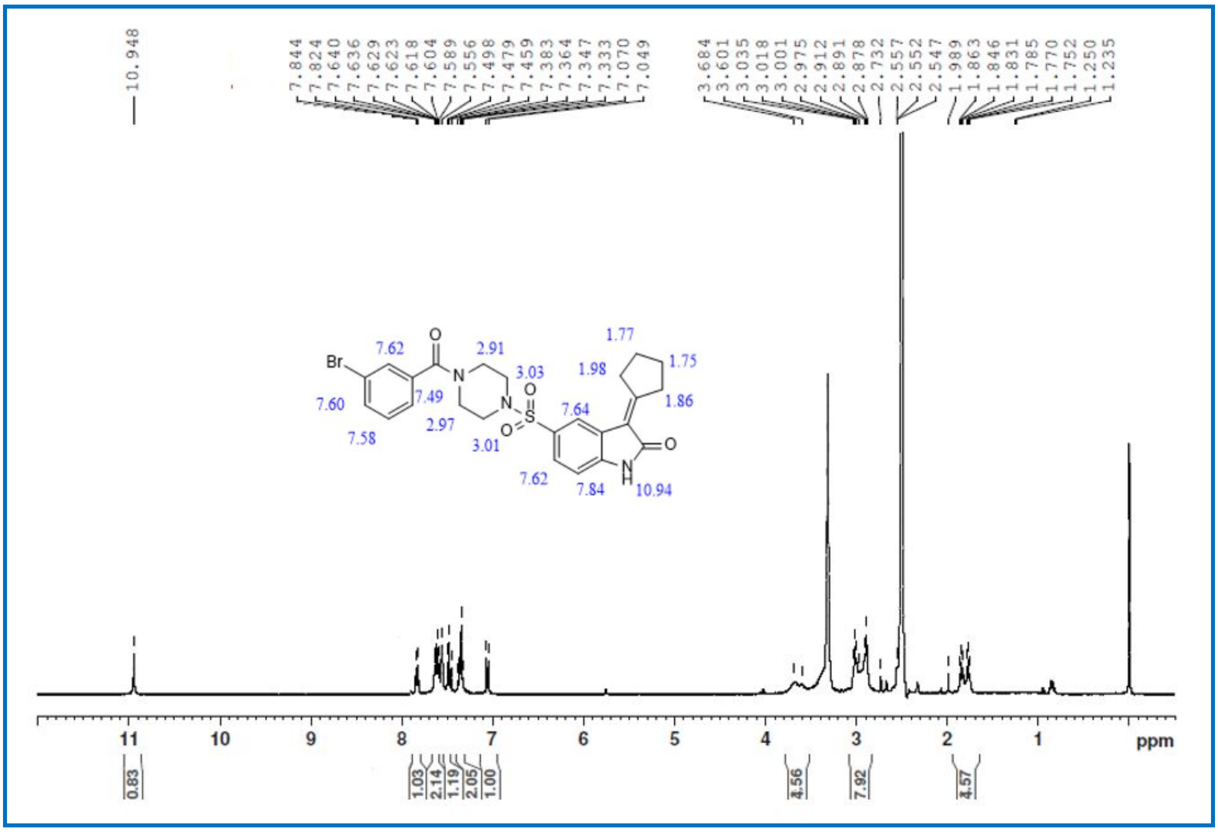

Mass of the Compound-9f

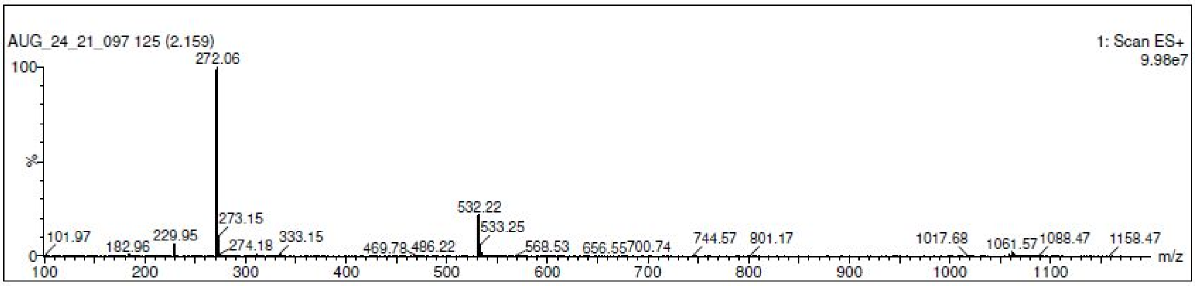

IR of the Compound-9f:

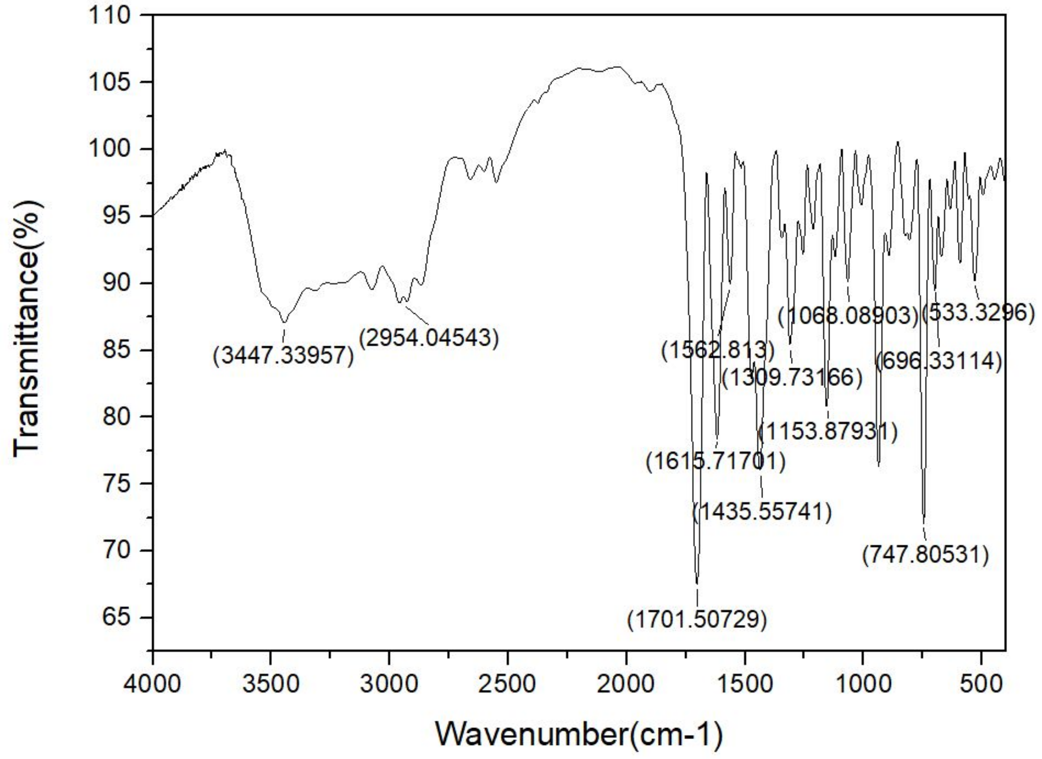

^{13}C -NMR of Compound-9f:

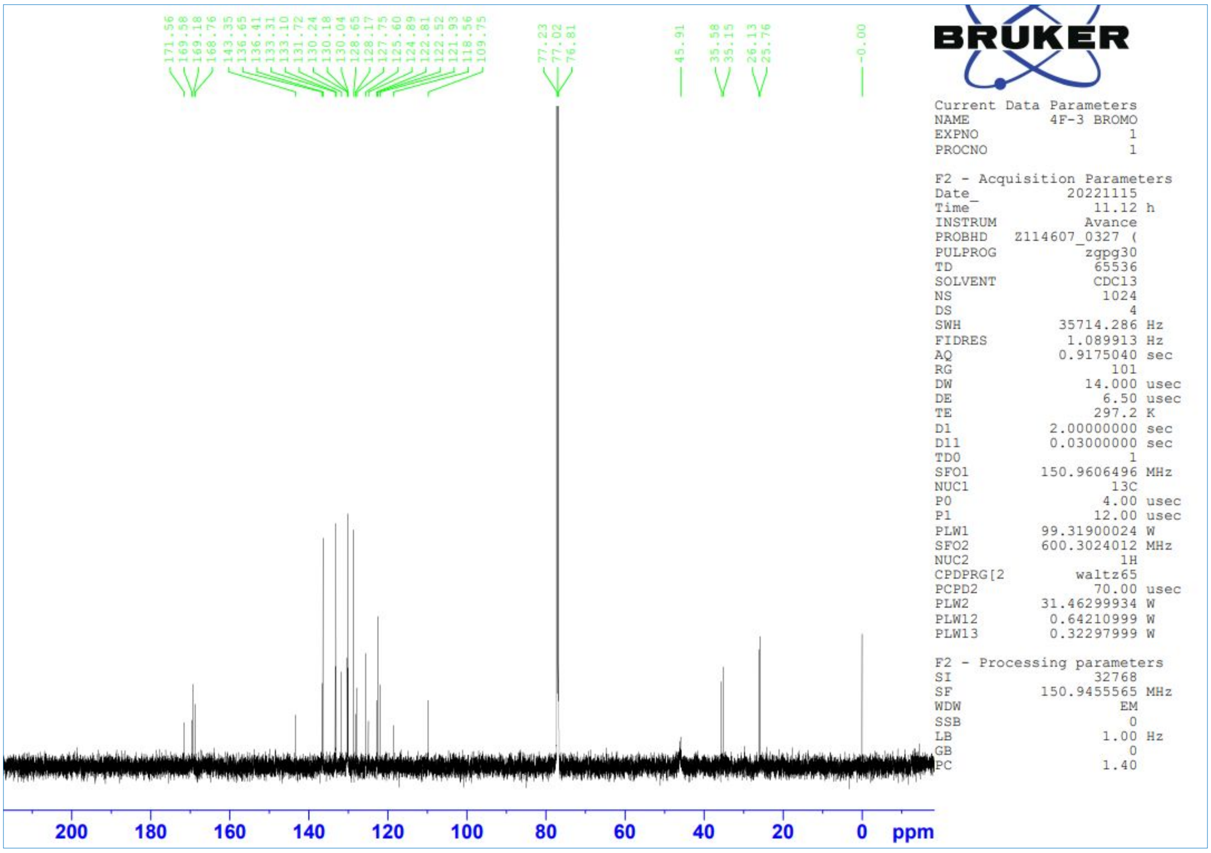

[1] H NMR of the Compound-9g

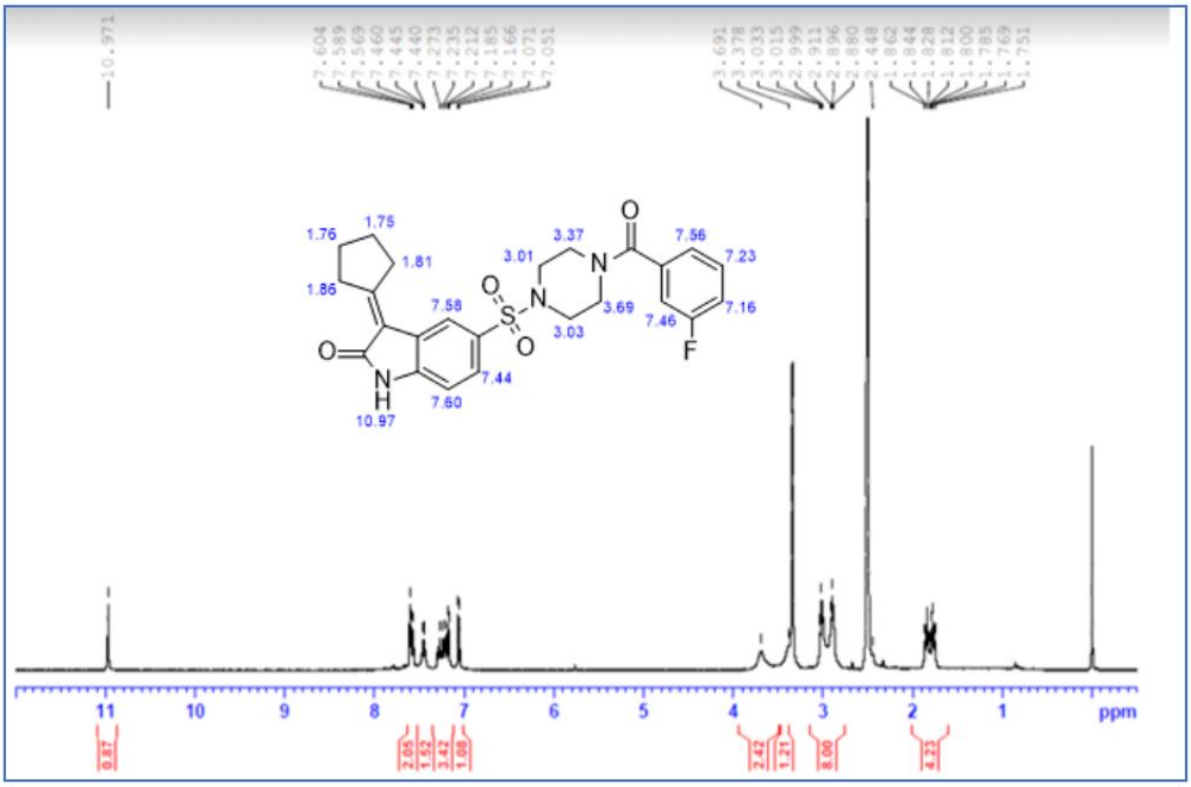

Mass of the Compound-9g

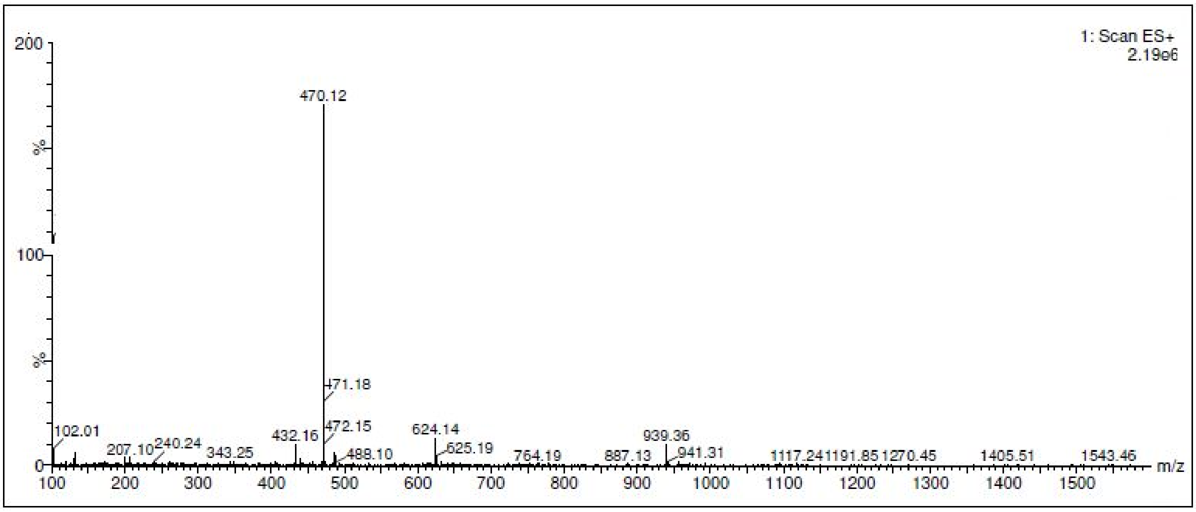

IR of the Compound-9g

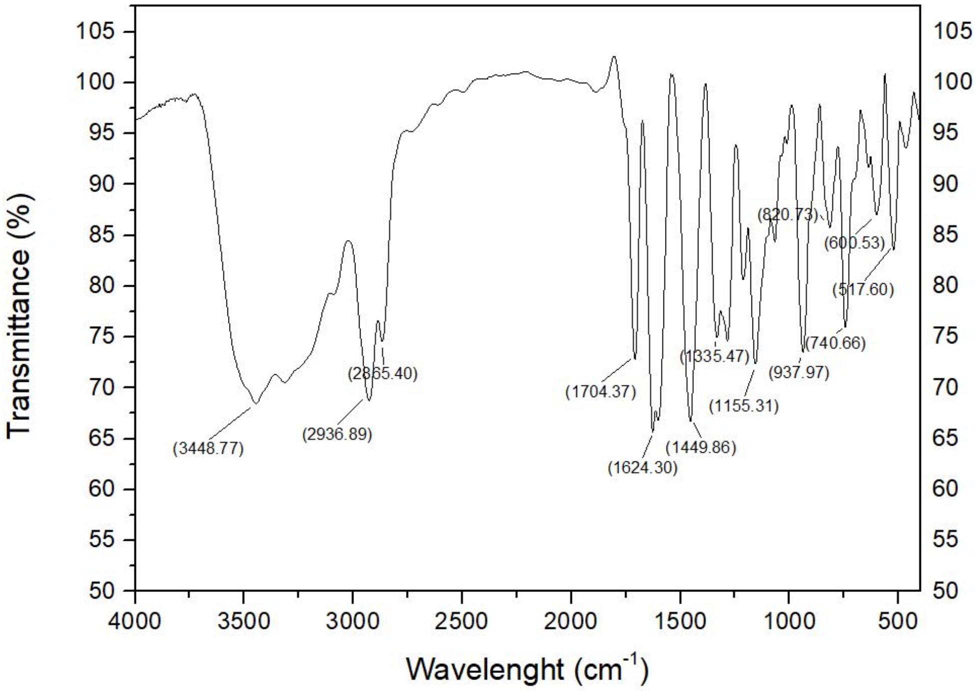

^{13}C -NMR of Compound-9g:

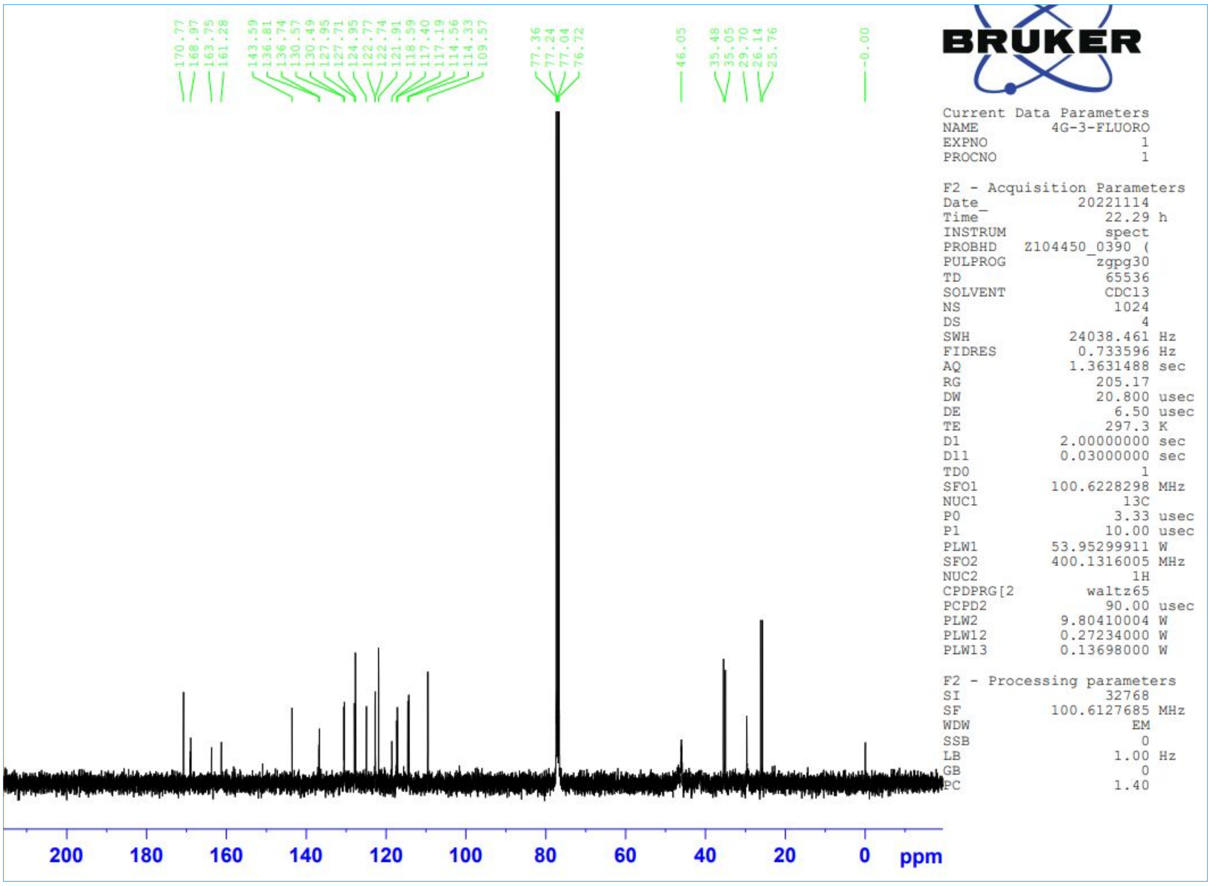

[1] H NMR of the Compound-9h

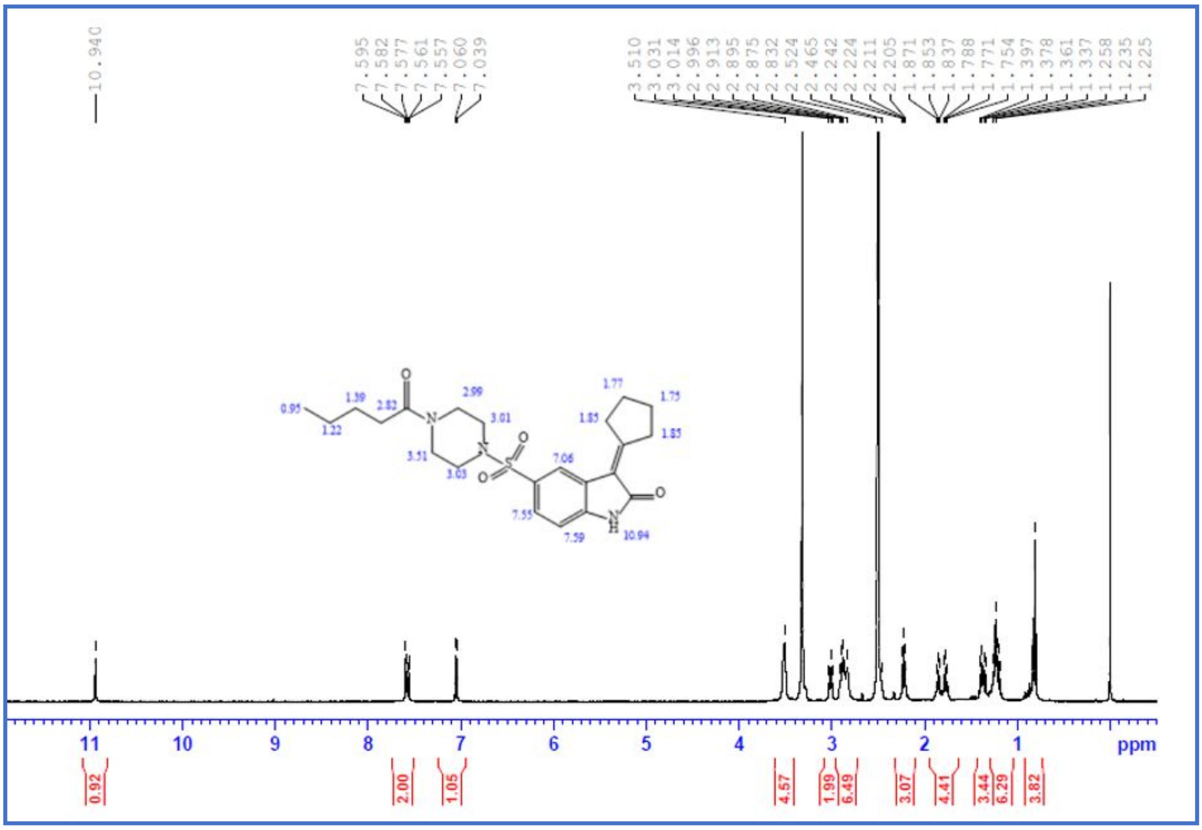

Mass of the Compound-9h

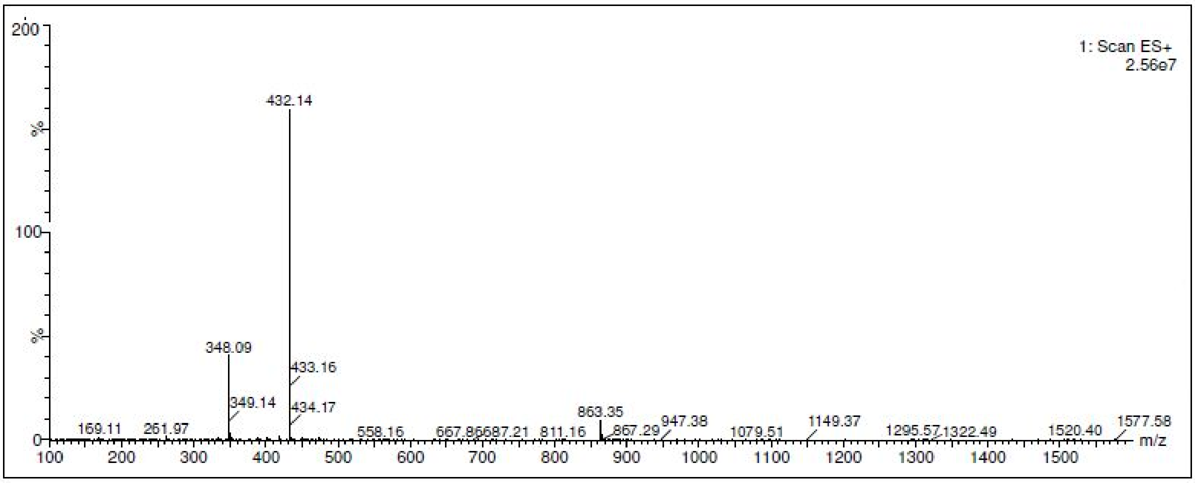

IR of the Compound-9h

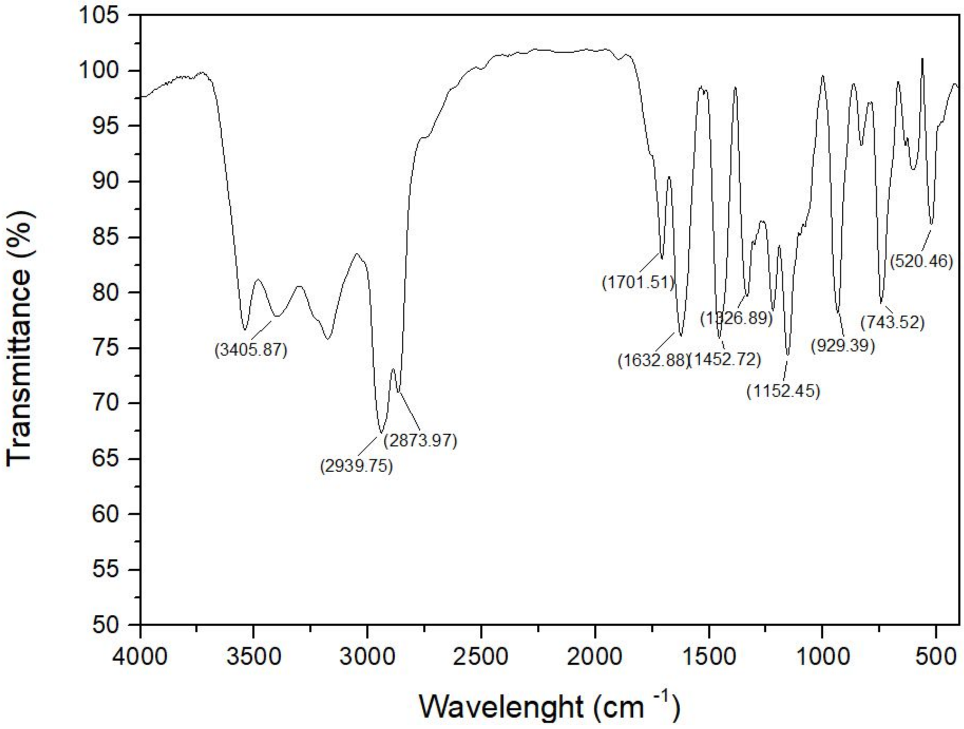

^{13}C -NMR of Compound-9h:

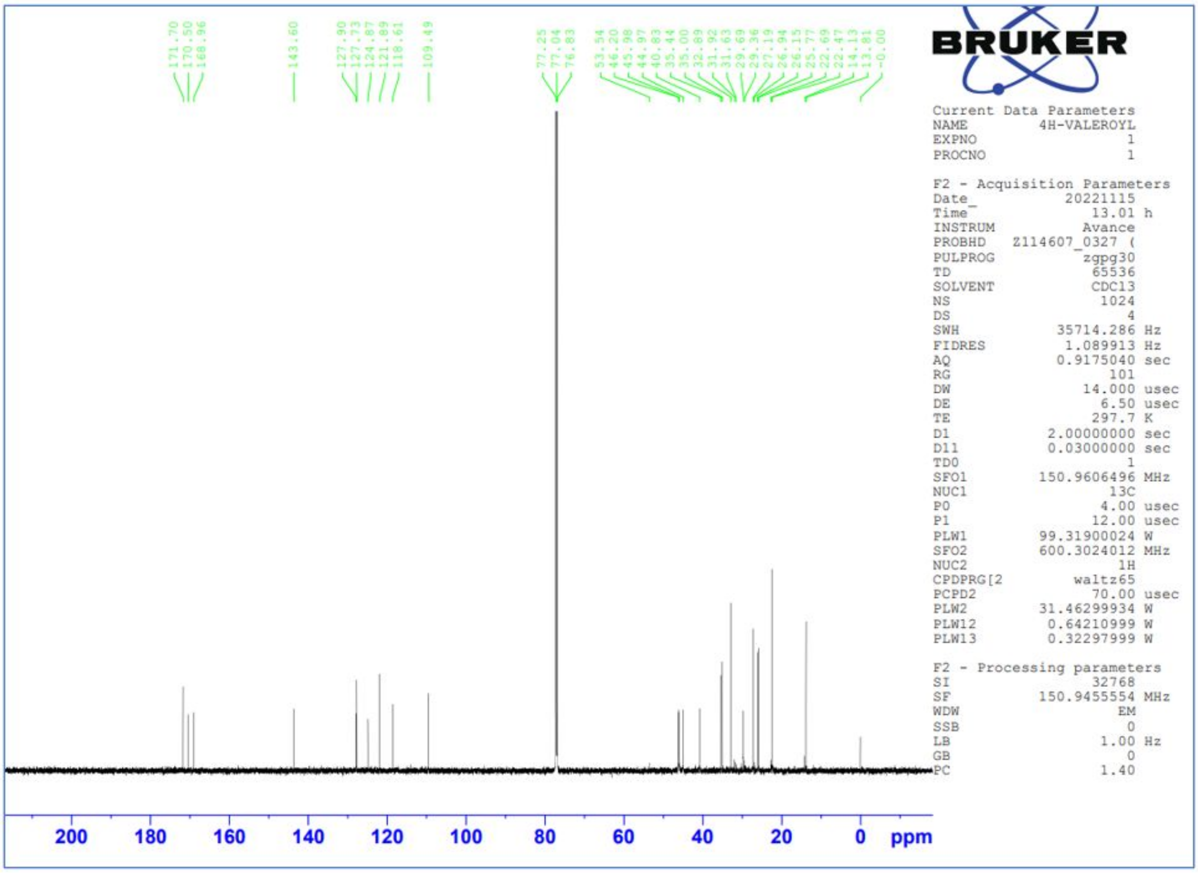

[1] H NMR of the Compound-9i

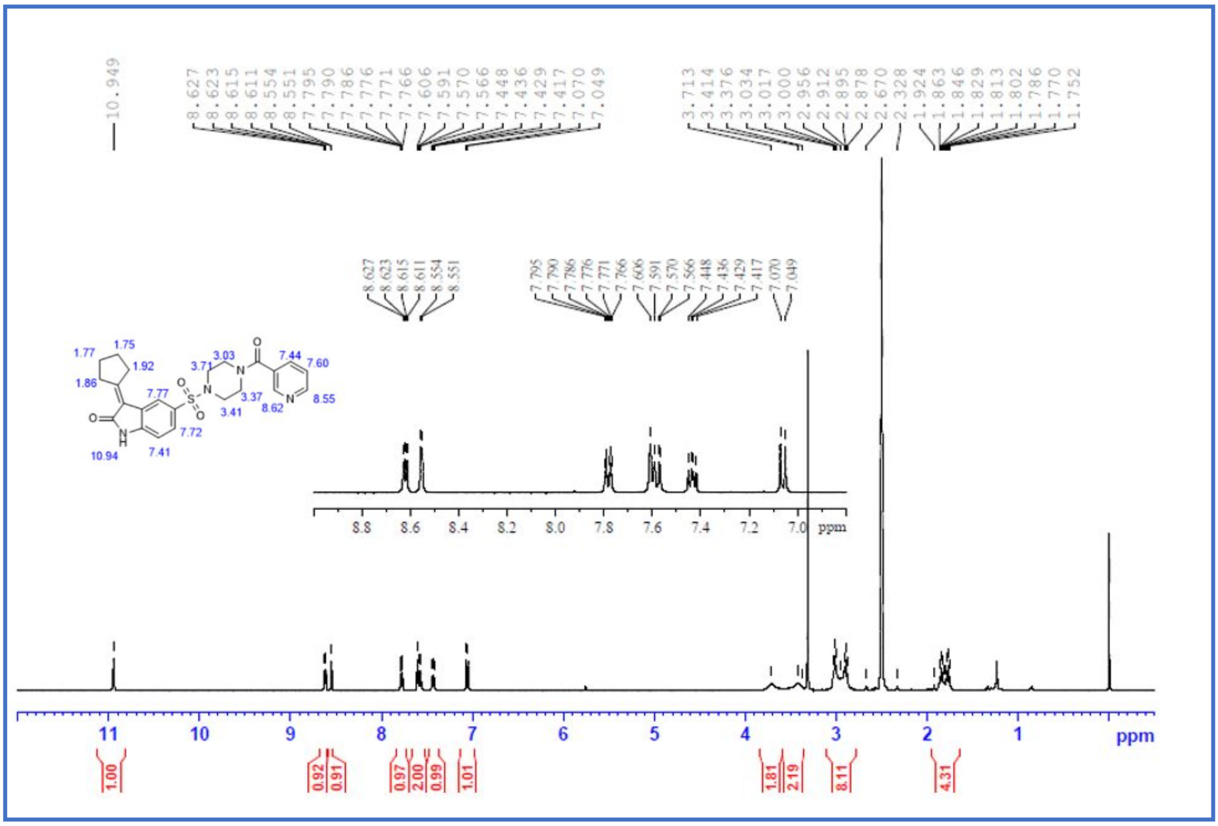

Mass of the Compound-9i

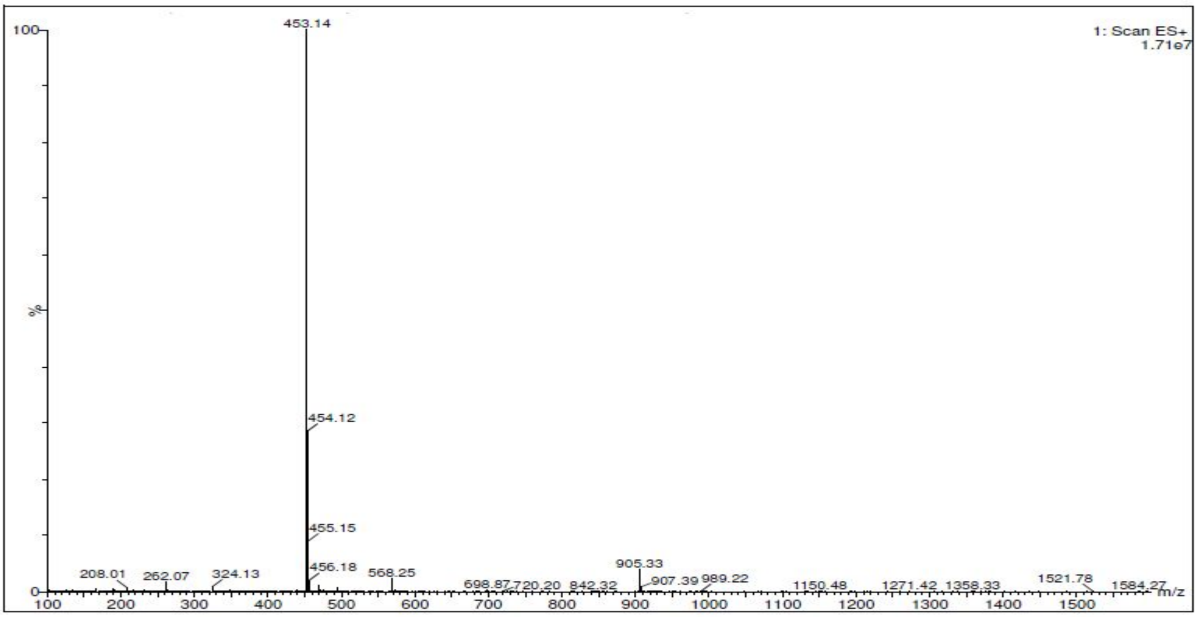

IR of the Compound-9h

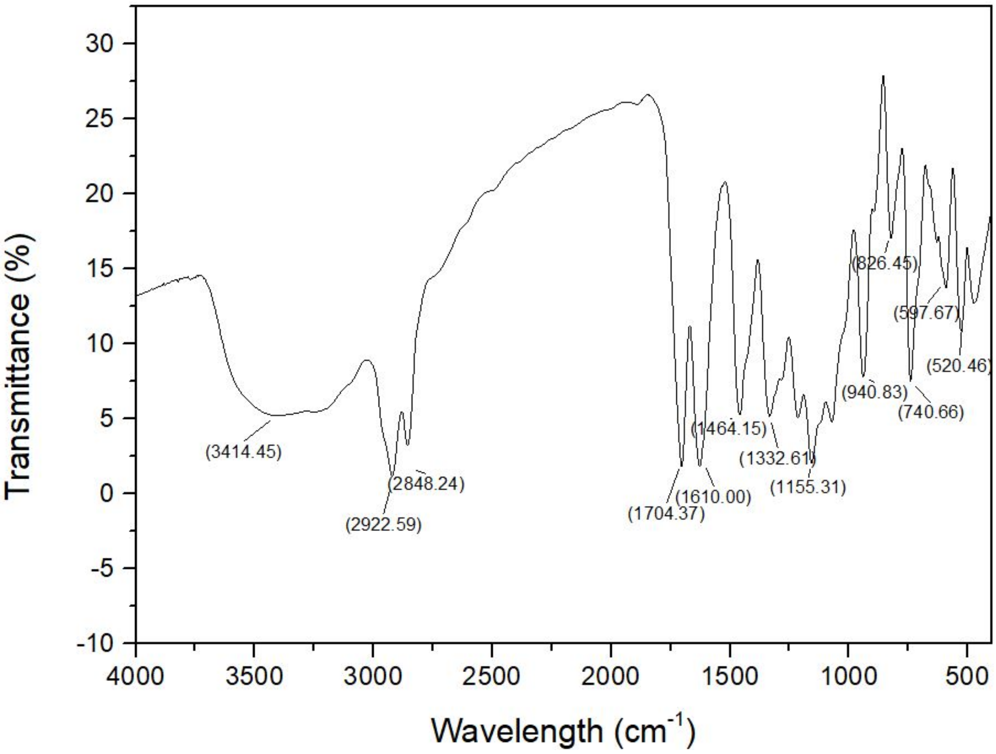

^{13}C NMR of the Compound-9i

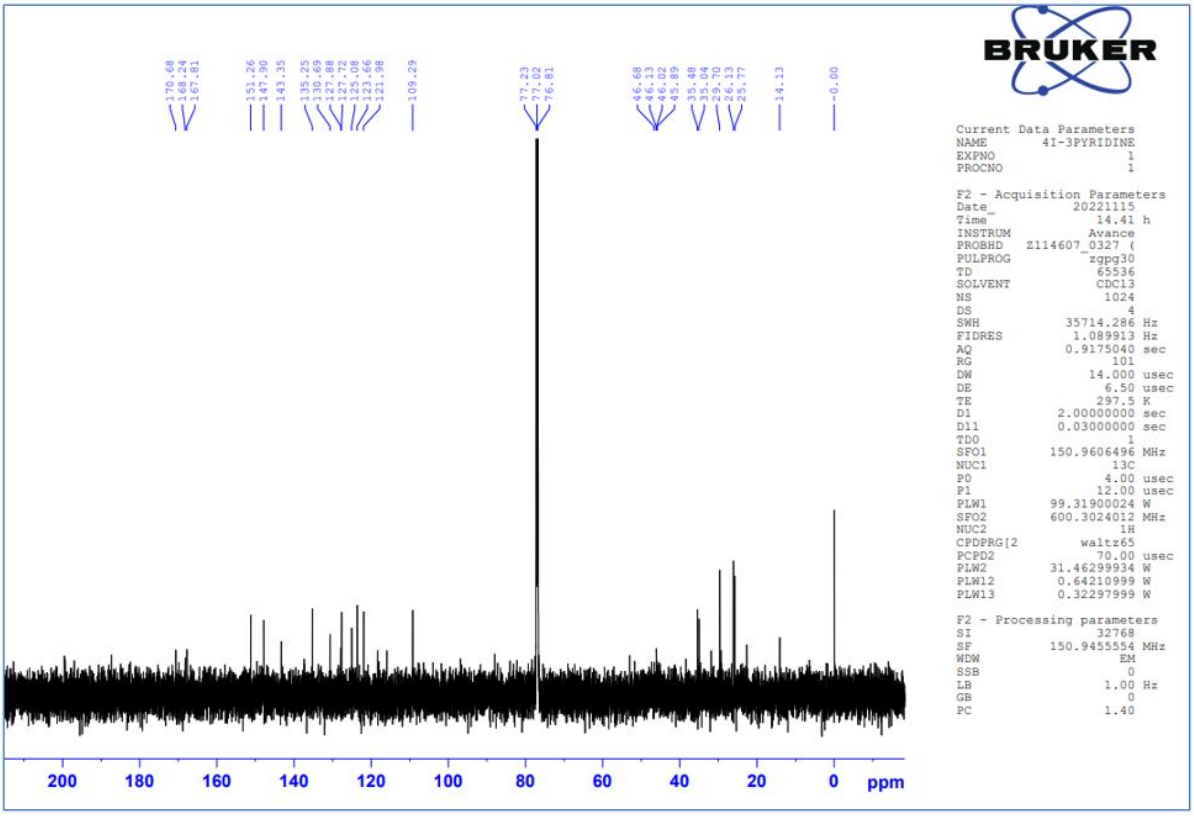

[1] H NMR of the Compound-9j

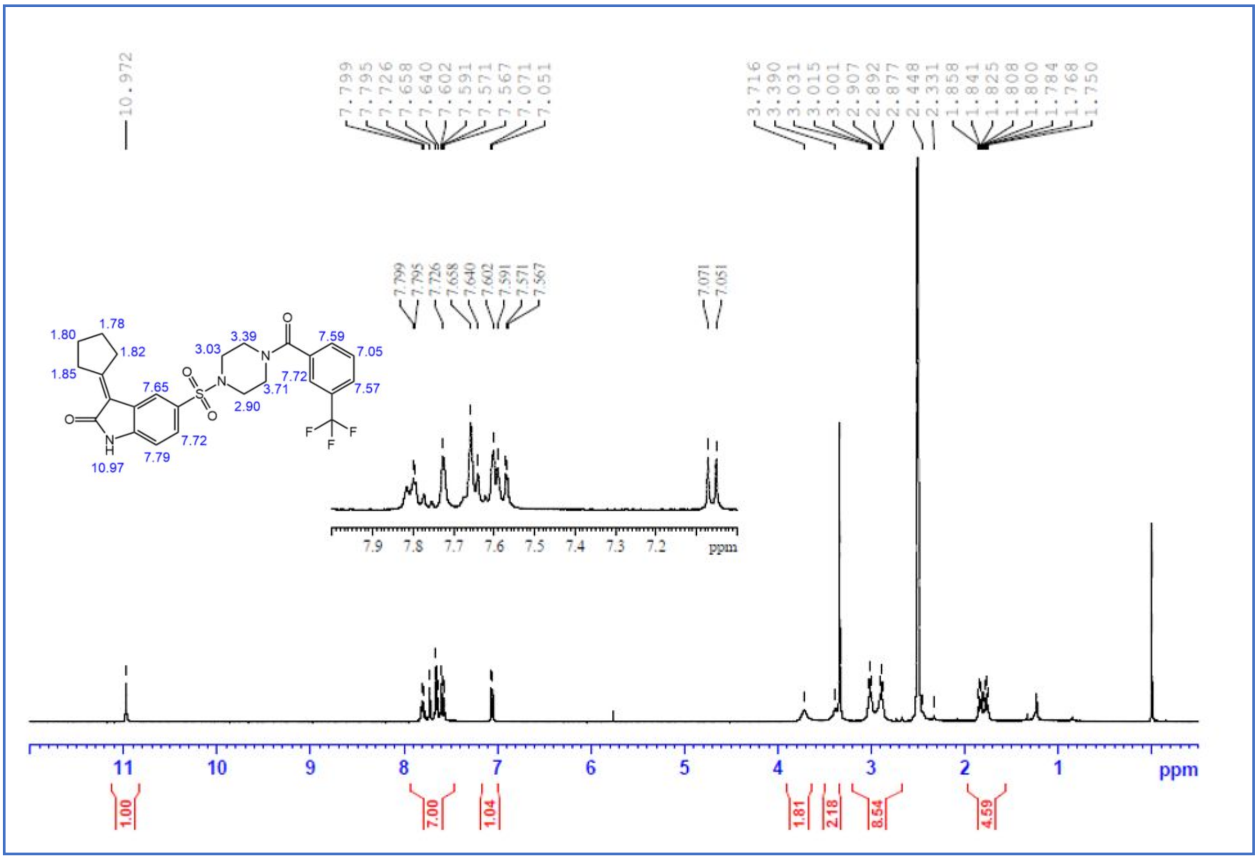

Mass of the Compound-9j

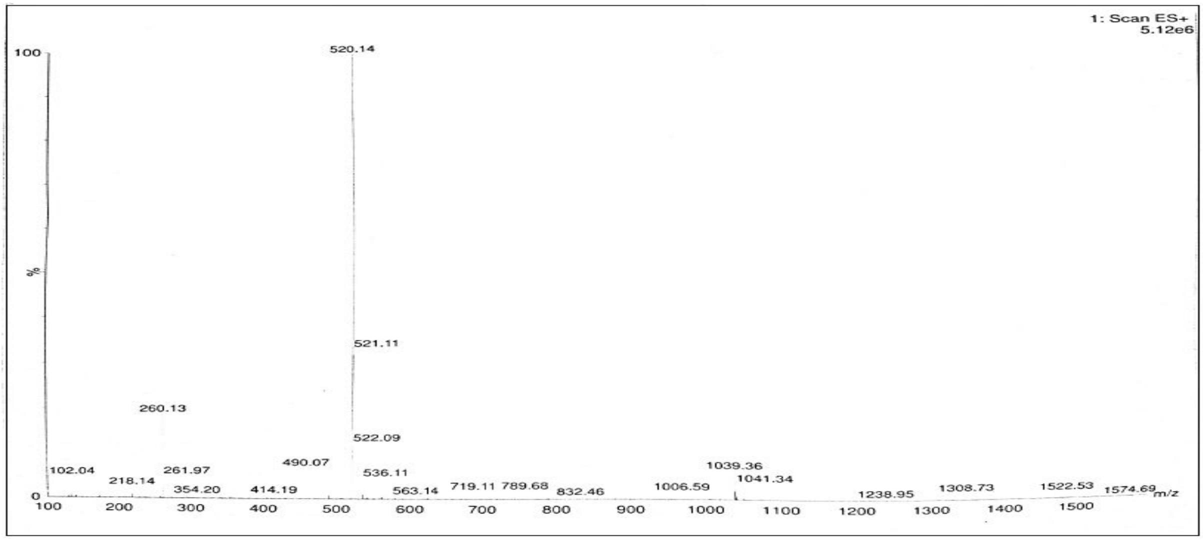

IR of the Compound-9j

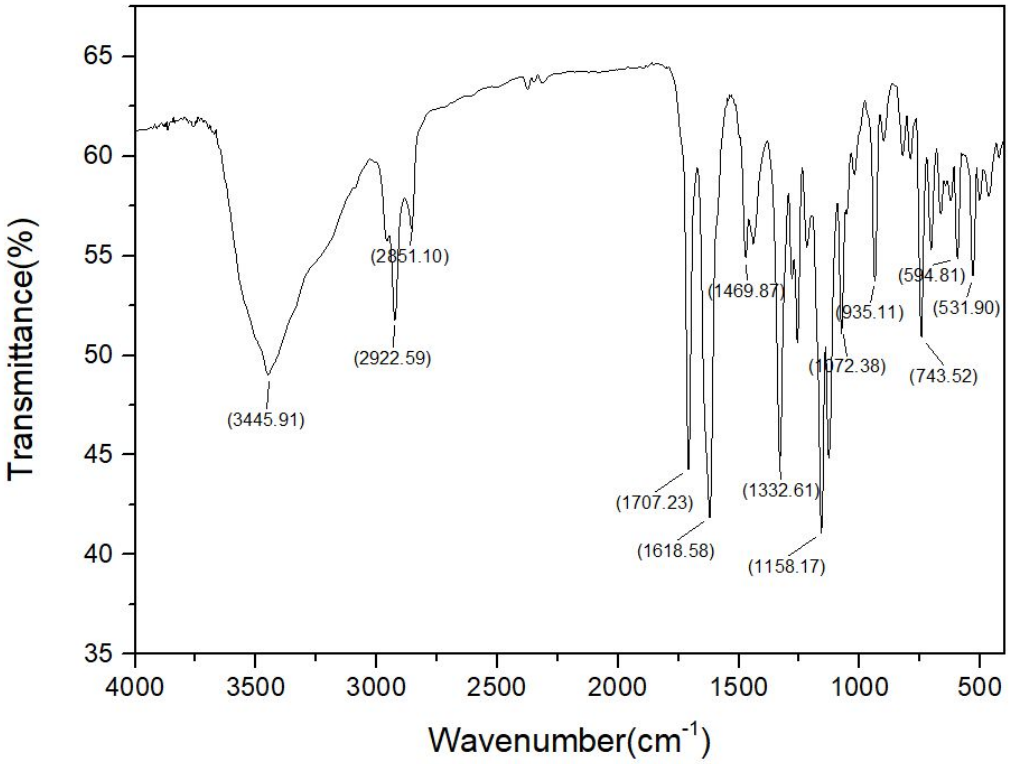

^{13}C NMR of the Compound-9j

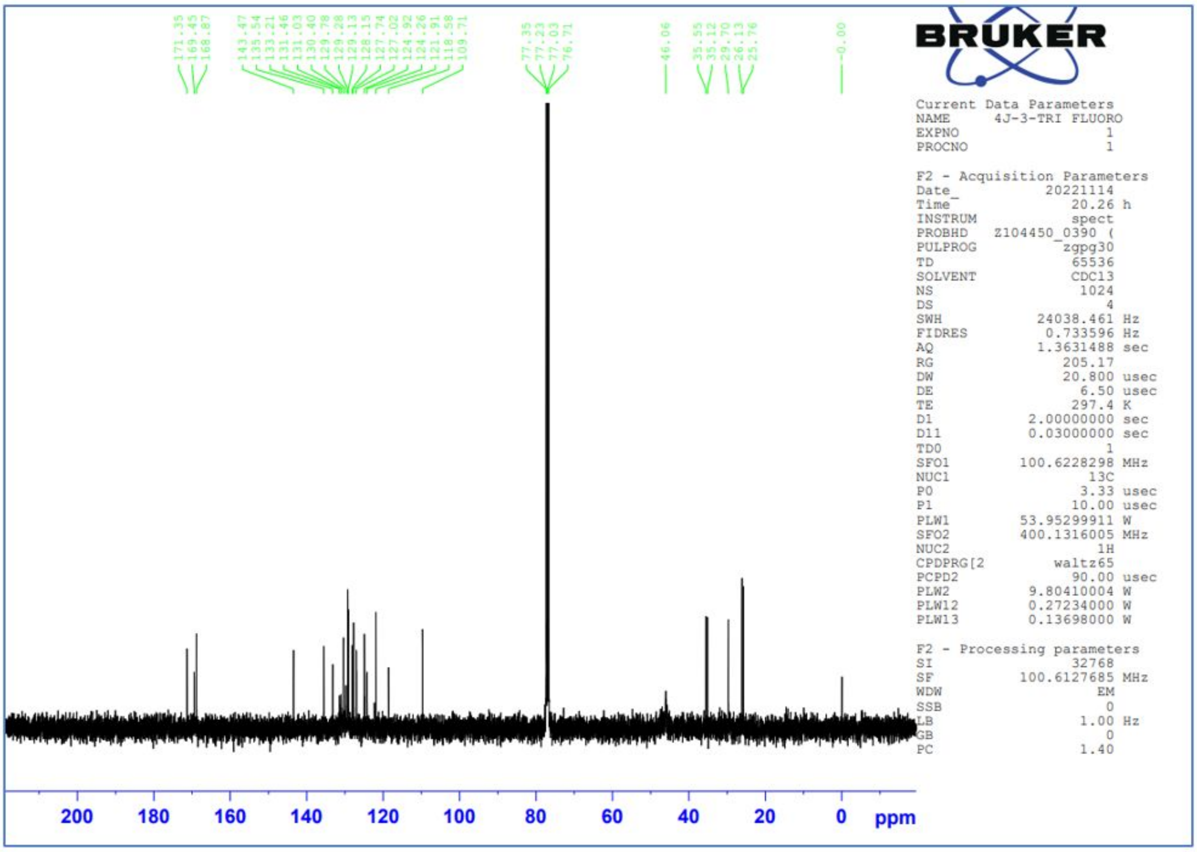

2. SUPPLEMENTARY FIGURES

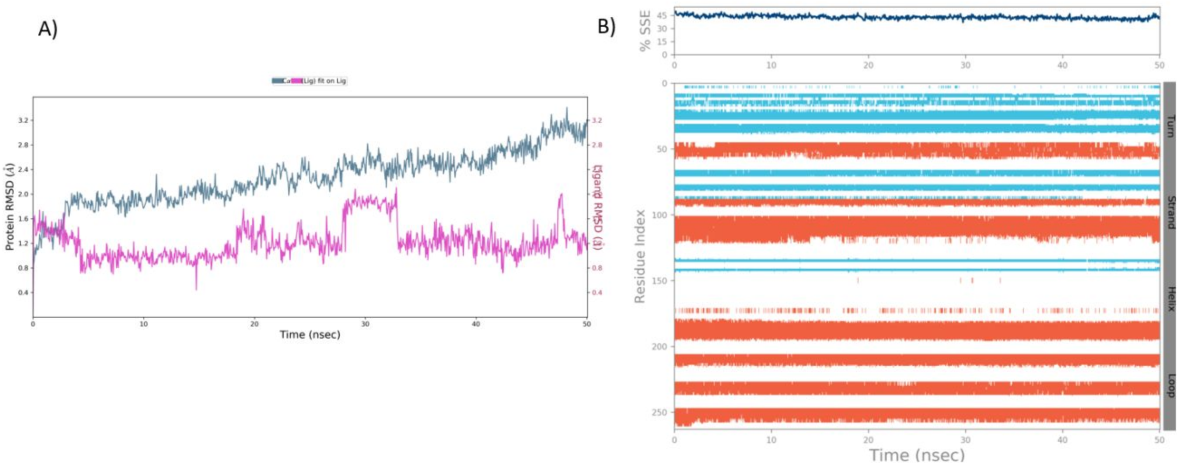

Figure S1. A) RMSD plot of protein (5P9J) and ligand (compound 9f) B) The secondary structure elements (SSE) of the protein are shown with helices in blue colour and beta strands in orange colour.

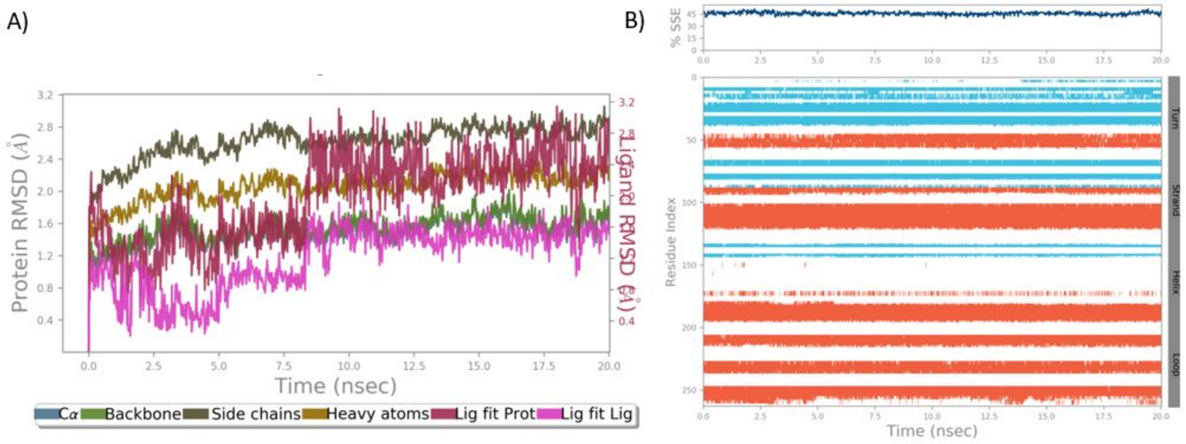

Figure S2. A) RMSD plot of protein (5P9J) and ligand (compound 9g) B) The secondary structure elements (SSE) of the protein are shown with helices in blue colour and beta strands in orange colour.

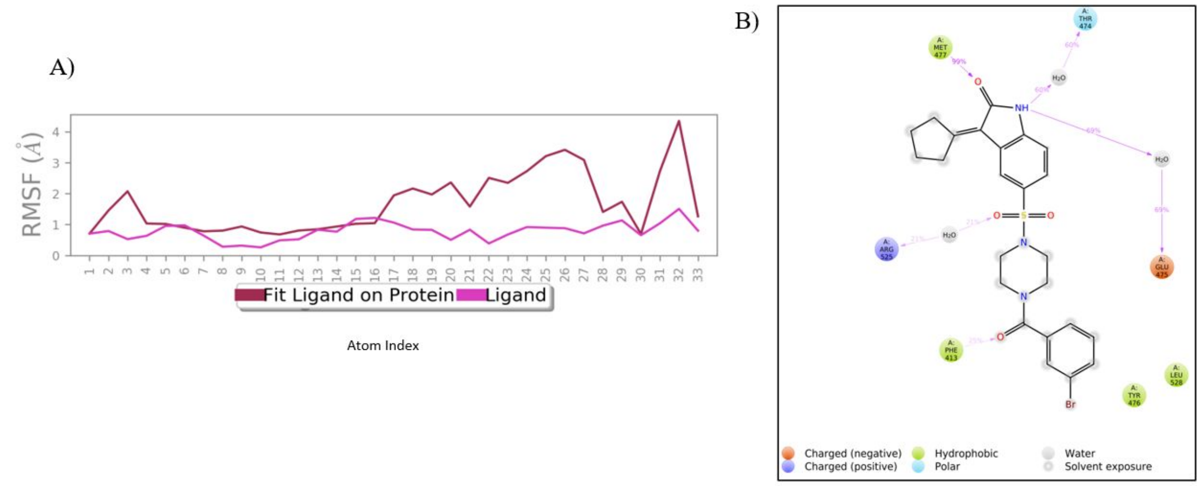

Figure S3. A) ligand-RMSF plot for compound 9f- protein 5P9J, where the brown line indicates the ligand fluctuations with respect to the binding site residues present on the target protein, and the pink line shows the fluctuations where the ligand in each frame is aligned on the ligand in the first reference frame. B) The compound **9f** shows interacting residues.

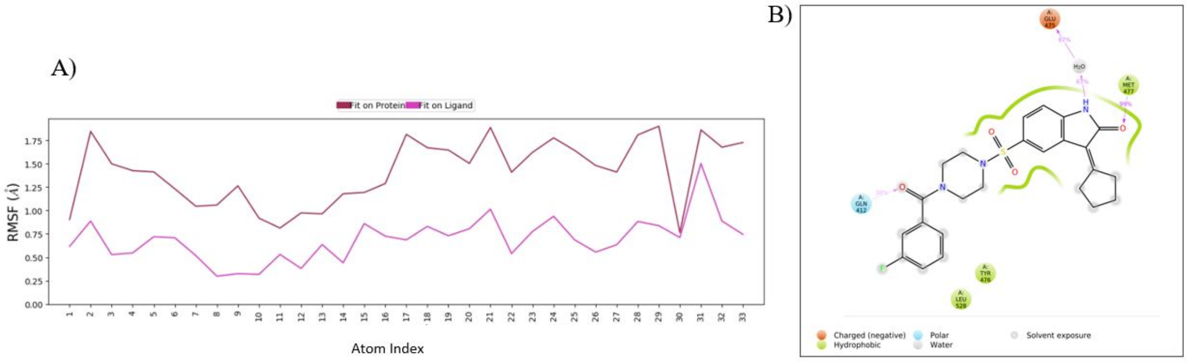

Figure S4. A) ligand-RMSF plot for compound 9g- protein 5P9J, where the brown line indicates the ligand fluctuations with respect to the binding site residues present on the target protein, and the pink line shows the fluctuations where the ligand in each frame is aligned on the ligand in the first reference frame. B) The compound **9g** shows interacting residues.

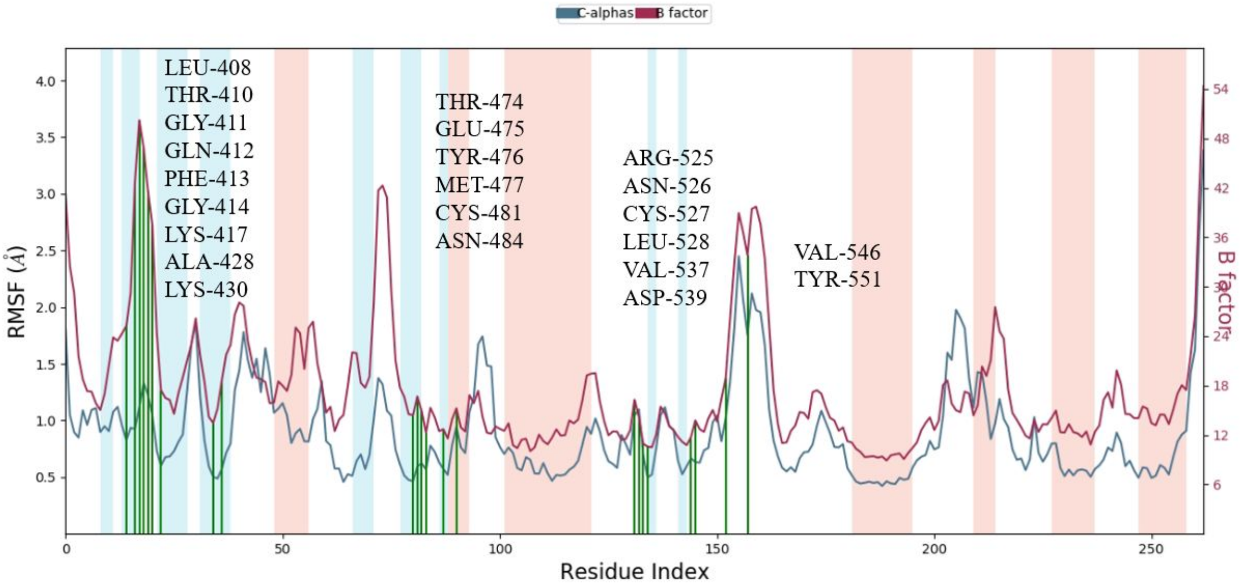

Figure S5. RMSF plot for Cα of 5P9J residues in compound **9h**-5P9J complex (Cα-blue colour, B factor-maroon colour), binding site residues in three-letter code (green colour).

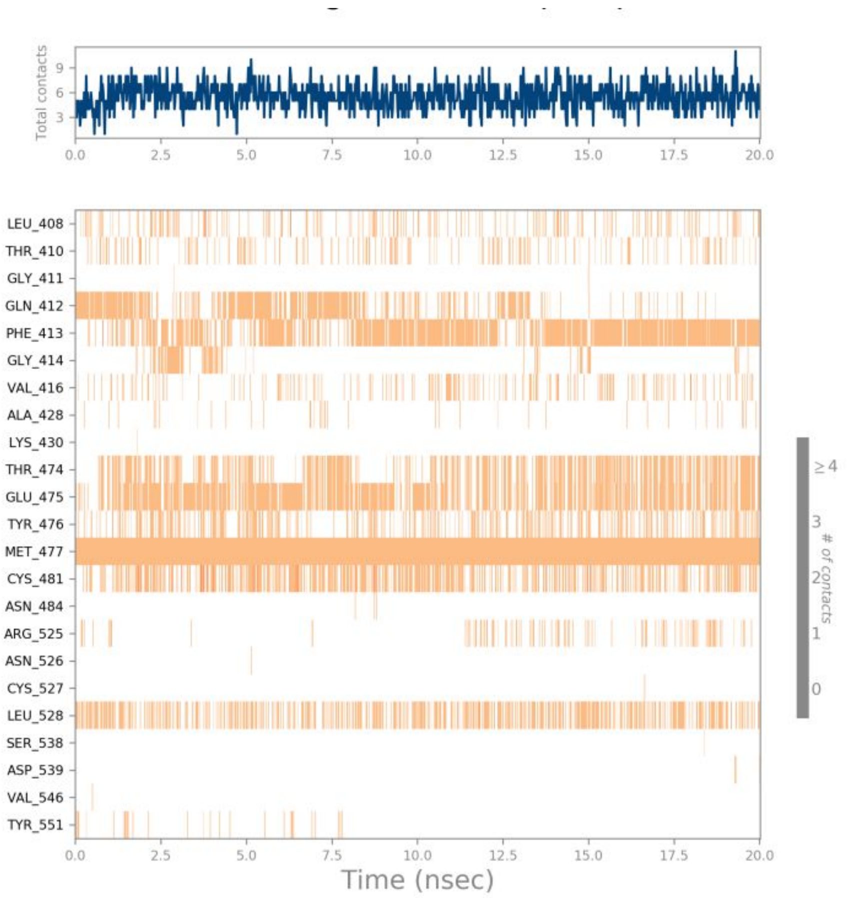

Figure S6. Timeline depiction of interactions and contacts (H-bonds, water bridges, hydrophobic) in the complex **9h**-5P9J during 20 ns MD simulation.

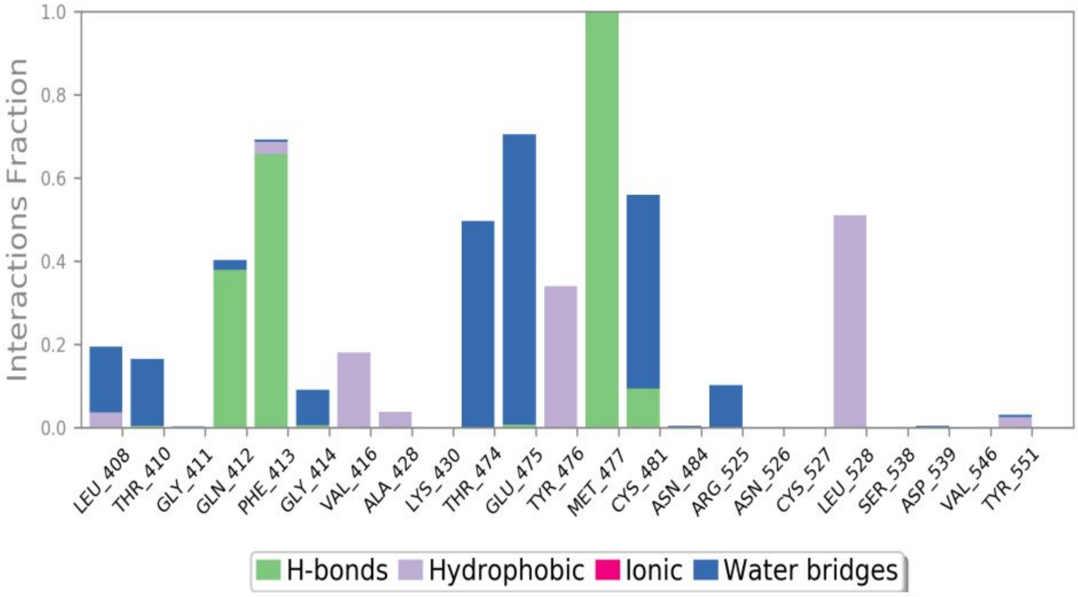

Figure S7. Histogram showing compound **9h** forming H-bond interactions (green colour), water bridges (blue colour), and hydrophobic interactions (violet colour) during 20 ns simulation.

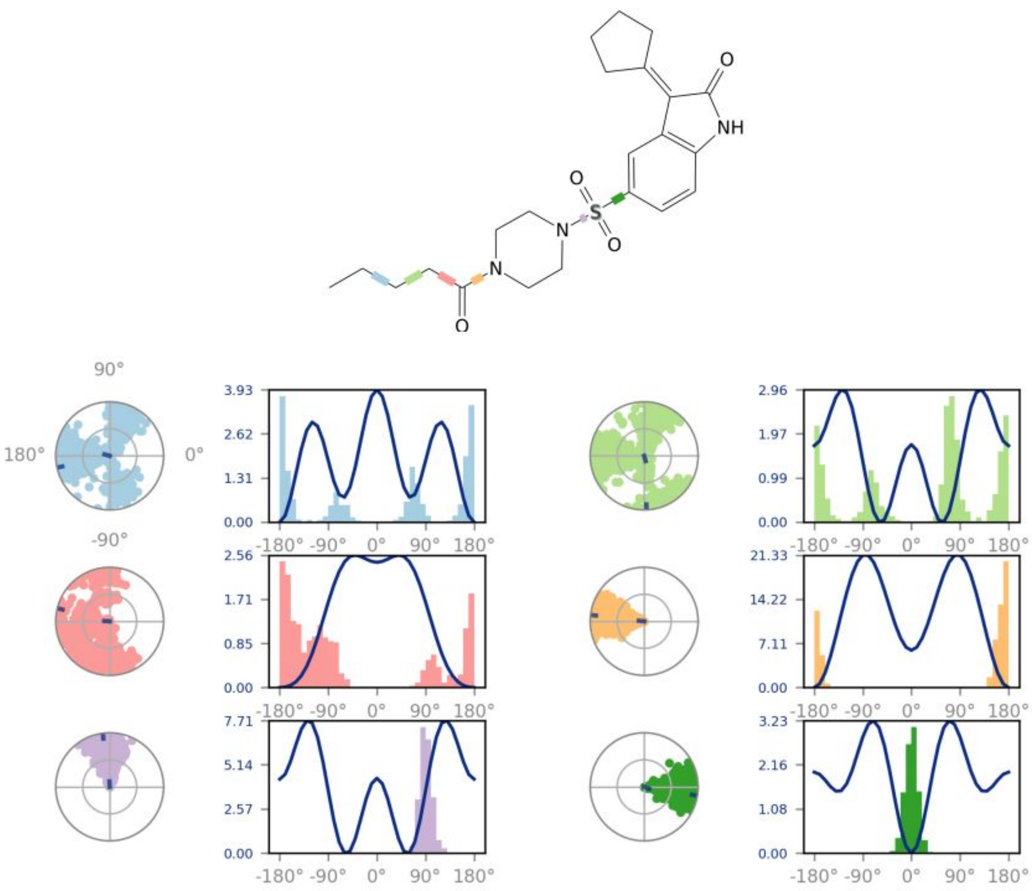

Figure S8. Torsional analysis of compound **9h** conformations during 20 ns simulations.

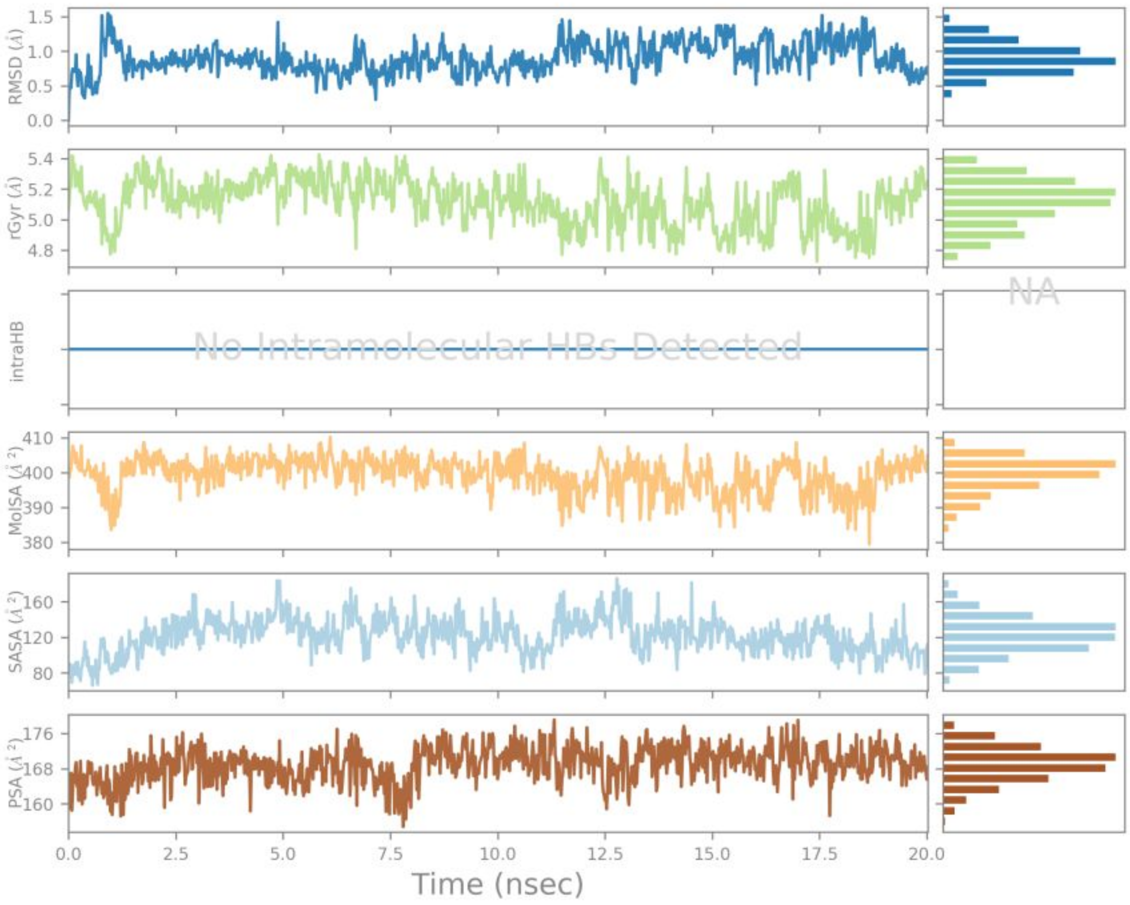

Figure S9. Showing six ligand properties during 20 ns simulations for compound **9h**: ligand RMSD (root mean square deviation), rGyr (radius of gyration), MolSA(molecular surface area), SASA(solvent accessible surface area), PSA (polar surface area).

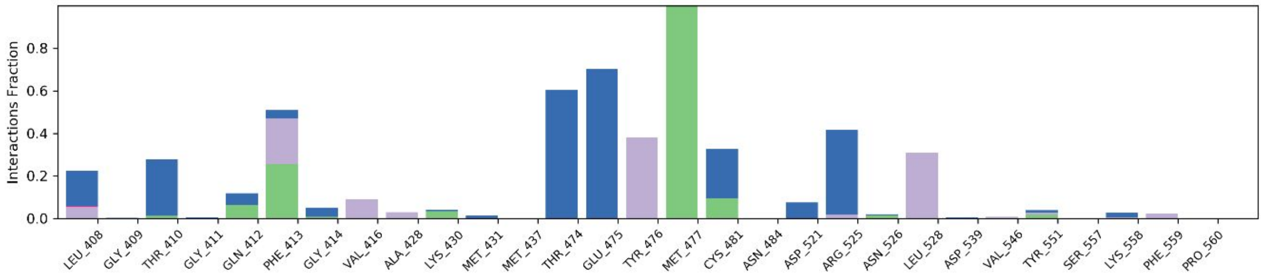

Figure S10. Histogram showing compound **9f** forming H-bond interactions (green colour), hydrophobic interactions (violet colour), and water bridges (blue colour) during 50 ns simulation.

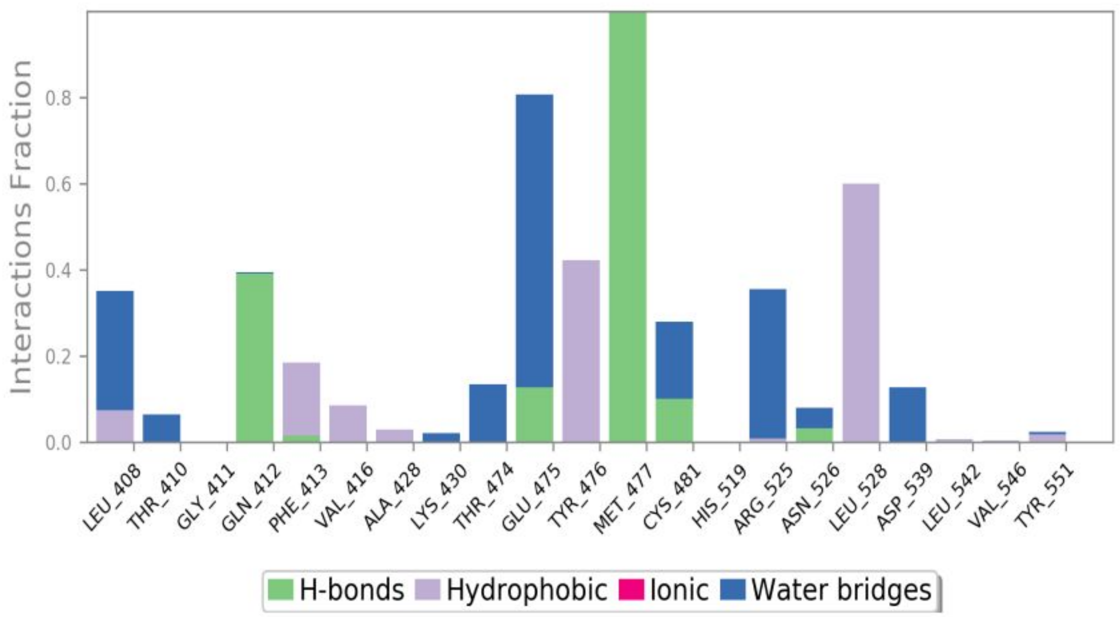

Figure S11. Histogram showing compound **9g** forming H-bond interactions (green colour), hydrophobic interactions (violet colour), and water bridges (blue colour) during 20 ns simulation.

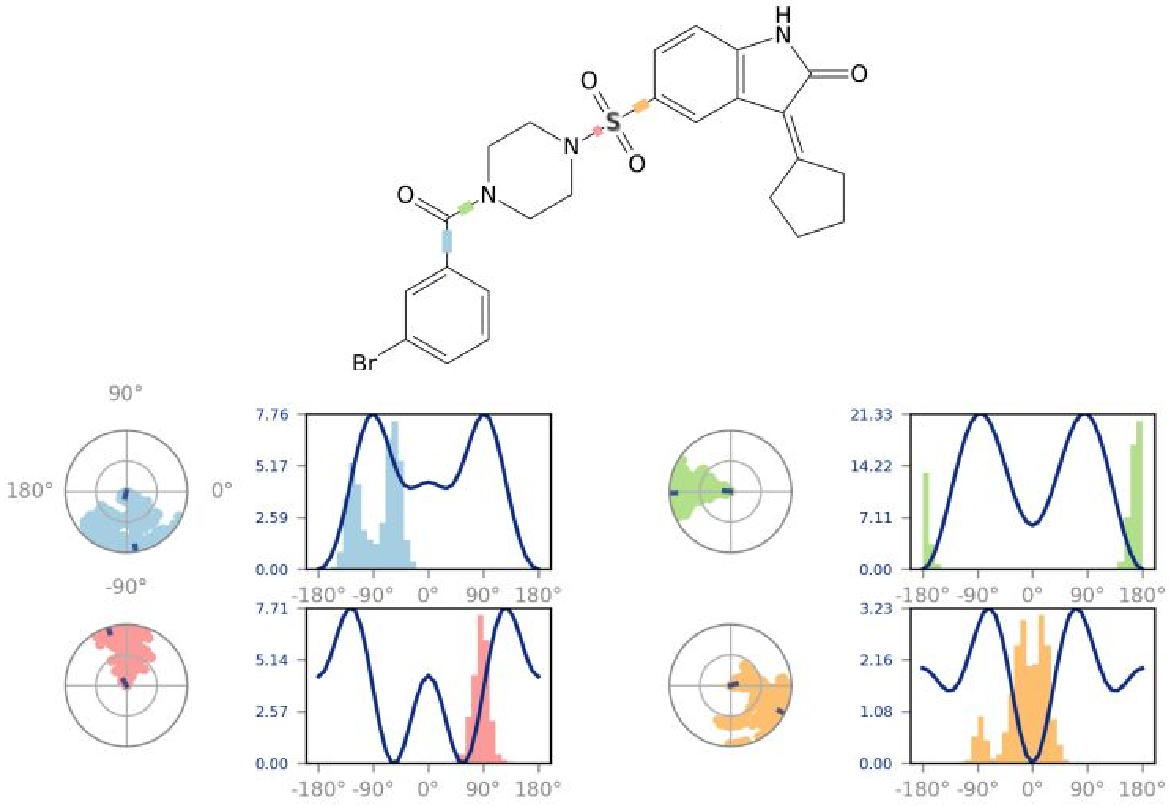

Figure S12. Torsional analysis of compound **9f** conformations during 50 ns simulations.

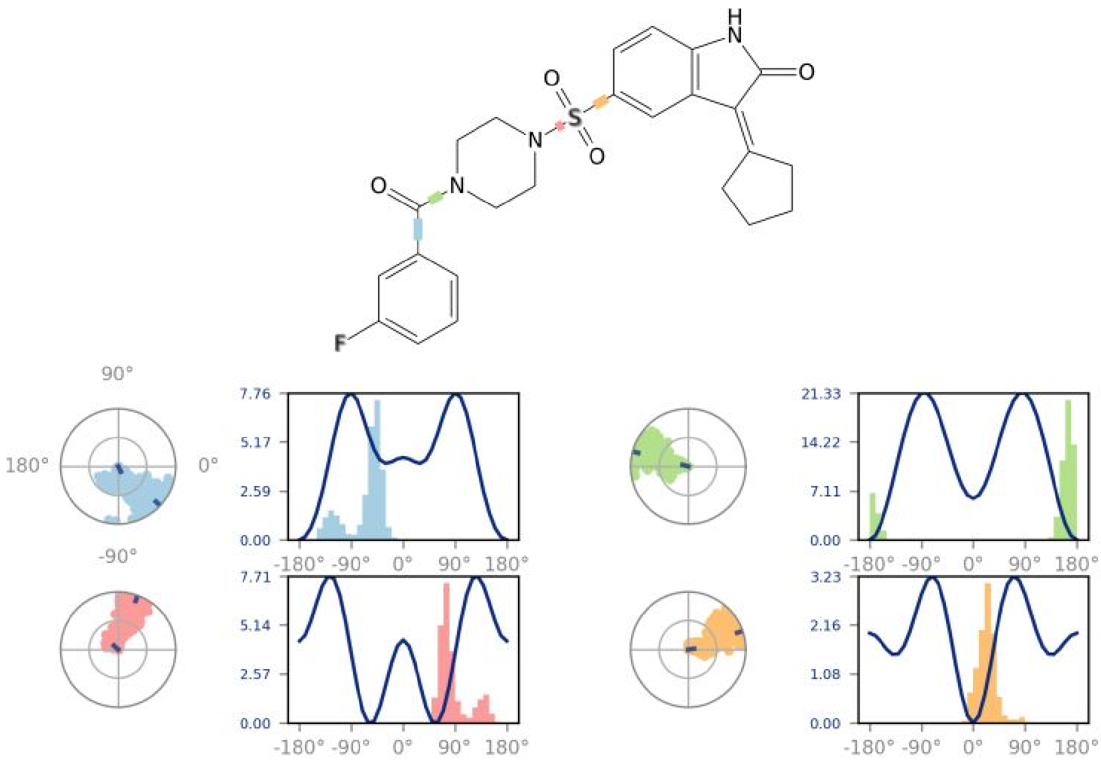

Figure S13. Torsional analysis of compound **9g** conformations during 20 ns simulations.

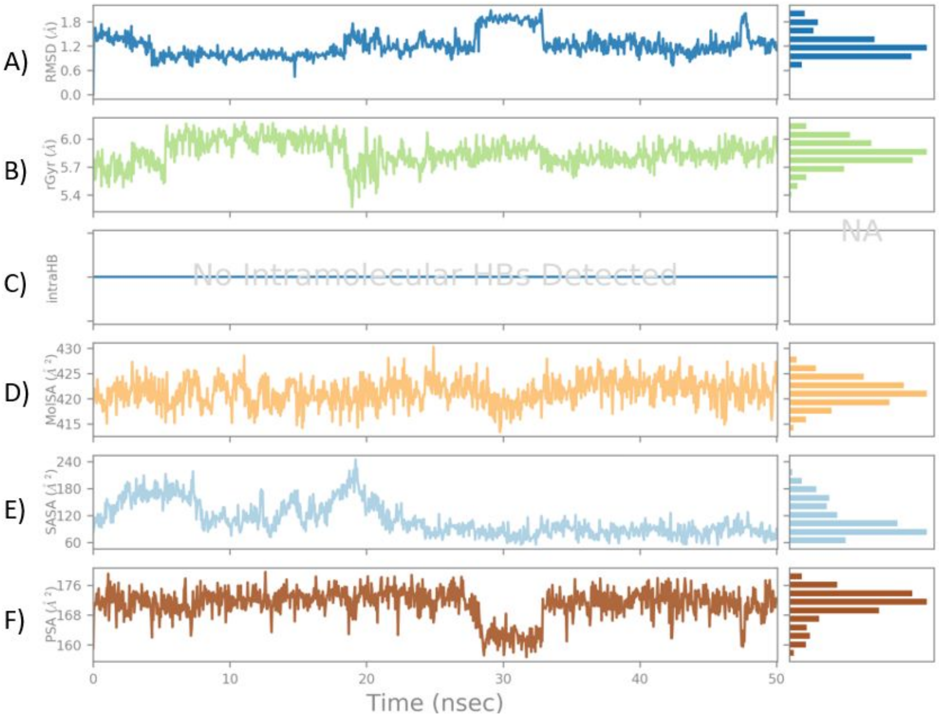

Figure S14. Ligand properties during 50 ns simulations for compound **9f**: (A) ligand RMSD, root mean square deviation, (B) radius of gyration (rGyr), (C) Intramolecular hydrogen bonds, (D) molecular surface area (MolSA), (E) solvent accessible surface area (SASA), and (F) polar surface area (PSA).

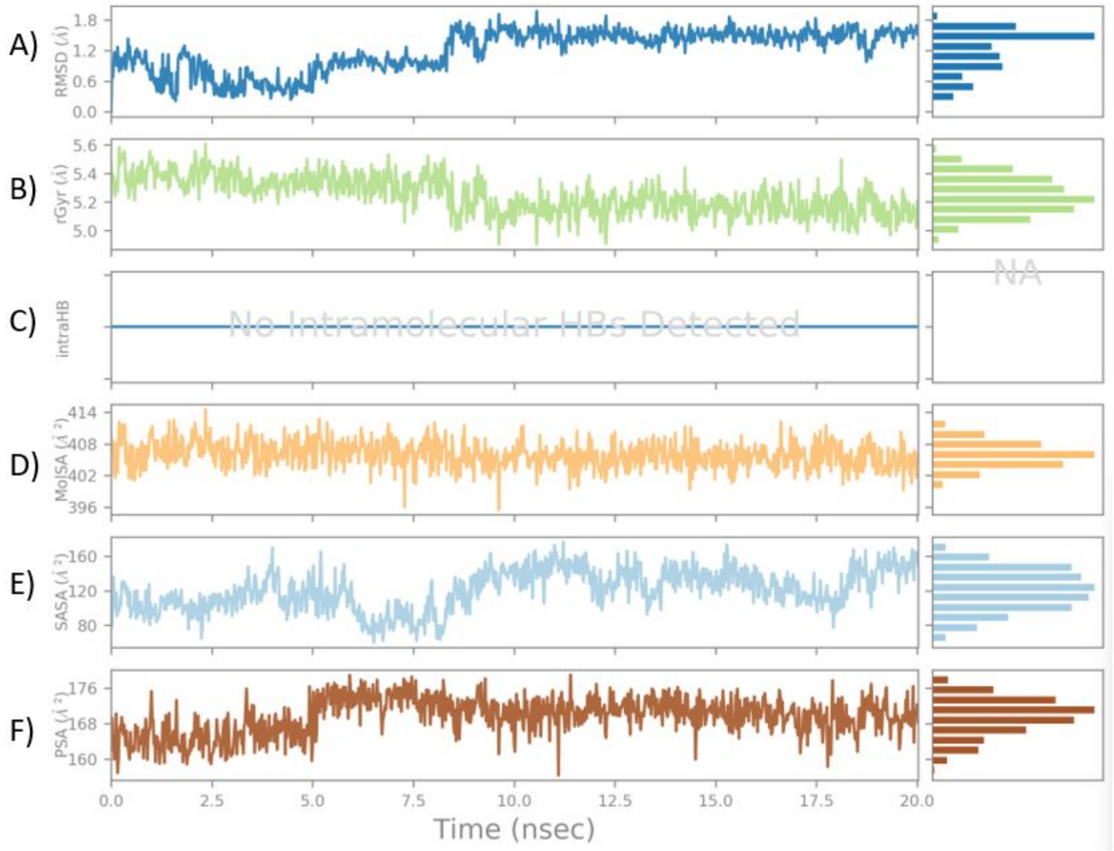

Figure S15. Ligand properties during 20 ns simulations for compound **9g**: (A) ligand RMSD, root mean square deviation, (B) radius of gyration (rGyr), (C) Intramolecular hydrogen bonds, (D) molecular surface area (MolSA), (E) solvent accessible surface area(SASA), and (F) polar surface area (PSA).

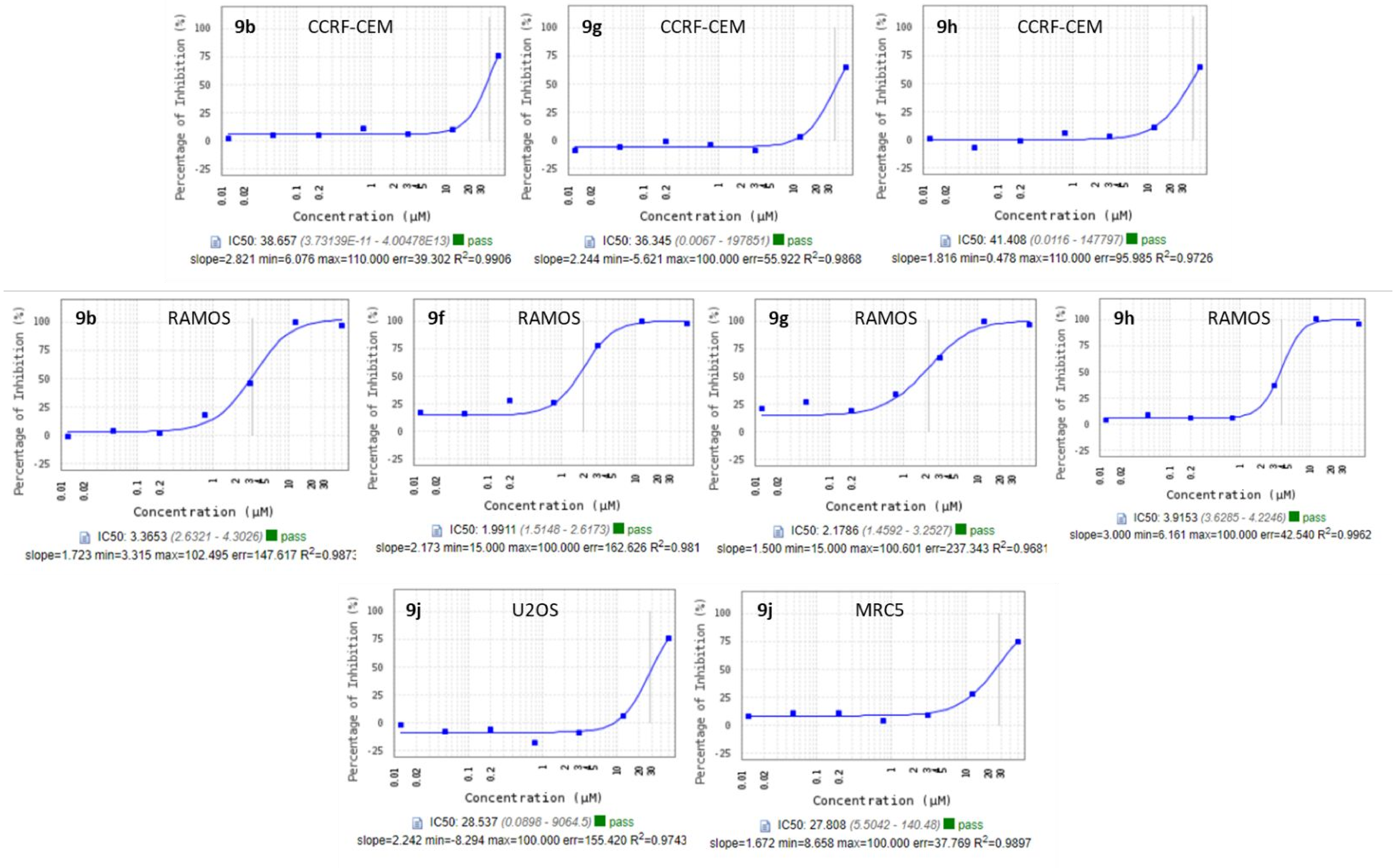

Figure S16. Representative dose-response curves of compounds **9b**, **9f**, **9g**, **9h** and **9j**.

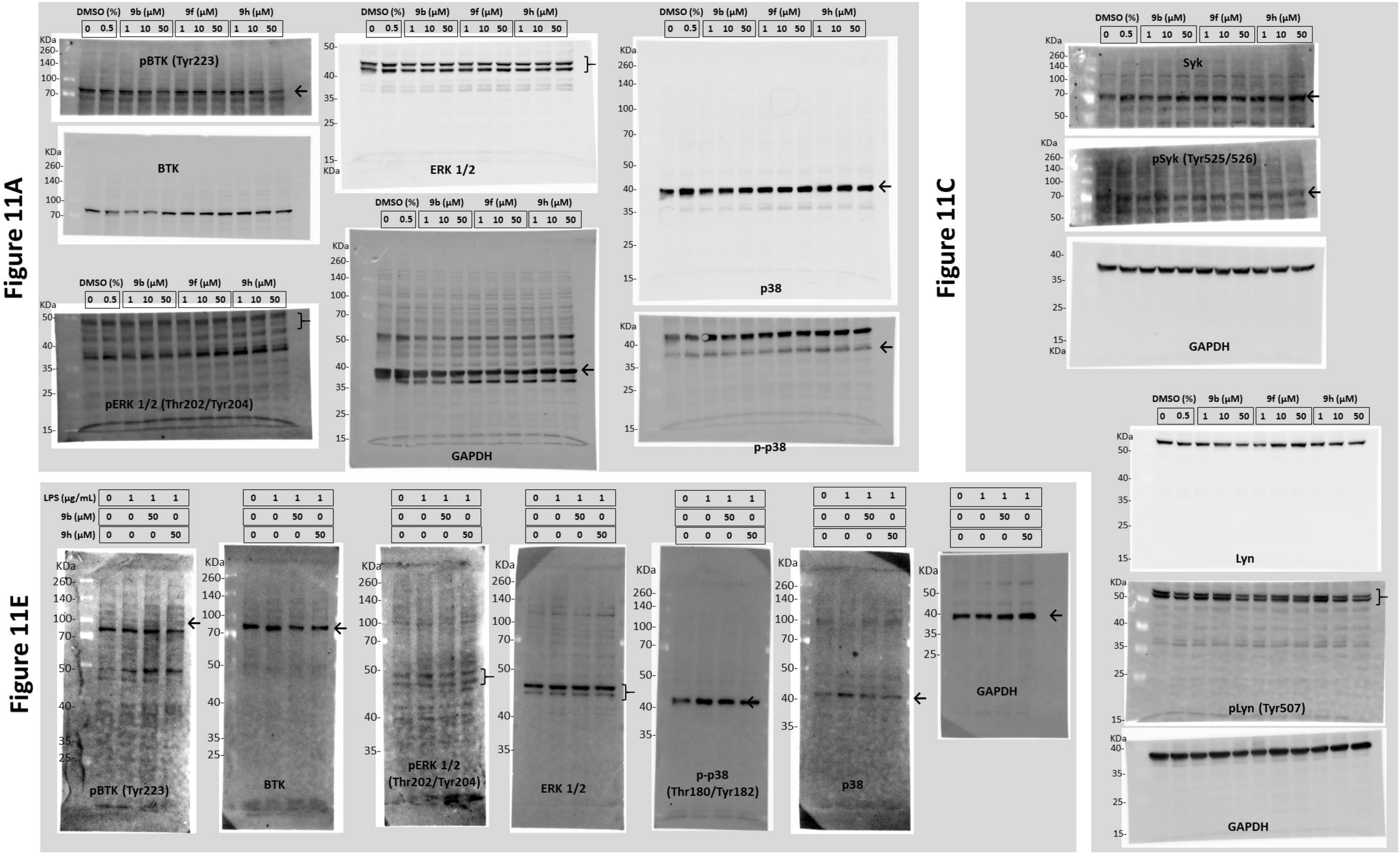

Figure S17. Original images of uncropped blots that were presented in main **Figure 11** are shown here. Note that blots were horizontally cut for some experiments, and each separate blot was stained with different primary antibodies.
